# Phlebotomine sand flies (Diptera: Psychodidae) of the Maghreb region: A systematic review of distribution, morphology, and role in the transmission of the pathogens

**DOI:** 10.1371/journal.pntd.0009952

**Published:** 2022-01-06

**Authors:** Kamal Eddine Benallal, Rafik Garni, Zoubir Harrat, Petr Volf, Vít Dvorak

**Affiliations:** 1 Laboratory of Parasitic Eco-Epidemiology and Genetic of Populations, Institut Pasteur of Algiers, Algiers, Algeria; 2 Department of Parasitology, Faculty of Science, Charles University, Prague, Czech Republic; Vienna, AUSTRIA

## Abstract

**Background:**

Phlebotomine sand flies (Diptera: Psychodidae) are important vectors of various human and animal pathogens such as *Bartonella bacilliformis*, *Phlebovirus*, and parasitic protozoa of the genus *Leishmania*, causative agent of leishmaniases that account among most significant vector-borne diseases. The Maghreb countries Mauritania, Morocco, Algeria, Tunisia, and Libya occupy a vast area of North Africa and belong to most affected regions by these diseases. Locally varying climatic and ecological conditions support diverse sand fly fauna that includes many proven or suspected vectors. The aim of this review is to summarize often fragmented information and to provide an updated list of sand fly species of the Maghreb region with illustration of species-specific morphological features and maps of their reported distribution.

**Materials and methods:**

The literature search focused on scholar databases to review information on the sand fly species distribution and their role in the disease transmissions in Mauritania, Morocco, Algeria, Tunisia, and Libya, surveying sources from the period between 1900 and 2020. Reported distribution of each species was collated using Google Earth, and distribution maps were drawn using ArcGIS software. Morphological illustrations were compiled from various published sources.

**Results and conclusions:**

In total, 32 species of the genera *Phlebotomus* (*Ph*.) and *Sergentomyia* (*Se*.) were reported in the Maghreb region (15 from Libya, 18 from Tunisia, 23 from Morocco, 24 from Algeria, and 9 from Mauritania). *Phlebotomus mariae* and *Se. africana* subsp. *asiatica* were recorded only in Morocco, *Ph. mascitti*, *Se. hirtus*, and *Se. tiberiadis* only in Algeria, whereas *Ph. duboscqi*, *Se. dubia*, *Se. africana africana*, *Se. lesleyae*, *Se. magna*, and *Se. freetownensis* were reported only from Mauritania. Our review has updated and summarized the geographic distribution of 26 species reported so far in Morocco, Algeria, Tunisia, and Libya, excluding Mauritania from a detailed analysis due to the unavailability of accurate distribution data. In addition, morphological differences important for species identification are summarized with particular attention to closely related species such as *Ph. papatasi* and *Ph. bergeroti*, *Ph. chabaudi*, and *Ph. riouxi*, and *Se. christophersi* and *Se. clydei*.

## Introduction

Phlebotomine sand flies (Diptera: Psychodidae) are small insects with nocturnal activity, females being hematophagous and feeding on various vertebrate hosts depending on species [1,2]. They live in various habitats, some species thriving in the vicinity of human dwellings and shelters of domestic animals that provide favorable humidity and temperature conditions and breeding sites as their larval stages are terrestrials, living in microhabitats with organic material [1,2]. So far, over 950 described species were classified into several genera, approximately 100 species in both Old and New World are incriminated in transmission of various pathogens including those infecting humans: bacteria *Bartonella bacilliformis*, sand fly–borne viruses, and, most importantly, parasitic protozoa of the genus *Leishmania* [3], causative agents of human and veterinary diseases known as leishmaniases that constitute a major health problem, threatening more than 350 million people in many countries and approximately 2 million of new cases reported each year [4].

Regarded as neglected diseases, leishmaniases have a huge impact on affected countries, challenging their public health services, exacerbating poverty, decreasing workers productivities, and threatening various age groups including children [5]. In 2018, of the 200 countries and territories that reported data to WHO, 97 (49%) were considered endemic and 4 countries having previously reported cases of leishmaniasis [6]. The diseases occur in 4 main clinical forms, cutaneous leishmaniasis (CL), mucocutaneous form (MCL), visceral leishmaniasis (VL) also known as kala-azar, and post–kala-azar dermal leishmaniasis (PKDL). These clinical forms, ranging from self-healing but potentially disfiguring skin lesion to serious visceral disease that is potentially life threatening when untreated, are typically associated with particular *Leishmania* species; however, the actual clinical outcome depends on multiple factors including the immunocompetence of the patient. The annual incidence of leishmaniasis is estimated at around 0.2 to 0.4 million cases for VL and from 0.7 to 1.2 million cases for CL [7].

**Fig 1:**
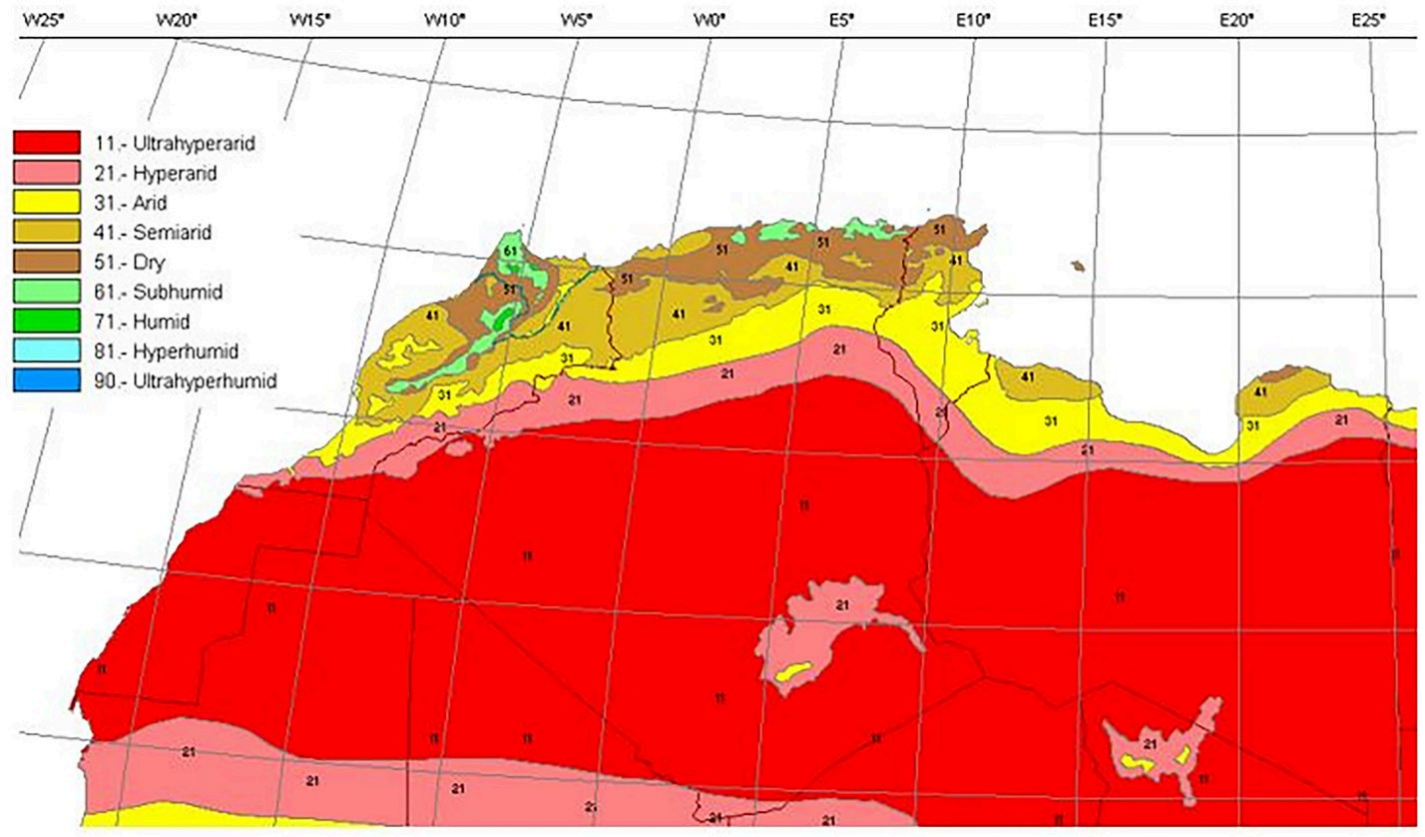
Bioclimatic stages of the Maghreb region.

The Maghreb region is located between 19° and 37° N and 15W° and 25°E, and it covers an area of 6,045,741 km^2^ including Mauritania, Morocco, Algeria, Tunisia, and Libya. In such a vast region, different bioclimatic zones occur ranging from humid to the hyperarid (Fig 1). Leishmaniases are known as dynamic diseases influenced by various factors [8]. Subsequently, between 2004 and 2008, 355 VL and 58,651 CL cases were reported in the Maghreb region [7]. Algeria is ranked first in CL cases and second worldwide after Afghanistan, reporting 44,050 cases, followed by Tunisia (7,631), Libya (3,540), and Morocco (3,430) cases. VL is less frequent, Morocco is ranked first in VL, reporting 152 cases, followed by Algeria with 111, Tunisia with 89 cases, and Libya with 3 cases for the same period [9]. Despite published VL and CL cases in the Maghreb region, the data are compromised by expected underreporting [5].

These high numbers were the consequence of several human and natural factors such as the looseness of the sand fly control campaigns, strong urbanization of the rural and suburban areas, and changes in land use that stimulate the expansion or migration of the reservoir hosts such as *Psammomys obesus* from its natural habitats caused by hydric stress and the decrease in food sources; all these factors led to the emergence of new foci of transmission as reported in Algeria [10,11]. Thus, synergic effects of globalization, climatic change, and various human activities allow the parasites and their vectors to spread in space and time as it was noticed in Europe [8].

Sand flies belong to the order Diptera, suborder Nematocera, family Psychodidae, and subfamily Phlebotominae. In the Old World, sand flies are traditionally divided into 7 genera, some of these comprising further subgenera. Sand fly taxonomy is traditionally based on analysis of decisive morphological characters on the head and genitalia [3,12], but the advent of molecular techniques that deploy mainly sequencing analyses of suitable genetic markers [13] provided further insights into cryptic species including challenging taxa of the Maghreb region like *Phlebotomus perniciosus* and *Ph. longicuspis* or *Ph. riouxi* and *Ph. chabaudi*. Moreover, they allowed to associate males with females, e.g., *Chinius samarensis* [14], to give an insight about vectors and nonvectors by the detection of DNA parasite or to propose evolutionary systematic [13,15,16]. In addition, next-generation sequencing (NGS) assays and statistical analysis of the subsequent reads were used to help species identification within pooled samples in several virological studies [14–16]. Recently, matrix-assisted laser desorption/ionization time-of-flight mass spectrometry (MALDI-TOF MS), a protein-based method, was introduced for rapid and cost-effective species identification of various arthropods including sand flies upon their species-specific protein profiles [17]. These molecular approaches complement the morphological identification that is still regarded as the golden standard and the backbone of entomological surveys and vector control programs [18].

The incrimination of particular sand fly species as a vector of leishmaniasis is based on assessment of several criteria previously established [1,4,19]. As a complete evaluation of all these criteria requires a sustained field and experimental research, beside sand fly species fully proven as leishmaniasis vectors, many other species are considered as suspected vectors based on circumstantial evidence and partial fulfillment of some of these criteria [15,20]. In the Maghreb region, the omnipresence of many proven and suspected vectors of several *Leishmania* species supports transmission cycles of both CL and VL [21]. While vast knowledge of sand fly species present in Maghreb countries was accumulated during several decades of focused research including precise morphological analysis of some species, the information remain fragmented, albeit it could serve as a valuable background for advanced molecular studies. The correct and conclusive morphological identification requires accurate and updated identification keys and experienced entomologists who can provide sufficient expertise for evaluation of often minute morphological characters.

This review aimed to update the list of sand fly species so far recorded in the Maghreb region (Morocco, Algeria, Tunisia, and Libya) providing (i) their presumable geographic distribution with respect to the bioclimatic zones; (ii) their proven or suspected role in pathogen transmission in the Maghreb region (Table 1); and (iii) defining morphological characters that enable a species identification with special attention to taxonomically challenging species such as *Ph*. *chabaudi/Ph*. *riouxi*, *Ph. papatasi/Ph. bergeroti*, or *Ph*. *perniciosus* species complex.

**Table 1:** Sand fly species their distribution and vectorial role in the Maghreb region.

Genus	Subgenus	Species	Figure		Map of distribution	Country of distribution			
			Female	Male		M	A	T	L
*Phlebotomus*	*Phlebotomus*	*Ph*. papatasi*^x^	4	6	2	X	X	X	X
		*Ph*. *bergeroti*	5	7	3	X	X	X	X
	*Paraphlebotomus*	*Ph*. sergenti*^x^	8	9	10	X	X	X	X
		*Ph*. *alexandri*	11	12	13	X	X	X	X
		*Ph*. *kazeruni*	14	15	16	X	X		
		*Ph*. *chabaudi*	18	17	19	X	X	X	X
		*Ph*. *riouxi*	21	22	20	X	X	X	X
	*Larroussius*	*Ph*. perniciosus*^x^	25	23	26	X	X	X	X
		*Ph*. *perniciosus* atypical	-	24	27	X	X	X	
		*Ph*. longicuspis**^x^	29	28	30	X	X	X	X
		*Ph*. perfiliewi*	32	31	33	X	X	X	
		*Ph*. ariasi*^x^	35	34	36	X	X	X	X
		*Ph*. *chadlii*	37	38	39	X	X	X	
		*Ph*. langeroni*	41	40	42	X	X	X	X
		*Ph*. *mariae*	-	43	44	X			
	*Transphlebotomus*	*Ph*. *mascittii*	45	46	47		X		
*Sergentomyia*	*Sergentomyia*	*Se*. *minuta*	48	49	50	X	X	X	X
		*Se*. *fallax*	51	52	53	X	X	X	X
		*Se*. *antennata*	55	54	56	X	X	X	X
		*Se*. *cincta*	57	-	58		X		
		*Se*. *schwetzi*	59	60	61	X	X	X	X
	*Parrotomyia*	*Se*.*africana* subsp. *asiatica*	63	-	62	X			
		*Se*. *africana* subsp. *eremitis*	66	65	64		X		
		*Se*. *lewisi*	68	69	67	X	X	X	X
	*Grassomyia*	*Se*. *dreyfussi*	72	71	70	X	X	X	X
	*Sintonius*	*Se*. clydei**	75	74	73	X	X	X	X
		*Se*. *christophersi*	78	77	76	X	X	X	X
		*Se*. *hirtus*	-	80	79		X		
		*Se*. *tiberiadis*	83	82	81		X		

*Proven vector of the parasites in the Maghreb region.

**Detection of *Leishmania* DNA.

^**x**^Detection of *Phlebovirus* RNA.

X, occurrence.

A, Algeria; L, Libya; M, Morocco; T, Tunisia.

## Materials and methods

### Literature search and data extraction

For our study, we have followed PRISMA-P protocol [22]. We consulted all available data on sand fly fauna of the Maghreb regions, namely Mauritania, Morocco, Algeria, Tunisia, and Libya. Various databases like PubMed, Google Scholar, Archive de Institut Pasteur d’Algérie (IPA), and Archive de Institut Pasteur de Tunis were searched for available data from the period 1900 to 2020. The available English and French resources published in full text, articles, reports, congress presentations, book chapters, and thesis dissertations containing information on sand flies were added, treated, and analyzed together with data available from the Laboratoire d’Eco-épidémiologie Parasitaire et Génétiques des Populations of Institut Pasteur of Algeria. The following search string was used: terms in title: [(phlebotomine OR sand flies OR sand flies) AND in all fields: (*Phlebotomus* OR *Sergentomyia*) AND in all fields: (*Phlebotomus* OR *Paraphlebotomus* OR *Larroussius* OR *Transphlebotomus* OR *Sergentomyia* OR *Parrotomyia* OR *Grassomyia* OR *Sintonius*) AND in all fields: (species name) AND in all fields: (distribution OR presence OR occurrence OR report OR spread OR disperse OR detect) AND in all fields:(Algeria OR Libya OR Tunisia OR Maghreb region OR Morocco) AND in all fields: (leishmaniasis OR “cutaneous leishmaniasis” OR “CL” OR “visceral leishmaniasis” OR “VL” “canine leishmaniasis” OR “CanL” OR “Phlebovirus” OR “Bunyaviridae”) AND in all fields: (“neglected tropical diseases” OR “vector- borne diseases”) AND in all fields: (“vector control”)] as suggested by Dvorak and colleagues [23].

Titles relevant to the scope of this review were obtained in full text and selected for inclusion. Beside these, some data also come from direct consultations with experts and their in-house unpublished databases.

The distribution of Moroccan sand fly species was reported in [24–27], Algerian species [11,28–35], Tunisian species [36–38], and Libyan species [39–42]. For each sand fly species, geographical positions of published records (S1 Data) were first gathered in Google Earth software from sourced publications and then processed with ArcGIS Pro software version 2.5.0 for analysis and maps drawing. The bioclimatic zones classification (Fig 1) was based on the Worldwide Bioclimatic Classification System [43].

We adopted the abbreviation for the genus and subgenus according to the last suggestions of the experts during the International Symposium on Phlebotominae Sand Flies (ISOPS) 2016 meeting and the designation “Sand fly” or “Sand flies” along this review since during the same meeting, the experts did not converge to a common designation [44].

### Morphological analysis

Morphological illustrations were compiled from the following publications and books: Abonnenc [2], Dedet and colleagues [33], Bailly-Choumara and colleagues [24], Rioux and colleagues [25,26,45–47], Annajar [41], Depaquit and colleagues [48], Léger and colleagues [49,50], Parrot and colleagues [51–53], Croset and colleagues [54,55], El Sawaf and colleagues [56], Chamkhi and colleagues [57], and Theodor and colleagues [58].

## Results

### I- Genus *Phlebotomus*

The genus *Phlebotomus* is defined by the usual absence of cibarial teeth and pigmented patch. The hind end of the abdomen tergites 2 to 6 holds many erected setae that arise from large sockets of the same size as those on the tergite. In Maghreb, the species belong to 4 subgenera: *Phlebotomus* Rondani, 1843, *Paraphlebotomus* Theodor, 1948, *Larroussius* Nitzulescu 1931, and *Transphlebotomus* Artemiev 1984. In males, the styles bear either 3 terminal and 2 subterminal, rather short spines (subgenus *Phlebotomus*) or 2 long terminal and 2 or 3 subapical spines without accessory spine (other subgenera). In females, the spermathecae have superficial striation or are annulated with basal structures in some species depending of the subgenus [2,12,59]. The main differences between the species are summarized in **Tables A and B in** S1 Text for males and females, respectively.

### I-1 Subgenus *Phlebotomus* Rondani, 1843

The species belonging to this subgenus are recognized by (i) large male genitalia with 5 short spines (3 spatulated terminal spines and 2 at the mid-style position) upon a long cylindrical style; (ii) a paramere with 2 lengthy secondary appendices; (iii) short and conical aedeagus; and (iv) a small button at the base of the coxite bearing few long setae. Females possess pharynx armed with large teeth fringed with minute denticules and spermathecae with uniform shaped segments which are ended by a terminal knob. In Maghreb, 2 species of this subgenus were recorded: *Ph*. *papatasi* (Scopoli, 1786) and *Ph*. *bergeroti* (Parrot, 1934).

### I-1-1 *Phlebotomus (Phlebotomus) papatasi*, Scopoli, 1786

**Fig 2:**
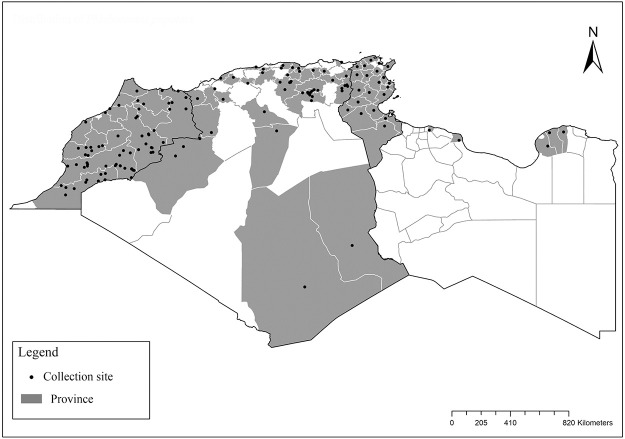
Distribution of *Phlebotomus papatasi*. Available from: https://services3.arcgis.com/W1gaXmEpGR8h1K59/arcgis/rest/services/maghreb/FeatureServer.

It is one of most studied species due to its large geographical area of occurrence and its medical importance as it is the principal vector of the zoonotic cutaneous leishmaniasis (ZCL) in highland and north of Sahara caused by *Leishmania major* [60]. Except the zymodeme MON-269 from Algeria, all isolates of *L*. *major* in the region belong to zymodeme MON-25 [61,62]. This species also has been reported to transmit the Sicilian virus in Egypt [63] and found infected by Toscana virus (TOSV) in Morocco [64]. It has a vast distribution from northern to the southern part of the Maghreb region (Algeria, Morocco, Tunisia, and Libya) with a high abundance in the highlands and north of Sahara between 27°/35° N and −4°/58° E worldwide [65]. It is encountered from the humid to ultra-hyperarid bioclimatic zones (Fig 2).

### I-1-2 *Phlebotomus bergeroti* Parrot, 1934

**Fig 3:**
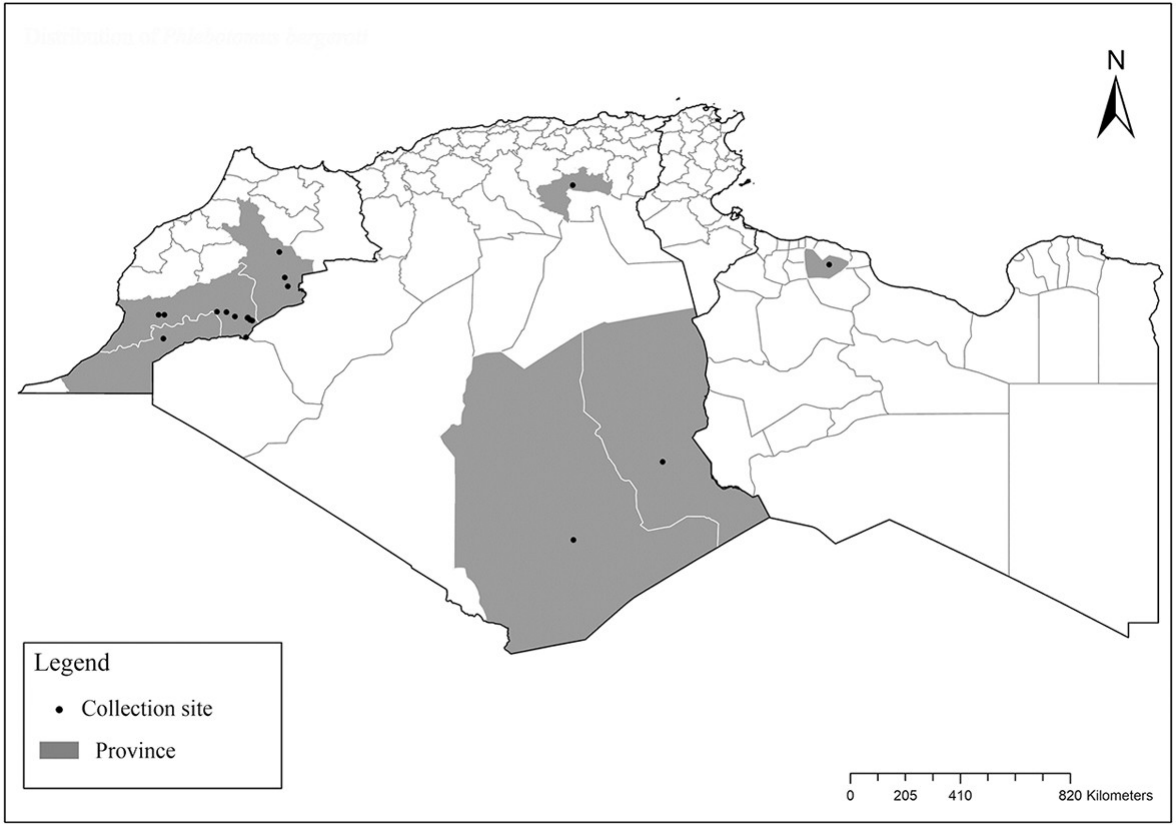
Distribution of *Phlebotomus bergeroti*. Available from: https://services3.arcgis.com/W1gaXmEpGR8h1K59/arcgis/rest/services/maghreb/FeatureServer.

**Fig 4:**
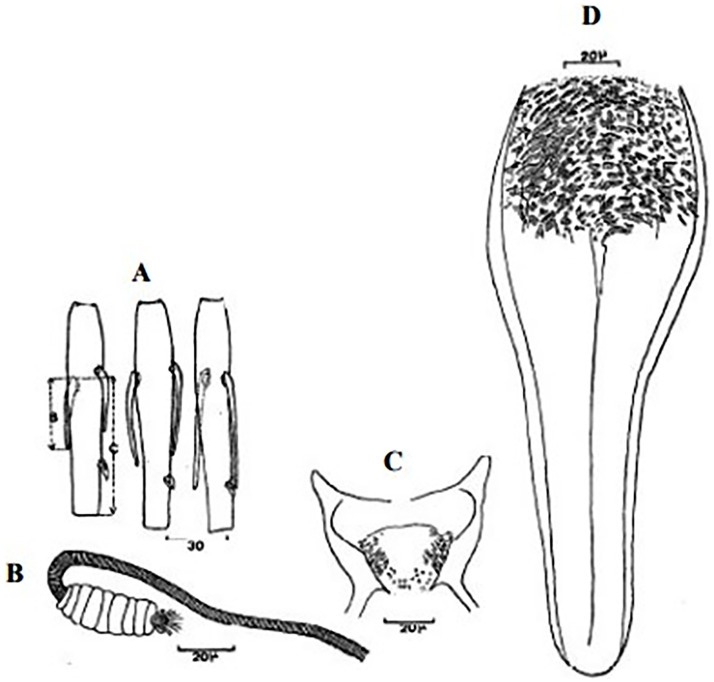
*Phlebotomus papatasi* ♀ [2]. **(A)** Fourth antenna segment. **(B)** Spermathecae. **(C)** Cibarium. **(D)** Pharynx.

**Fig 5:**
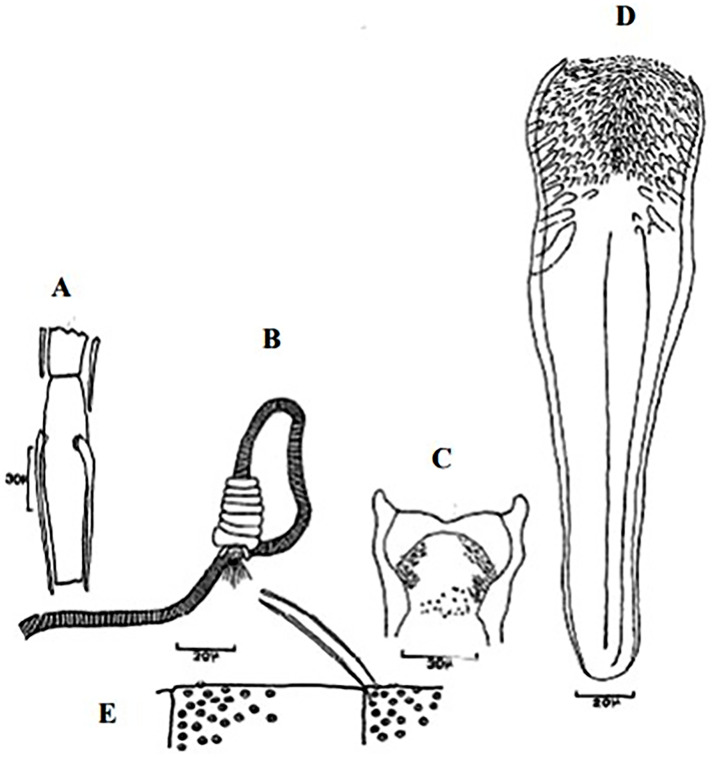
*Phlebotomus bergeroti* ♀ [2]. **(A)** Fourth antenna segment. **(B)** Spermathecae. **(C)** Cibarium. **(D)** Pharynx. **(E)** Abdomen with dressed setae.

**Fig 6:**
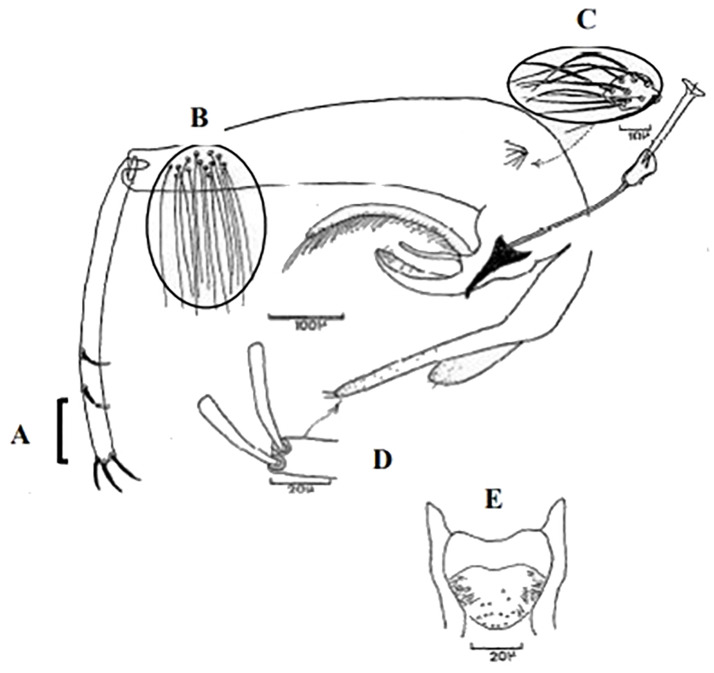
*Phlebotomus papatasi ♂* [2]. **(A)** Spines position. **(B)** Coxite setae. **(C)** Tuft of seta. **(D)** Setae of the lateral lobe. **(E)** Cibarium.

**Fig 7:**
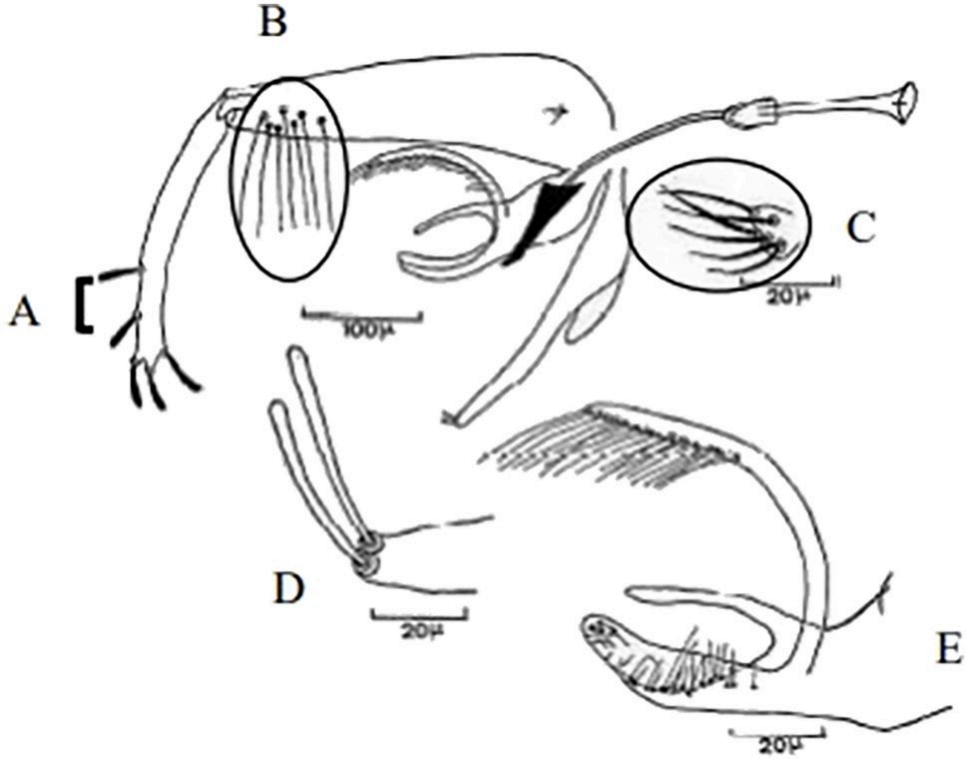
*Phlebotomus bergeroti ♂* [2]. **(A)** Spines position. **(B)** Coxite setae. **(C)** Tuft of seta. **(D)** Setae of the lateral lobe. **(E)** Paramere.

This species was shown to transmit *L*. *major* under laboratory conditions [66], and it is suspected to play a role in the transmission of this parasite in Burkina Faso, Chad, Egypt, Iran, Mauritania, Oman, and Yemen on the basis of epidemiological evidence [67]. It occurs in the southern part of the Maghreb region (Mauritania, Morocco, Algeria, and Libya), and it is encountered in the hyperarid bioclimatic zone (Fig 3). In central Sahara regions of Algeria (Tamanrasset and Illizi), *Ph*. *bergeroti* and *Ph*. *papatasi* are often confused in the overlapping areas of occurrence despite the description of distinct characters between the 2 species (**Table C in** S1 Text). For the females, 2 differences are known: Posterior part of pharynx has weakly distended shape in *Ph*. *papatasi* (Fig 4), while it has a bottle shape in *Ph*. *bergeroti* (Fig 5). The ascoids length is as long as the fourth antennal segment in *Ph*. *bergeroti* compared to *Ph*. *papatasi* where the ascoids are shorter. For the males, 4 major differences were highlighted: (i) the number of setae in the basal lobe coxite is typically 6 for *Ph*. *bergeroti* and 10 for *Ph*. *papatasi*; (ii) the length between the apical and median spines of the style is longer in *Ph*. *papatasi* (Fig 6) compared to *Ph*. *bergeroti* (Fig 7); (iii) the spines of the style are sharp and spatulated in *Ph*. *papatasi* and *Ph*. *bergeroti*, respectively; and (iv) the number of the tuft hairs of the coxite is less than 10 in *Ph*. *bergeroti* and more than 12 in *Ph*. *papatasi* [2].

### I-2 Subgenus *Paraphlebotomus* Theodor, 1948

The pharynx of the females bears large, backwardly directed teeth with smooth margins, which appear as a network. The spermathecae usually have their terminal segment much larger than the other ones. Males have characteristic lobes on the inner surface of their coxite, bearing tufts of long setae, and relatively short style with 4 long spines, 2 near the tip and 2 near the base. Species of this subgenus occur mainly in the Palearctic region, some are proven or suspected to transmit leishmaniases, typically CL due to *Leishmania tropica* [67]. In Maghreb region, 5 species of the subgenus *Paraphlebotomus* were recorded:

### I-2-1 *Phlebotomus sergenti* Parrot, 1917

**Fig 8:**
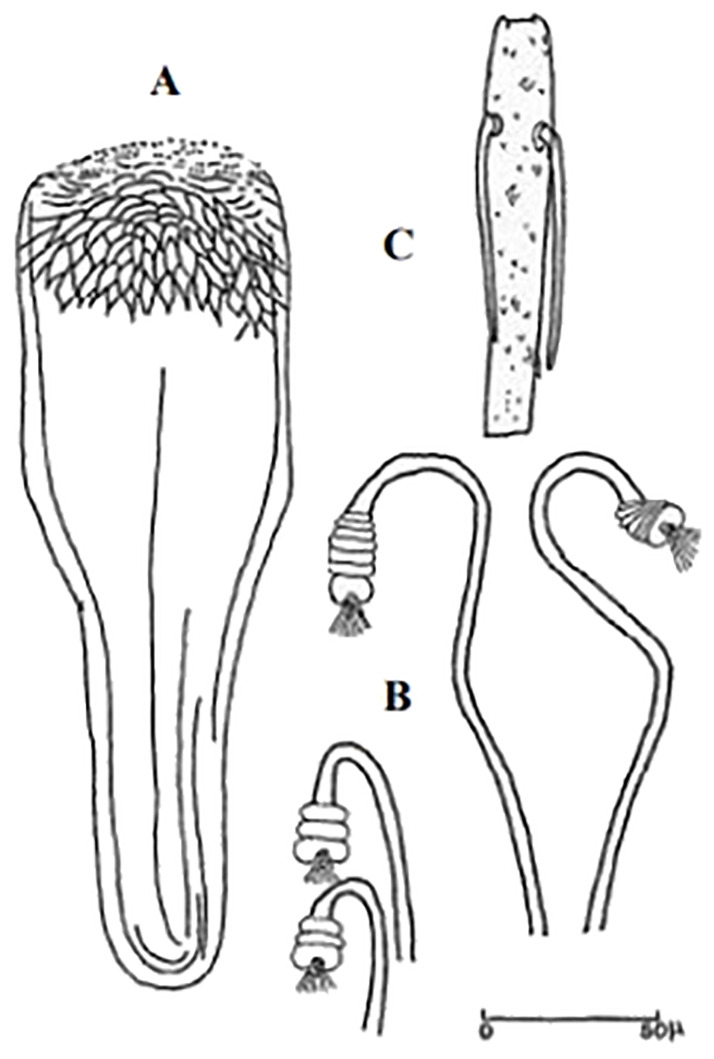
*Phlebotomus sergenti* ♀[2]. **(A)** Pharynx. **(B)** Spermathecae. **(C)** Fourth antenna segment.

**Fig 9:**
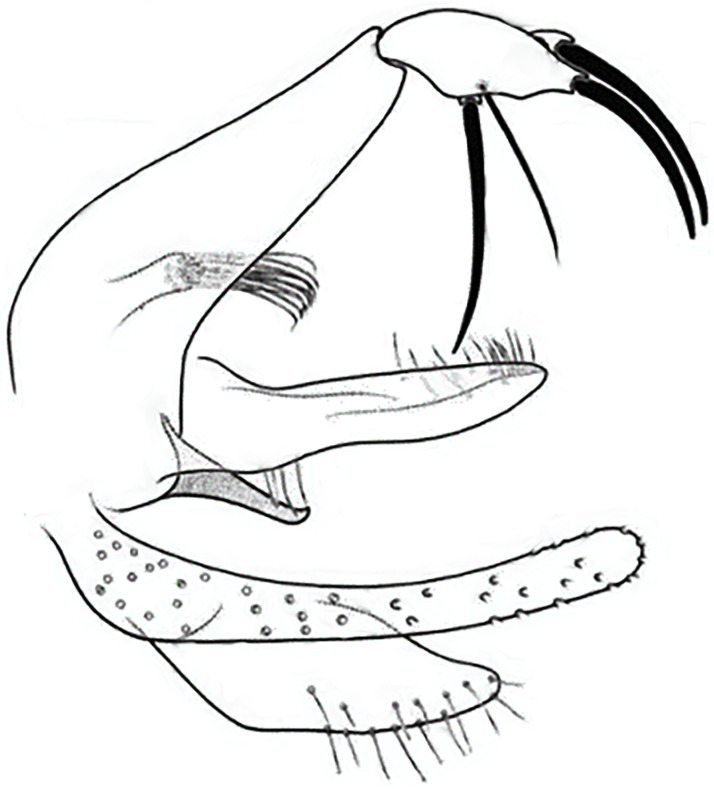
*Phlebotomus sergenti ♂* [57]. General genitalia.

**Fig 10:**
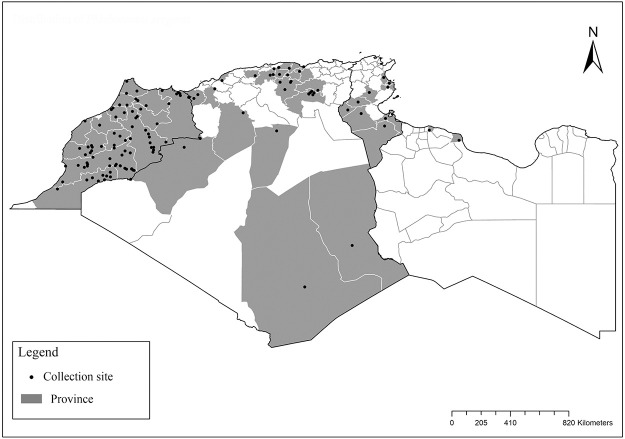
Distribution of *Phlebotomus sergenti*. Available from: https://services3.arcgis.com/W1gaXmEpGR8h1K59/arcgis/rest/services/maghreb/FeatureServer.

It is the proven vector of various zymodemes of *L*. *tropica* complex, including *L*. *killicki-*MON 301 [68–71], and suspected vector of *L*. *killicki* MON-306, recently reported in the East of Algeria [72]. Furthermore, it was found infected with TOSV in Morocco [73]. This species is collected both inside houses and outdoors, reported in all the Maghreb regions from the north to the central Sahara, occurring in the humid and arid bioclimatic zones (Figs 8–10).

### I-2-2 *Phlebotomus alexandri* Sinton, 1928

**Fig 11:**
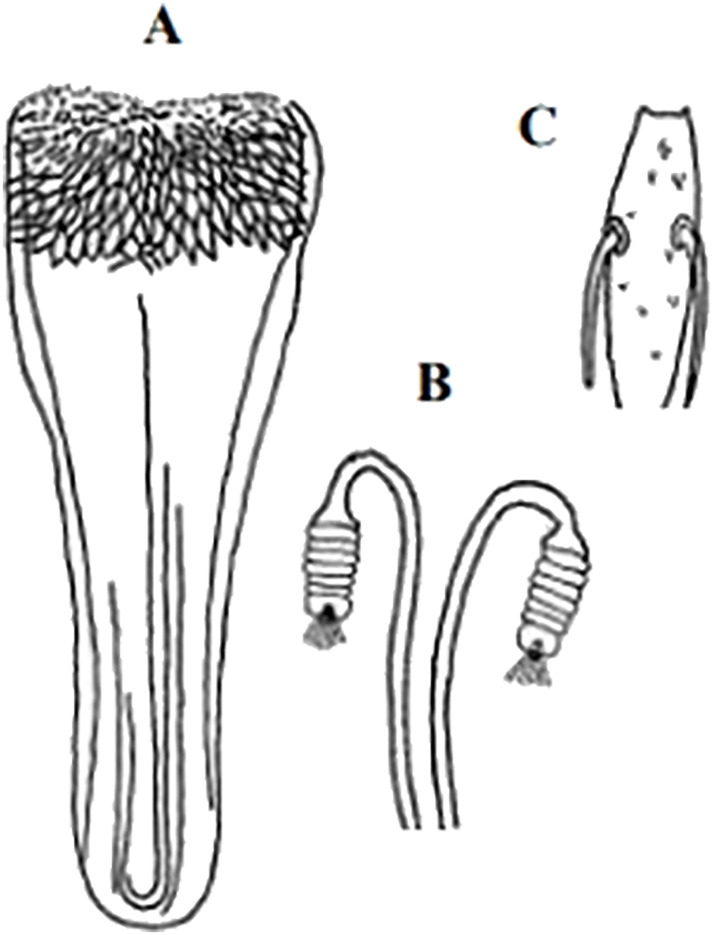
*Phlebotomus alexandri* ♀ [2]. **(A)** Pharynx. **(B)** Spermathecae. **(C)** Fourth antenna segment.

**Fig 12:**
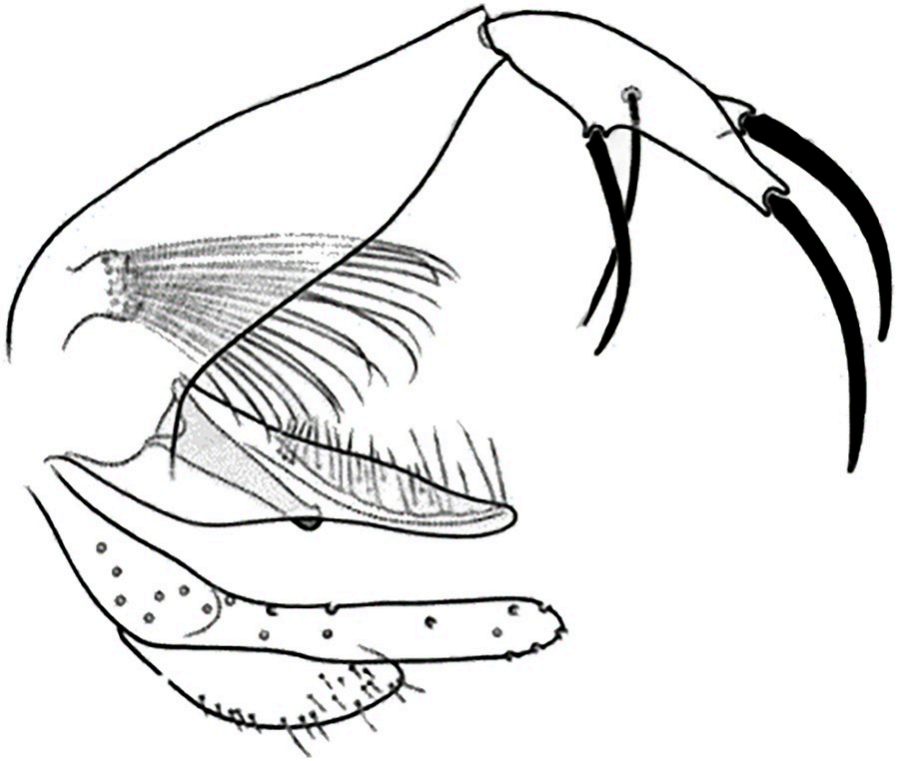
*Phlebotomus alexandri ♂* [57]. General genitalia.

**Fig 13:**
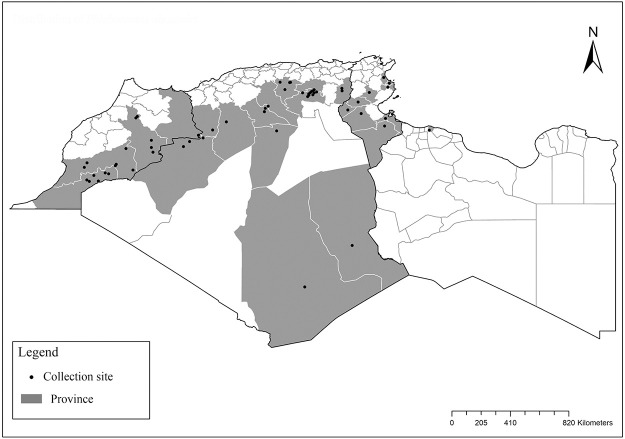
Distribution of *Phlebotomus alexandri*. Available from: https://services3.arcgis.com/W1gaXmEpGR8h1K59/arcgis/rest/services/maghreb/FeatureServer.

Despite it is the proven vector of VL in China [74], no vectorial role in the Maghreb region was so far reported. In Tunisia, Croset and colleagues [54] suspected its role in leishmaniasis cycle transmission due to its abundancy in dry and rocky biotopes (cavities and crevices dug in rocky cliffs) where rodents especially *Ctenodactylus gundi* and reptiles occur abound [33]. It was reported in entire Maghreb region, occurring from subhumid to arid bioclimatic zones (Figs 11–13).

### I-2-3 *Phlebotomus kazeruni* Theodor and Masghali, 1964

**Fig 14:**
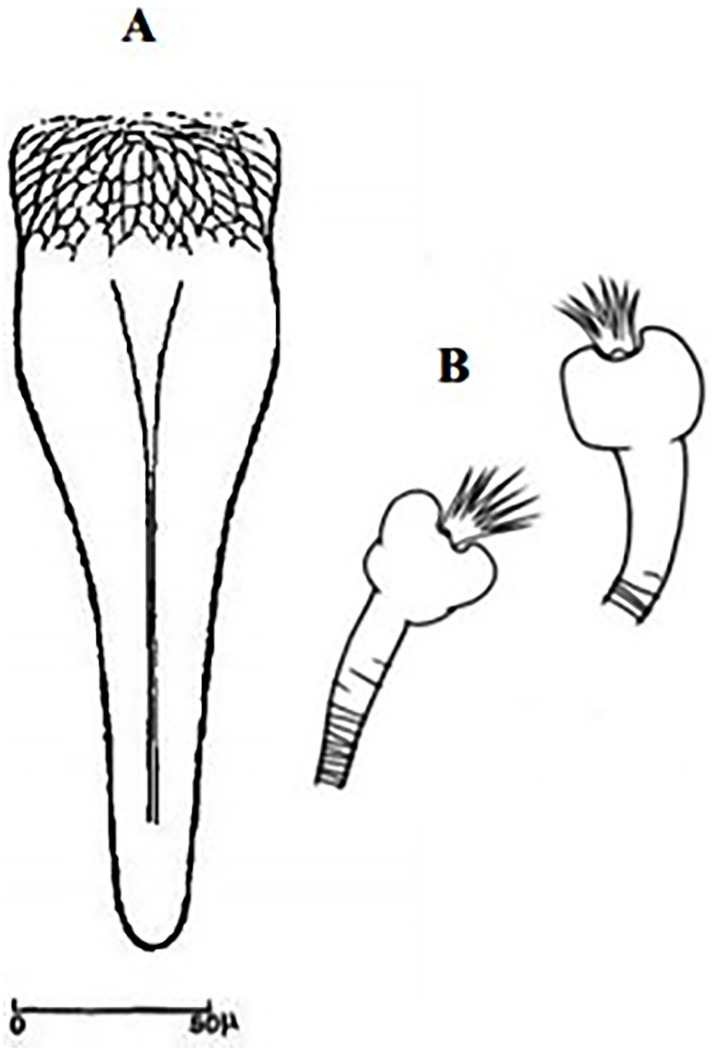
*Phlebotomus kazeruni* ♀ [29,61]. **(A)** Pharynx. **(B)** Spermathecae.

**Fig 15:**
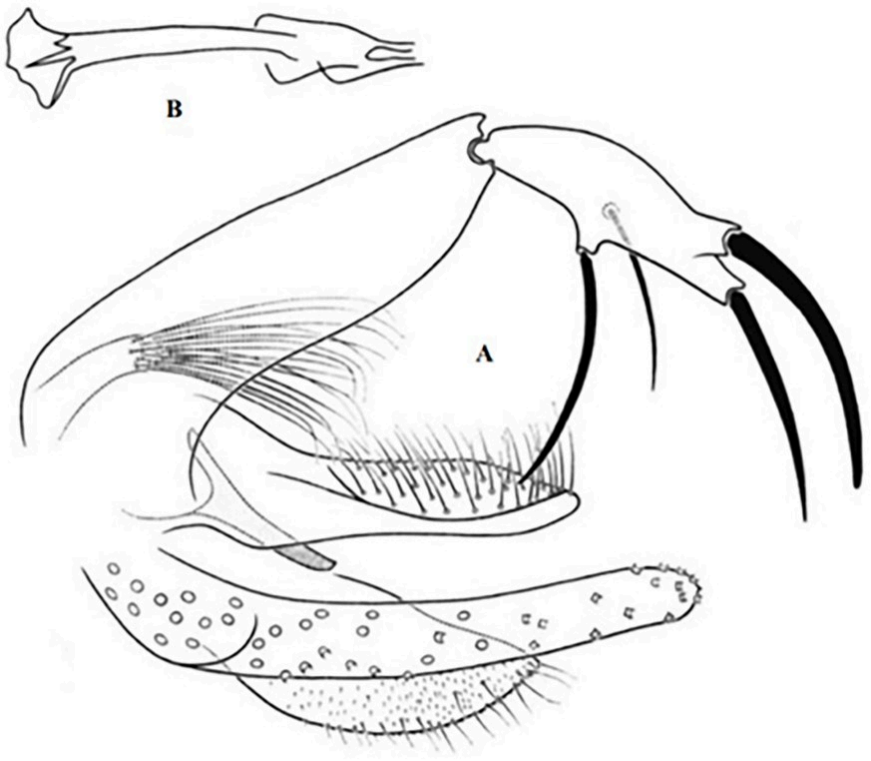
*Phlebotomus kazeruni* ♂ [29].

**Fig 16:**
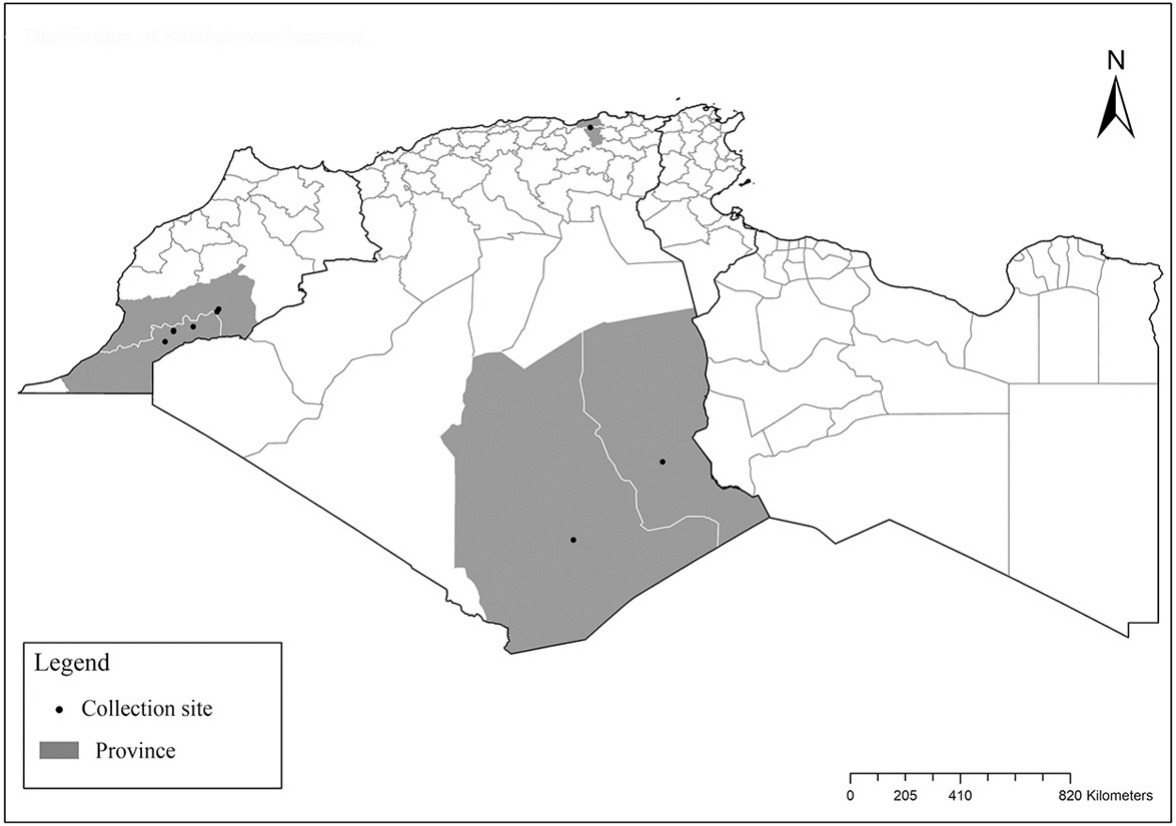
Distribution of *Phlebotomus kazeruni*. Available from: https://services3.arcgis.com/W1gaXmEpGR8h1K59/arcgis/rest/services/maghreb/FeatureServer.

This species is suspected to transmit causative agents of CL in Saudi Arabia [75] and was shown to support development of *L*. *major* under laboratory conditions [76]. It was first reported in Morocco but remains rare in Algeria where until now, few females were reported in the north in Constantine and Jijel, also in the central Sahara in Tamanrasset [26,31,77,78]. It occurs in the subhumid and arid bioclimatic zones (Figs 14–16).

### I-2-4 *Phlebotomus chabaudi* Croset, Abonnenc and Rioux, 1970

**Fig 17:**
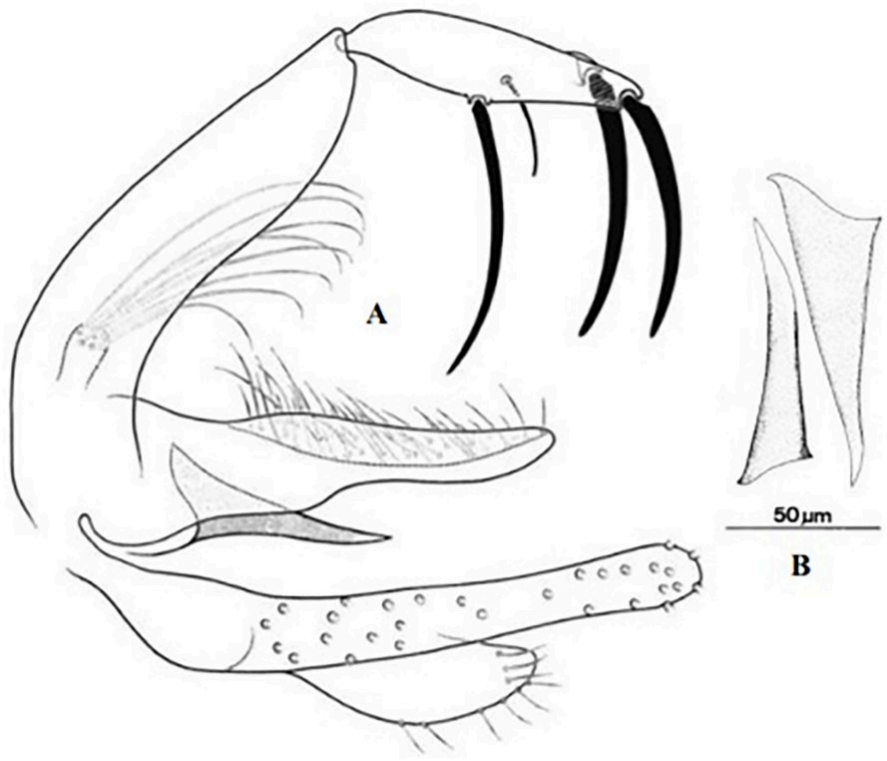
*Phlebotomus chabaudi* ♂ [51,58]. **(A)** General genitalia. **(B)** Aedeagus shape.

**Fig 18:**
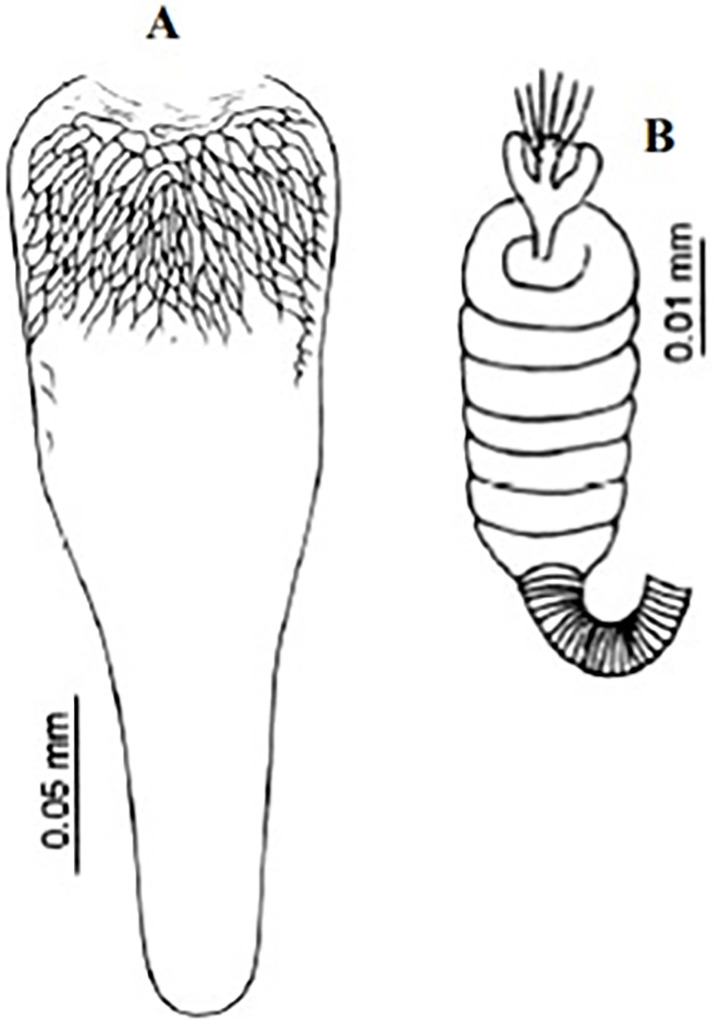
*Phlebotomus chabaudi* ♀ [44]. **(A)** Pharynx. **(B)** Spermathecae.

**Fig 19:**
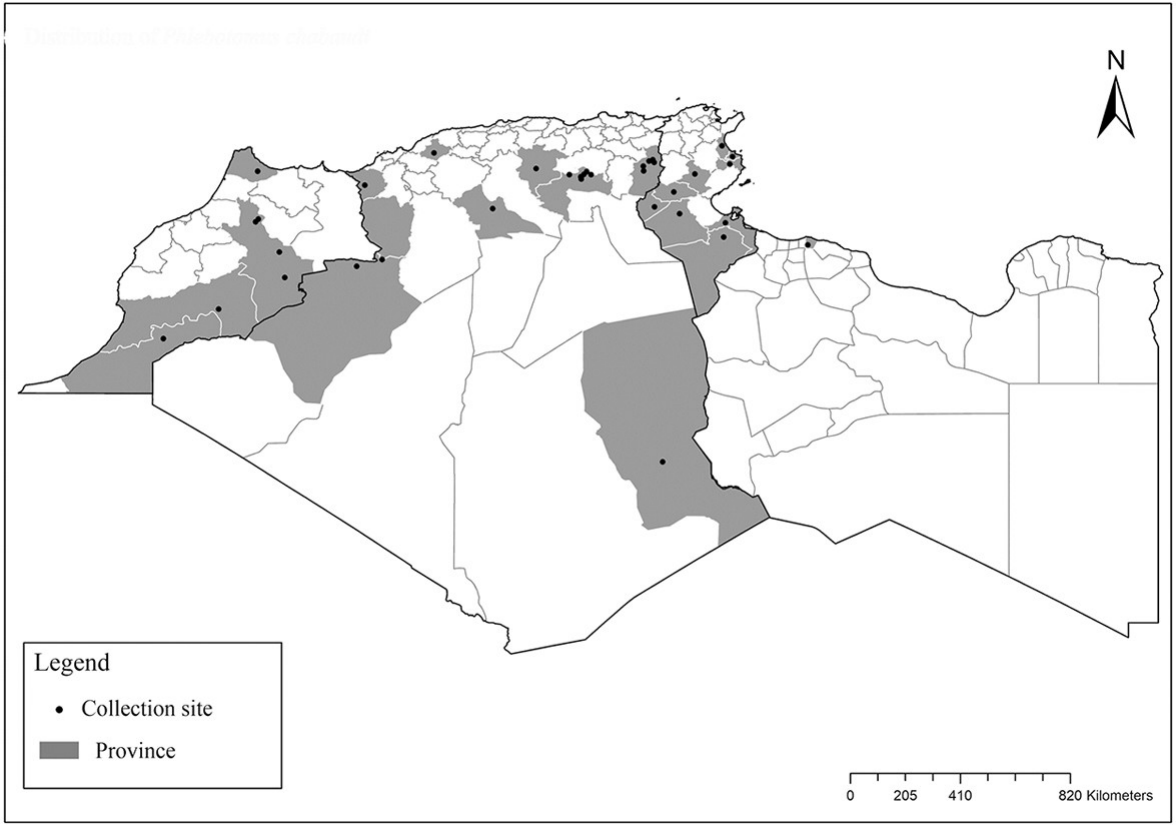
Distribution of *Phlebotomus chabaudi*. Available from: https://services3.arcgis.com/W1gaXmEpGR8h1K59/arcgis/rest/services/maghreb/FeatureServer.

So far not reported as a vector of any pathogen, it was described in 1970 based on a single male collected in Tunisia (Fig 17) [55]. In 1974, the first female (Fig 18) was collected in Algeria [79,80]. It occurs in the subhumid to arid bioclimatic zones (Fig 19).

### I-2-5 *Phlebotomus riouxi* Depaquit, Léger and Killick-Kendrick, 1998

**Fig 20:**
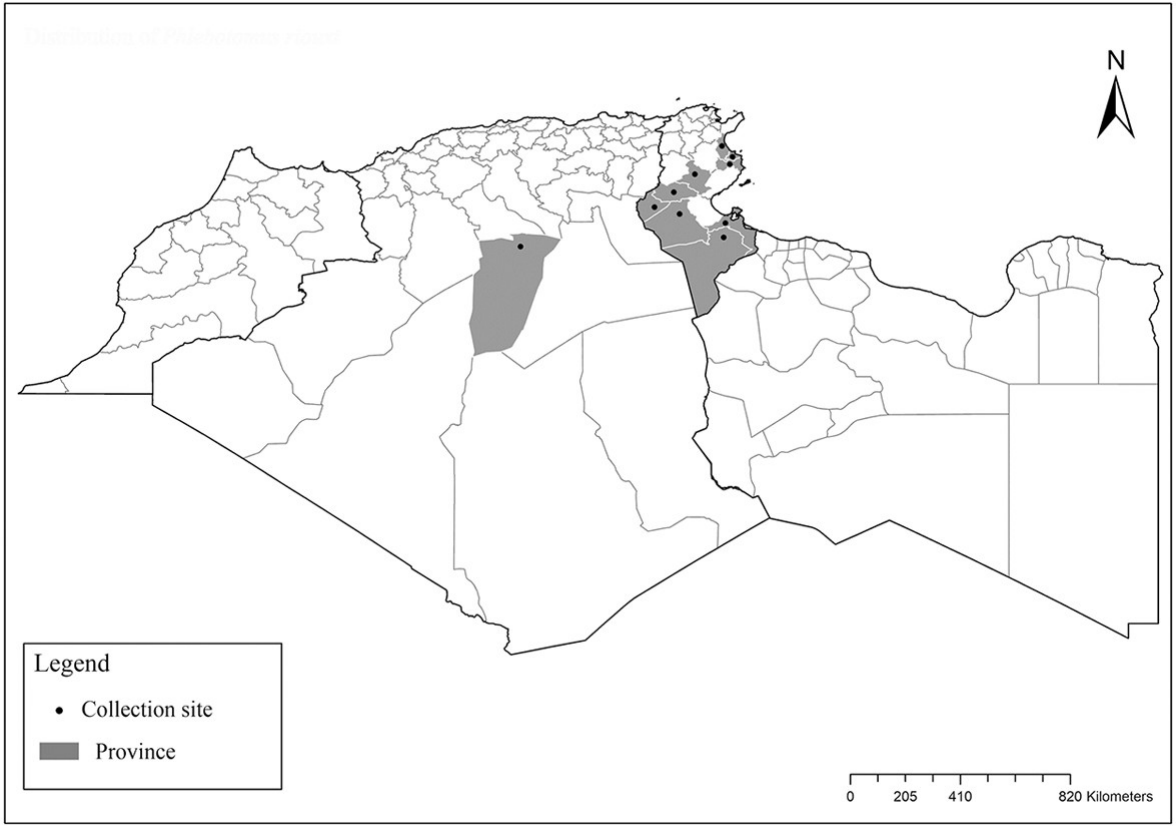
Distribution of *Phlebotomus riouxi*. Available from: https://services3.arcgis.com/W1gaXmEpGR8h1K59/arcgis/rest/services/maghreb/FeatureServer

**Fig 21:**
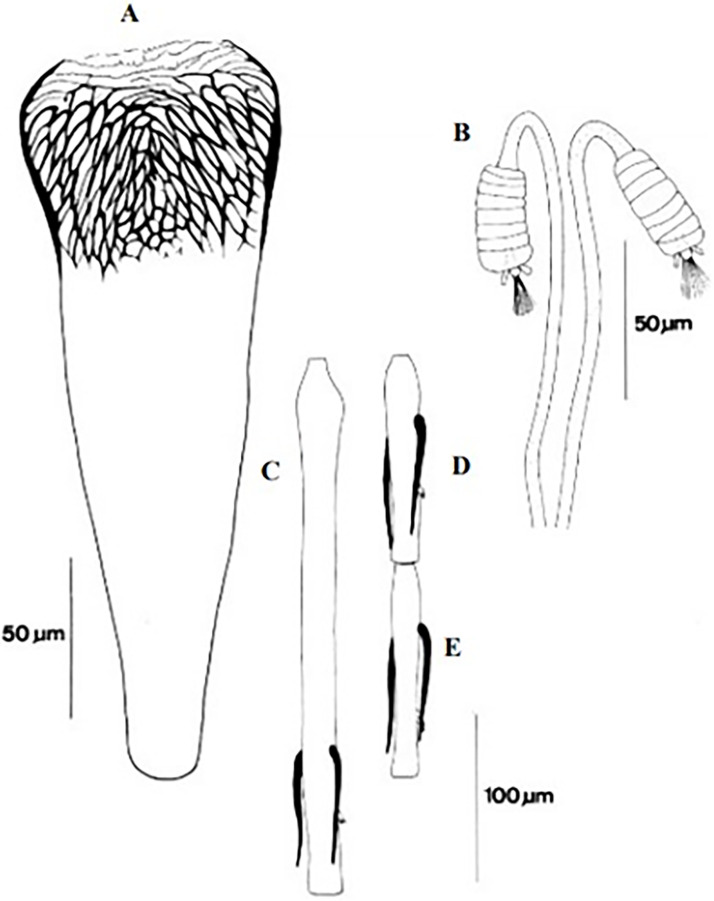
*Phlebotomus riouxi* ♀ [51]. **(A)** Pharynx. **(B)** Spermathecae. **(C)** Third antenna segment. **(D)** Fourth antenna segment. **(E)** Fifth antenna segment.

**Fig 22:**
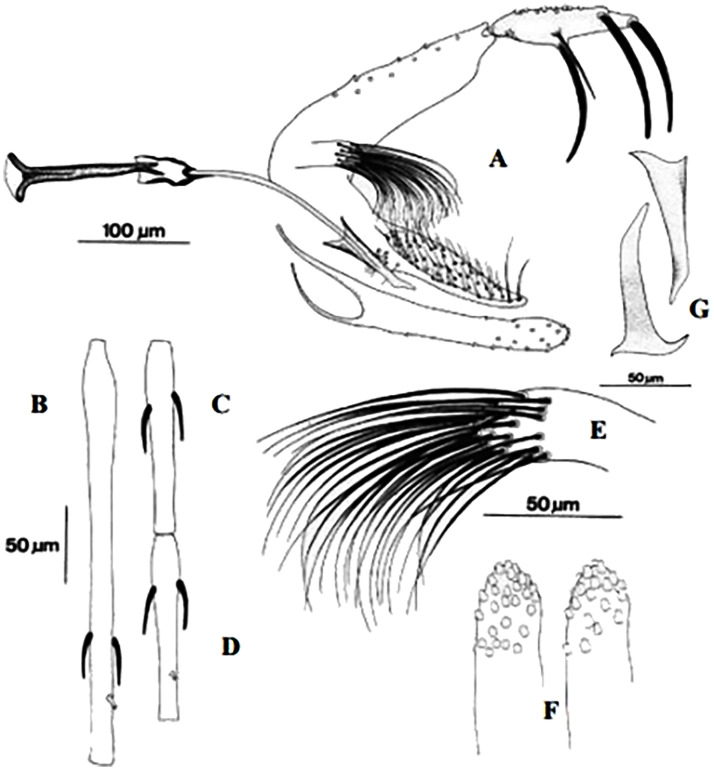
*Phlebotomus riouxi* ♂ [51]. **(A)** General genitalia. **(B–D)** Third, fourth, and fifth antenna segments. **(E)** Basal lobe of the coxite. **(F)** Internal face of the basal lobe of the coxite. **(G)** Aedeagus shape.

Until now, no role in the transmission of any pathogen has been reported for this species. Its known distribution in the Maghreb region is very limited, reported only in Algeria and Tunisia in the arid and hyper arid bioclimatic zones (Fig 20). It is closely related to *Ph*. *chabaudi*: Females are morphologically distinguished only by the absence of lateral pharyngeal teeth (Fig 21), which are present in *Ph*. *chabaudi* [15], and males differ by the higher number of setae at the basal coxite (Fig 22).

### I-3 Subgenus *Larroussius* Nitzulescu 1931

This subgenus includes 7 species present in Maghreb, all either proven or suspected to transmit *Leishmania infantum*. Males of this subgenus have 5 long spines on the style (3 terminal spines and 2 at the distal position), long aedeagus with species-specific tip shape and simple paramere. In females, pharyngeal armature consists of typical punctiform teeth and segmented spermathecae bear finger-like necked head with a basal structure [59].

### I-3-1 *Phlebotomus perniciosus* Newstead, 1911

**Fig 23:**
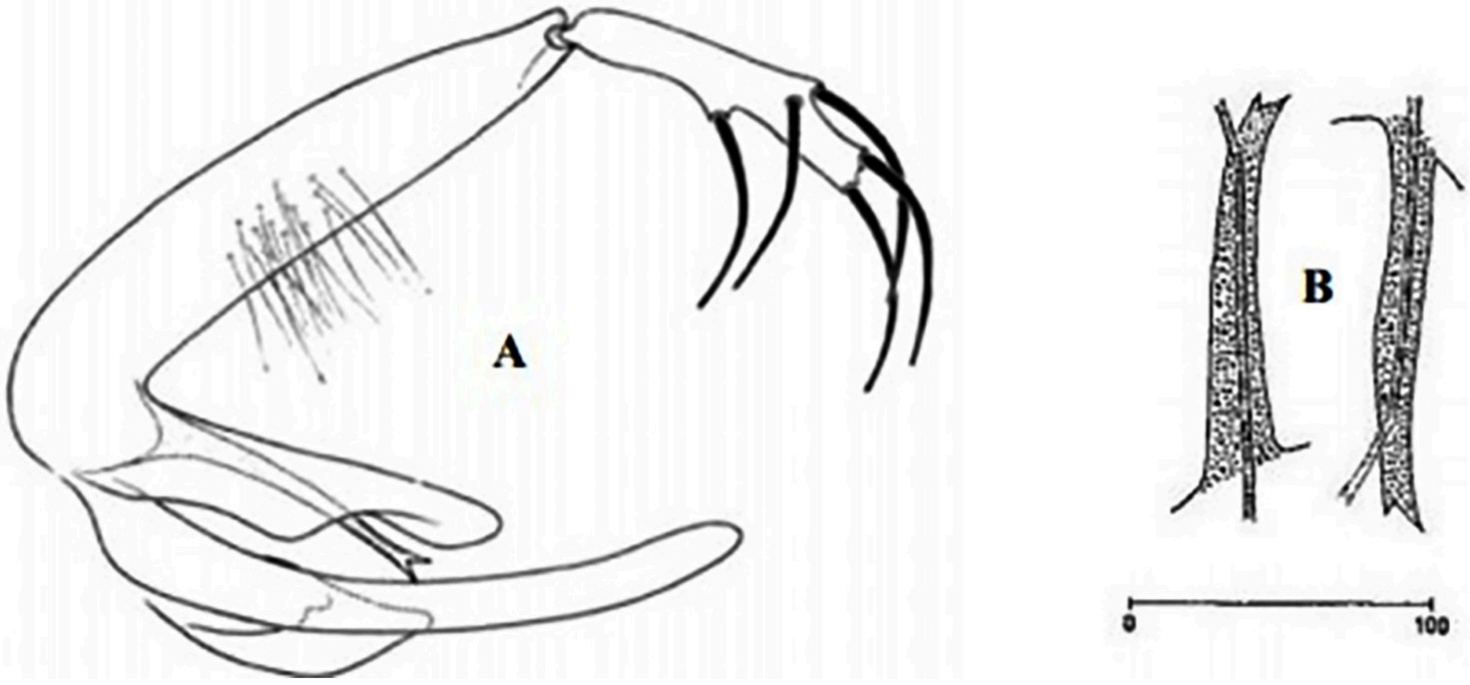
*Phlebotomus perniciosus* ♂ [36,57]. **(A)** General genitalia. **(B)** Typical aedeagus.

**Fig 24:**
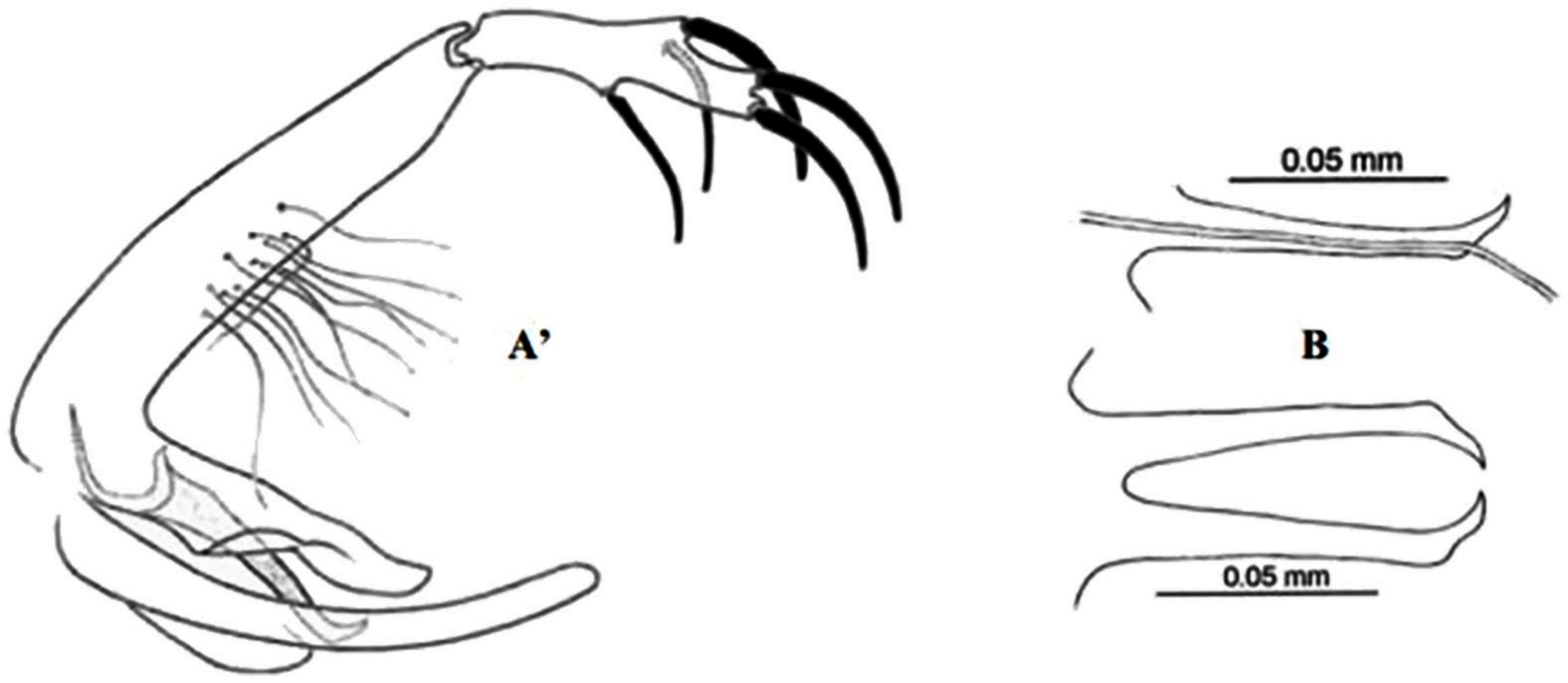
*Phlebotomus perniciosus* atypical form ♂ [44,57]. **(A’)** General genitalia. **(B)** Aedeagus shape.

**Fig 25:**
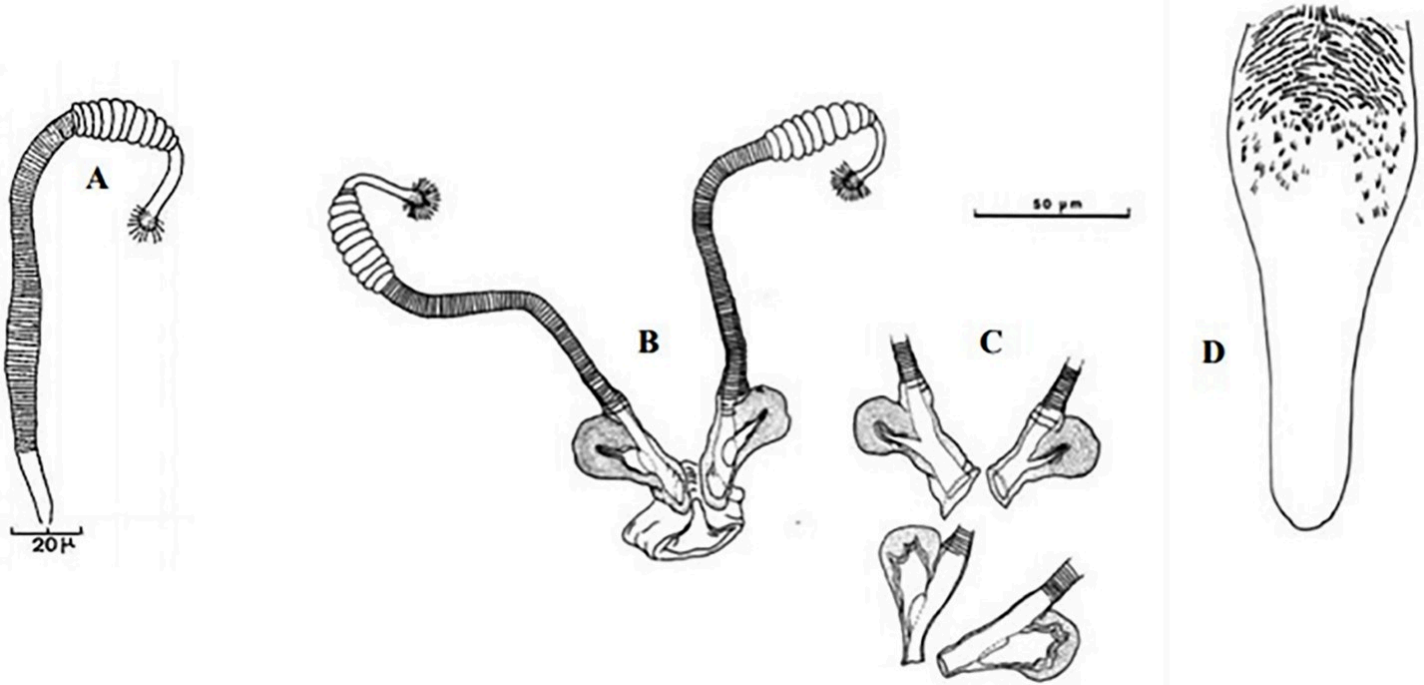
*Phlebotomus perniciosus* ♀ [27,52]. **(A)** Atypical spermathecae. **(B)** Typical spermathecae. **(C)** Reservoir at the subterminal part of the duct. **(D)** Pharynx.

**Fig 26:**
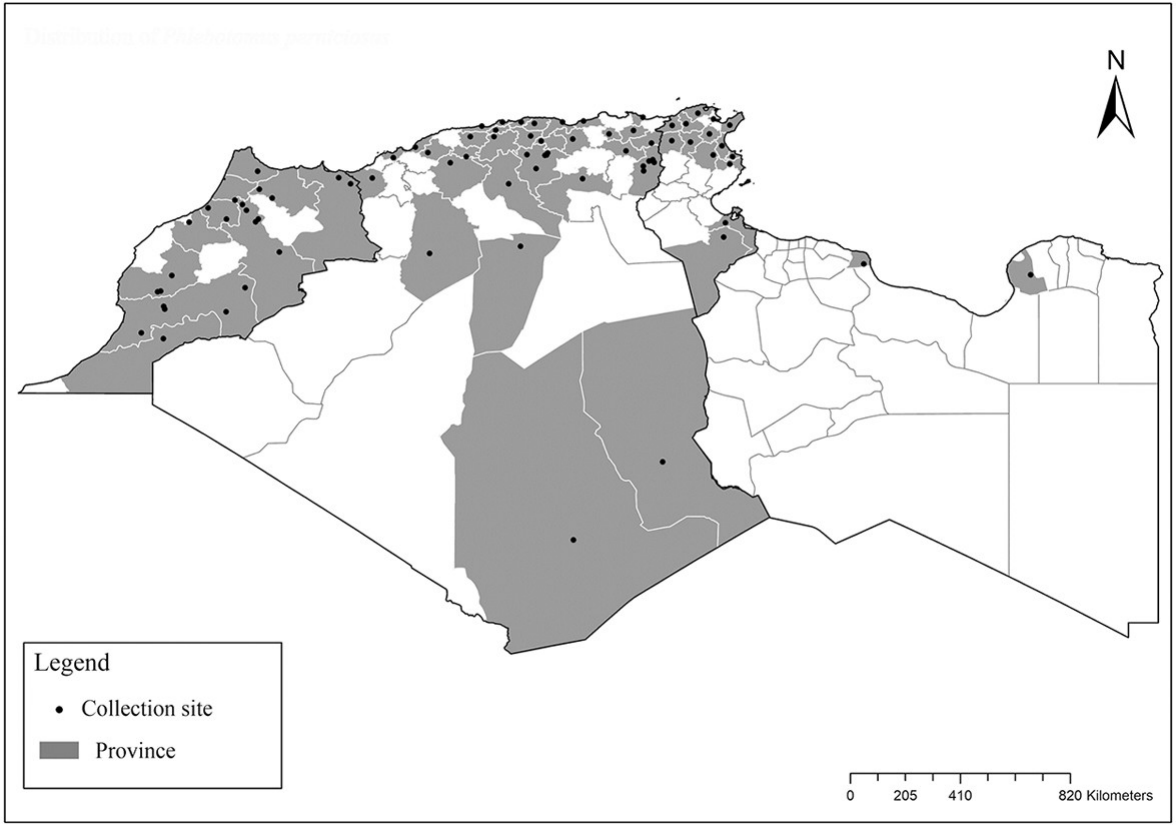
Distribution of *Phlebotomus perniciosus*. Available from: https://services3.arcgis.com/W1gaXmEpGR8h1K59/arcgis/rest/services/maghreb/FeatureServer.

**Fig 27:**
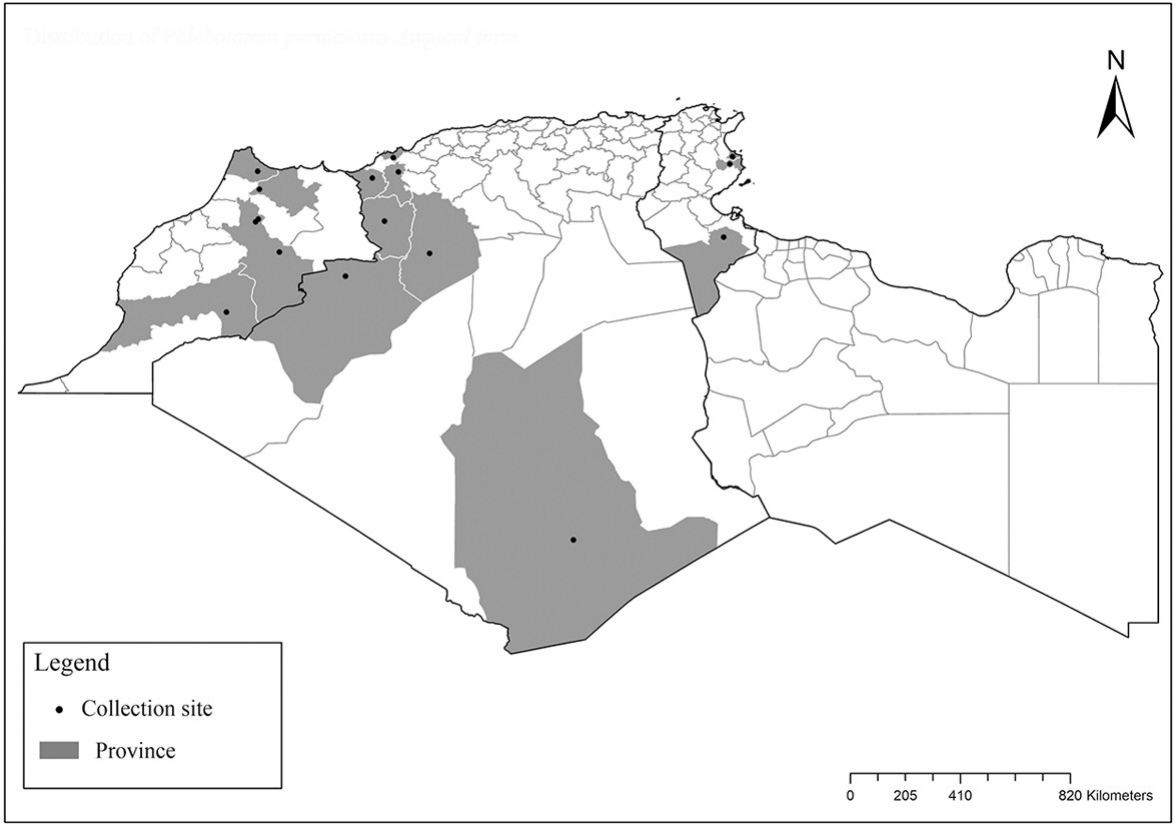
Distribution of typical *Phlebotomus perniciosus*. Available from: https://services3.arcgis.com/W1gaXmEpGR8h1K59/arcgis/rest/services/maghreb/FeatureServer.

This species is significant in the epidemiology of VL as well as canine leishmaniasis (CanL), being the major vector of *L*. *infantum* in the western part of the Mediterranean basin [5,21,81,82]. TOSV and Punique virus were isolated from this species in Tunisia [82]. Aedeagus has typically bifurcated at its tip (Fig 23). However, an atypical form of males (for long time confused with *Ph*. *longicuspis*) was recently reported in Morocco, Algeria, and Tunisia; its aedeagus was not typically bifurcated but rather curved at the tip (Fig 24) [30,83,84]. The females are recognized by a necked spermathecae, each spermiduct has a lateral and relatively thick-walled bulb at the base (Fig 25B). In some females, collected in Morocco and Libya, no bulb structures were observed (Fig 25A), which let to speculations that they may belong to the atypical form [24,41], but this was not confirmed yet. This species occurs mainly from the humid to arid bioclimatic zone but can also be found in the Saharan bioclimatic zones in the south area and central Sahara (Figs 26 and 27), albeit with low density compared to the northern regions [30,85].

### I-3-2 *Phlebotomus longicuspis* Nitzulescu, 1930

**Fig 28:**
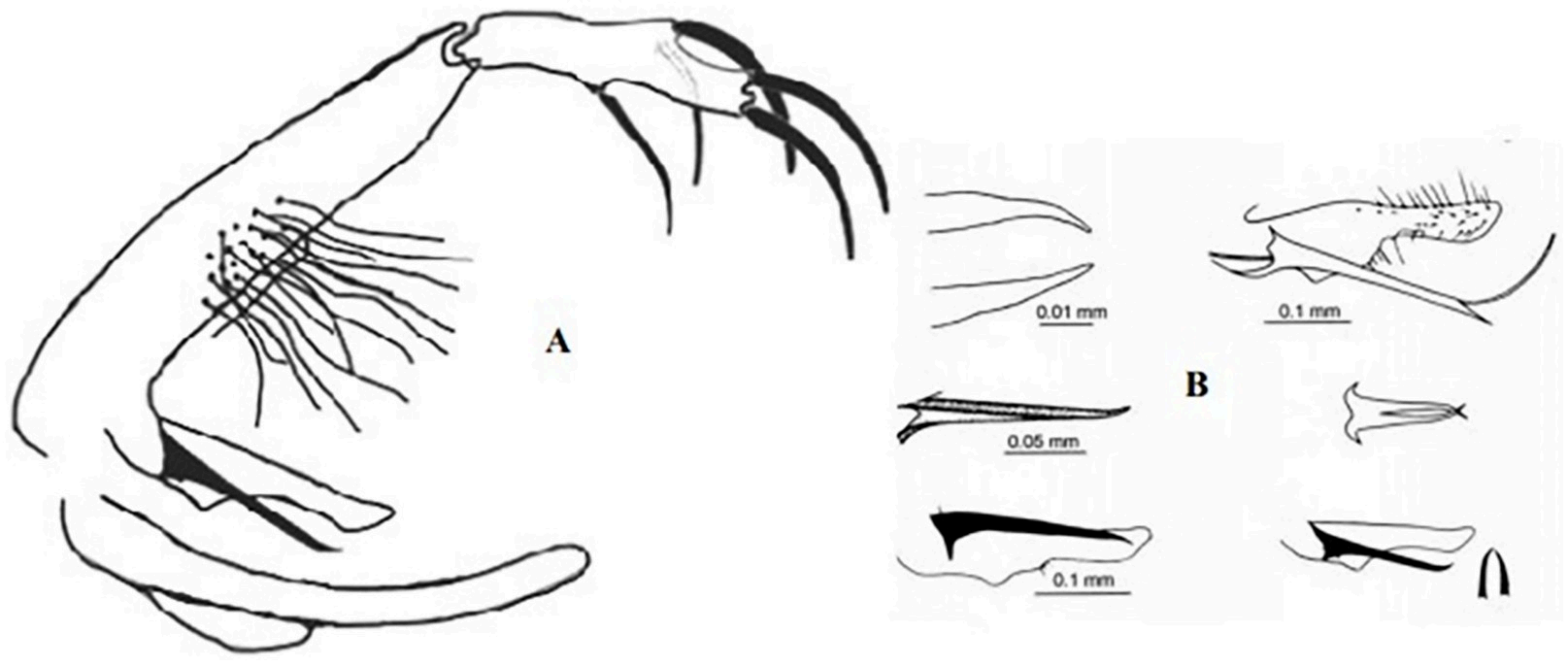
*Phlebotomus longicuspis* ♂ [44,57]. **(A)** General genitalia. **(B)** Aedeagus shapes.

**Fig 29:**
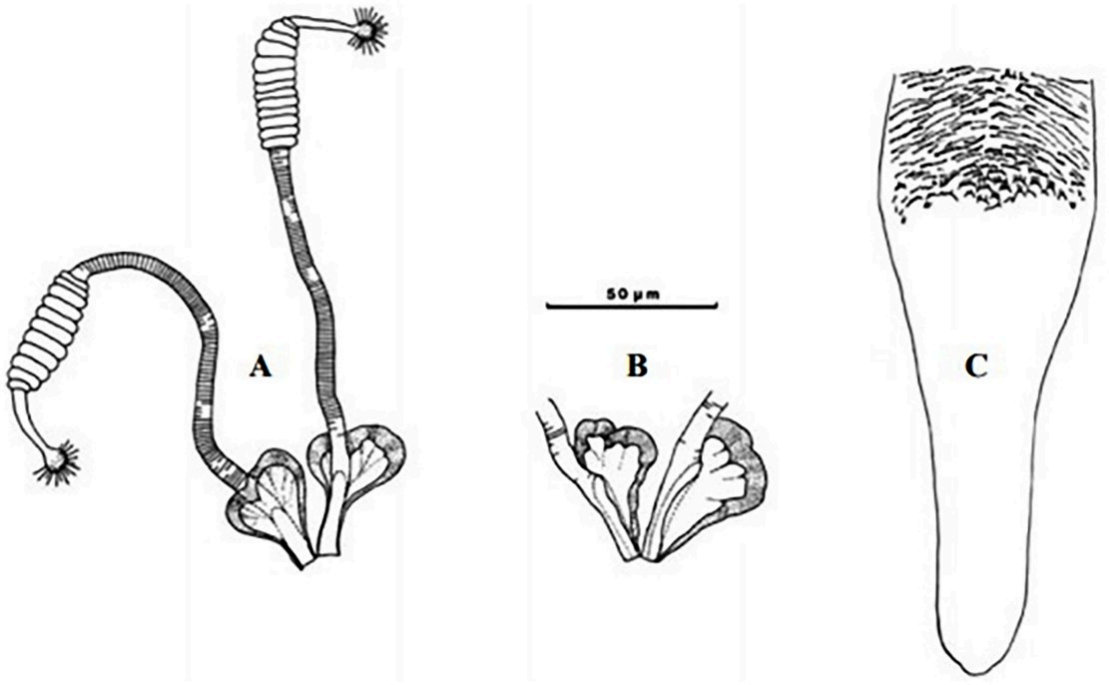
*Phlebotomus longicuspis* ♀ [52]. **(A)** Spermathecae. **(B)** Reservoir at the subterminal part of the duct. **(C)** Pharynx.

**Fig 30:**
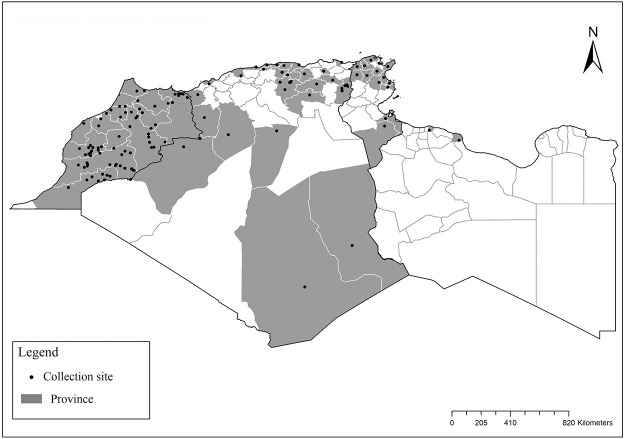
Distribution of *Phlebotomus longicuspis*. Available from: https://services3.arcgis.com/W1gaXmEpGR8h1K59/arcgis/rest/services/maghreb/FeatureServer.

Reported to be naturally infected by unidentified *Leishmania* parasites [33] and recently shown to be positive for *L*. *infantum* DNA by PCR screening [86], it still is a suspected vector of VL in Algeria, Morocco, and Tunisia [67]. It has been found infected also by Naples virus in Algeria [87], by Punique virus in Tunisia [82], and by TOSV in Morocco [73]. The males are recognized by a long aedeagus pointed at the tip (Fig 28) whereas the females by long-necked spermathecae similar to *Ph*. *perniciosus* but having relatively thin-walled bulb at the base of spermiducts (Fig 29). This species is generally recorded in sympatry with *Ph*. *perniciosus* from the subhumid to Sahara bioclimatic zones (Fig 30) [30].

### I-3-3 *Phlebotomus perfiliewi* Parrot, 1930

**Fig 31:**
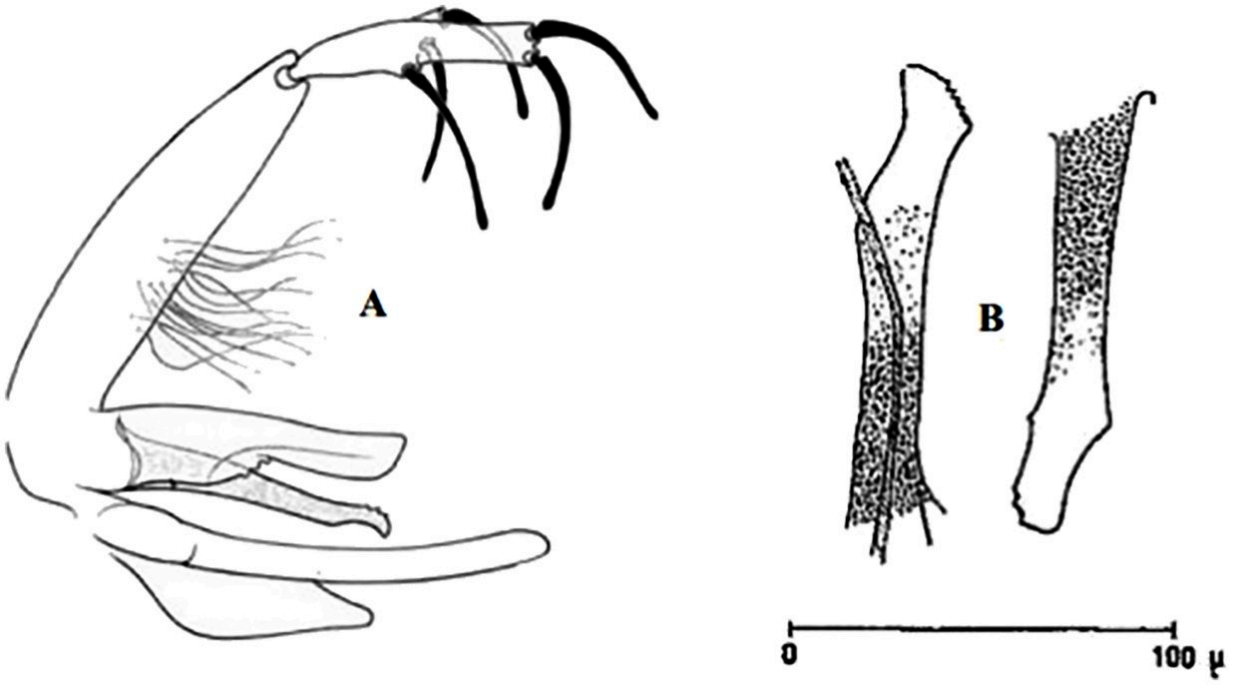
*Phlebotomus perfiliewi* ♂ [36,57]. **(A)** General genitalia. **(B)** Aedeagus shape.

**Fig 32:**
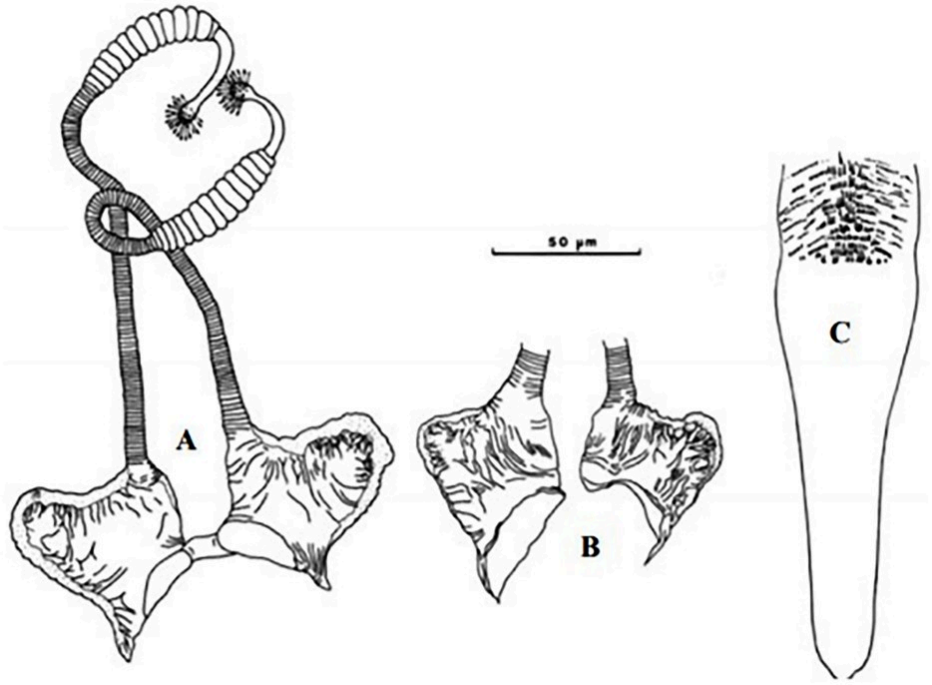
*Phlebotomus perfiliewi* ♀ [52]. **(A)** Spermathecae. **(B)** Reservoir at the subterminal part of the duct. **(C)** Pharynx.

**Fig 33:**
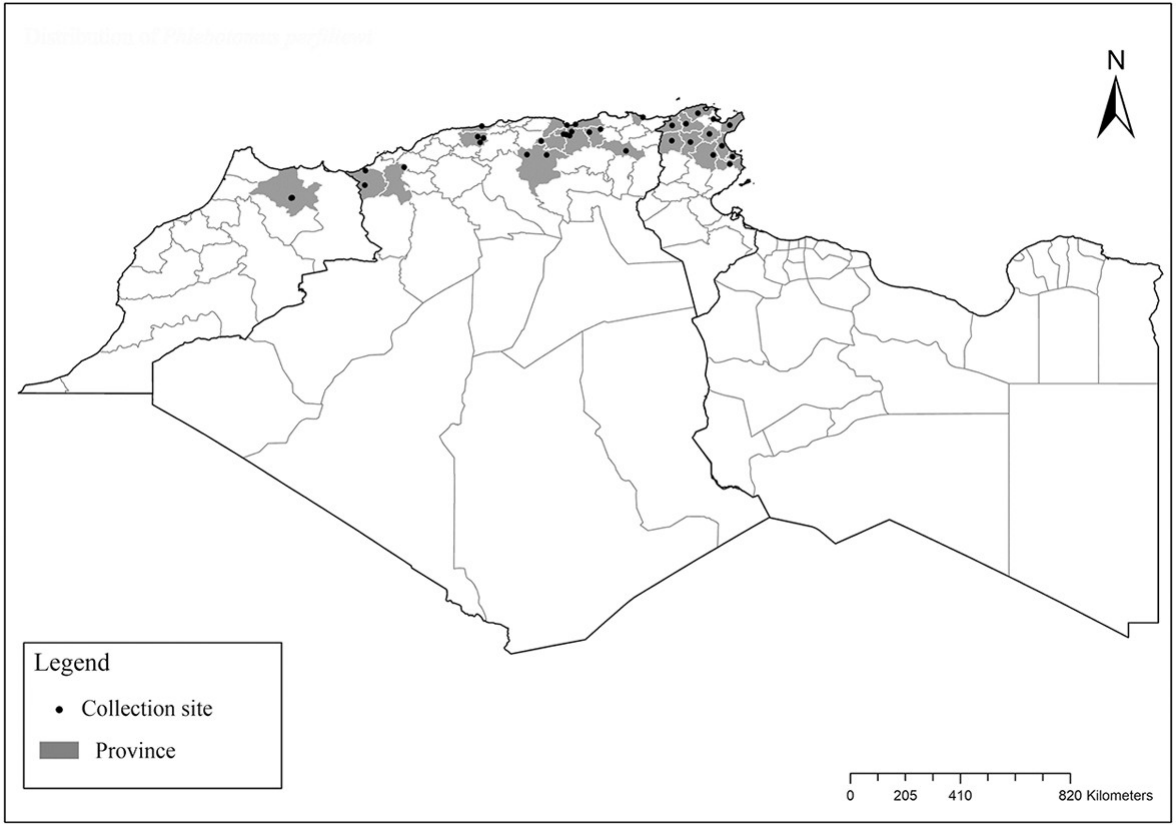
Distribution of *Phlebotomus perfiliewi*. Available from: https://services3.arcgis.com/W1gaXmEpGR8h1K59/arcgis/rest/services/maghreb/FeatureServer.

It is a proven vector of *L*. *infantum* in Mediterranean basin. In Algeria, this species transmits *L*. *infantum* Mon-24, which causes sporadic CL [88] and TOSV [89]. Depaquit and colleagues [90] showed that this species forms a complex of species that includes 3 taxa: *Ph. perfiliewi* (Parrot, 1930) *s*. *s*., *Ph. galilaeus* (Theodor, 1958), and *Ph. transcaucasicus* (Perfiliev, 1937). Males (Fig 31) of these taxa differ in shape of aedeagus, but females are indistinguishable by morphology, all having large lateral and triangular pocket near the opening of each spermiducts (Fig 32). In the Maghreb region, only *Ph*. *perfiliewi s*. *s*. is present, occurring in Morocco, Algeria, and Tunisia [33,36,90,91]. It is found in subhumid, arid, and subarid regions at the latitudes up to 1,200 meters above the sea [33]. Until now, it was not reported in Libya (Fig 33).

### I-3-4 *Phlebotomus ariasi* Tonnoir, 1921

**Fig 34:**
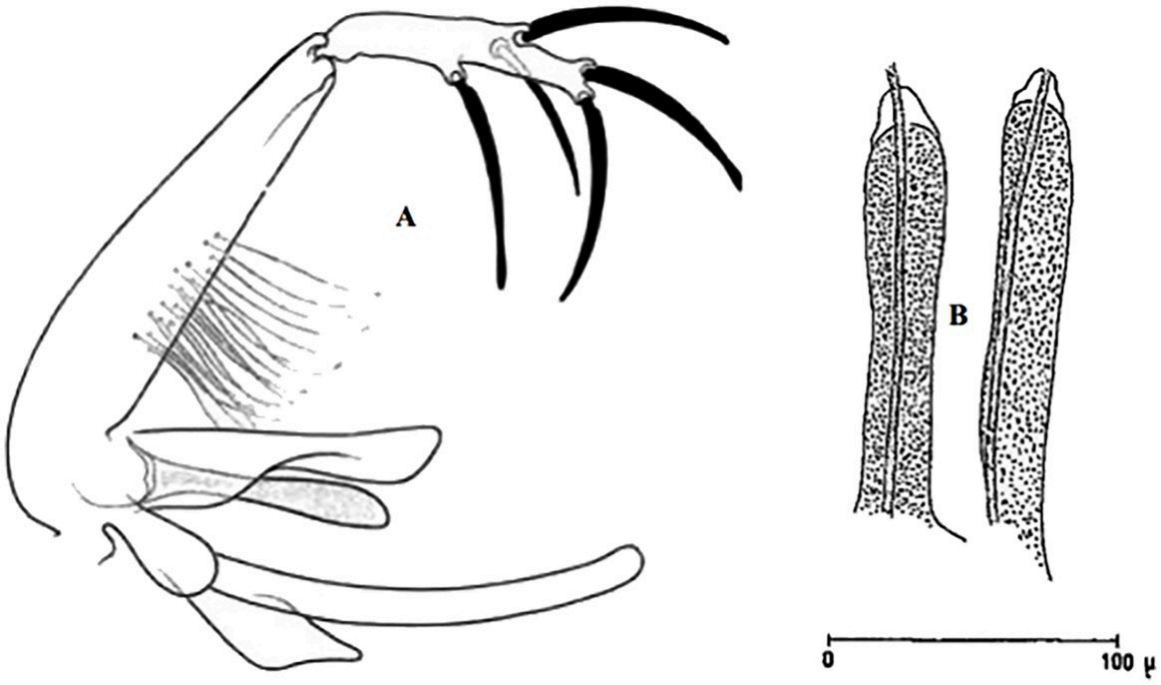
*Phlebotomus ariasi* ♂ [36,57]. **(A)** General genitalia. **(B)** Aedeagus shape.

**Fig 35:**
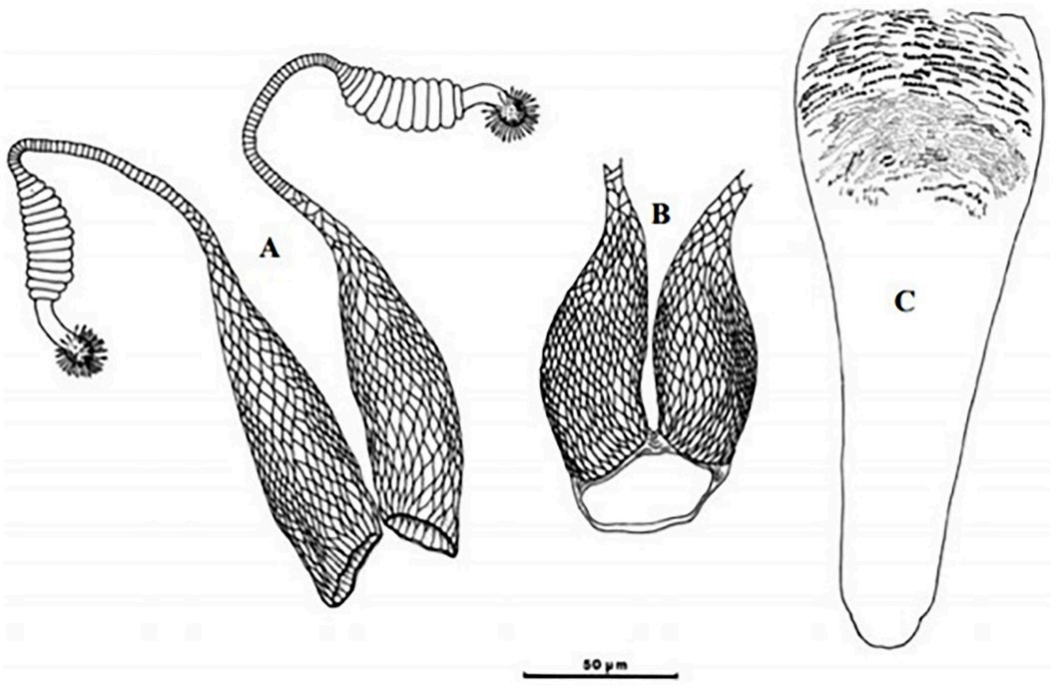
*Phlebotomus ariasi* ♀ [52]. **(A)** Spermathecae. **(B)** Reservoir at the subterminal part of the duct. **(C)** Pharynx.

**Fig 36:**
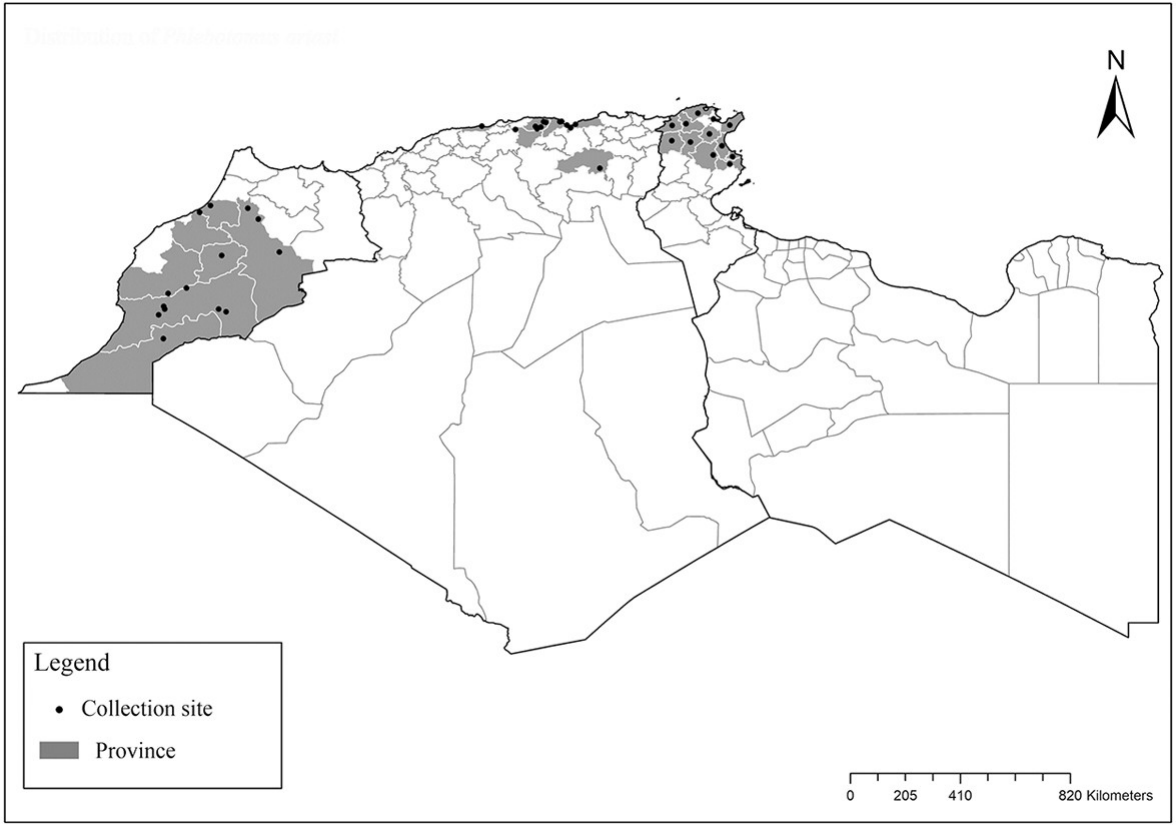
Distribution of *Phlebotomus ariasi*. Available from: https://services3.arcgis.com/W1gaXmEpGR8h1K59/arcgis/rest/services/maghreb/FeatureServer.

It is considered as a proven vector of *L*. *infantum* in western part of the European Mediterranean countries, and, rarely, it transmits the located and diffused CL [67]. Until now, a single female was found naturally infected by *L*. *infantum* in Morocco [92]. A recent study reported its infection by Sicilian virus in Algeria [93]. Aedeagus is relatively thick with rounded tip (Fig 34). In females, distal half of the spermiducts is swollen (Fig 35). It occurs from the humid to arid bioclimatic zones, mainly in the olive, Ilex oaks, and cedar vegetation parts. In Algeria (Great Kabylia), it was reported at 4,400 meters above sea level [27,33,36]. This species has not been reported in Libya yet (Fig 36).

### I-3-5 *Phlebotomus chadlii*, Rioux, Juminer et Gibily, 1966

**Fig 37:**
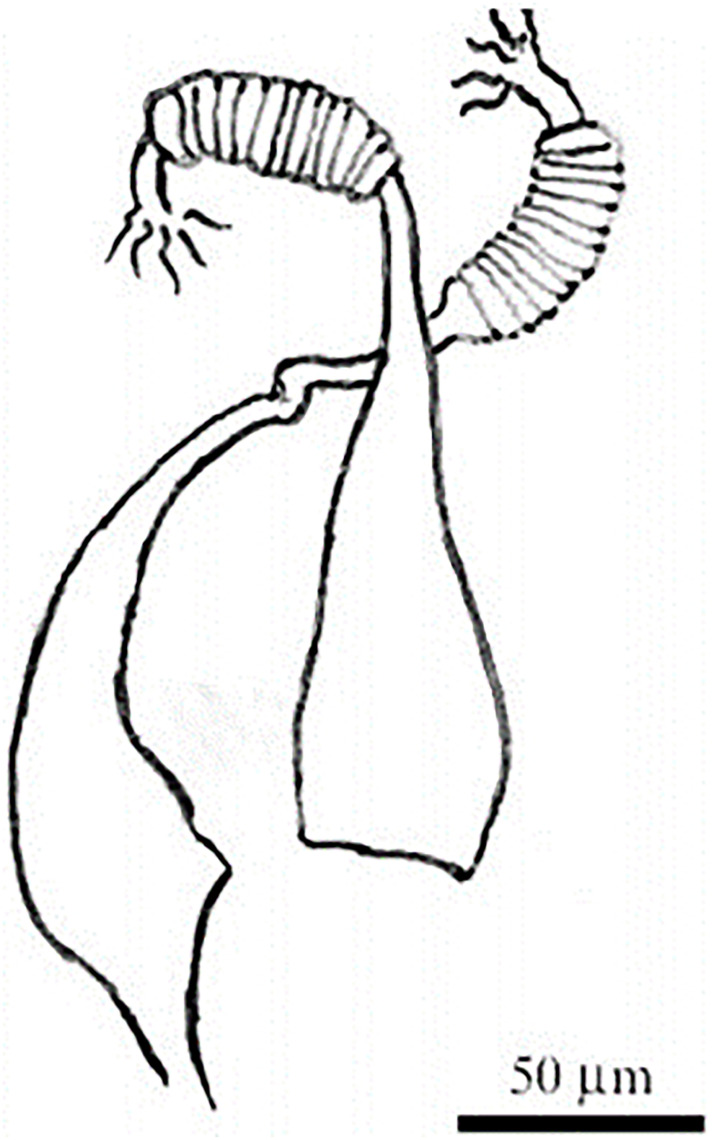
*Phlebotomus chadlii* ♀ [60]. Spermathecae and the reservoir at the subterminal part of the duct.

**Fig 38:**
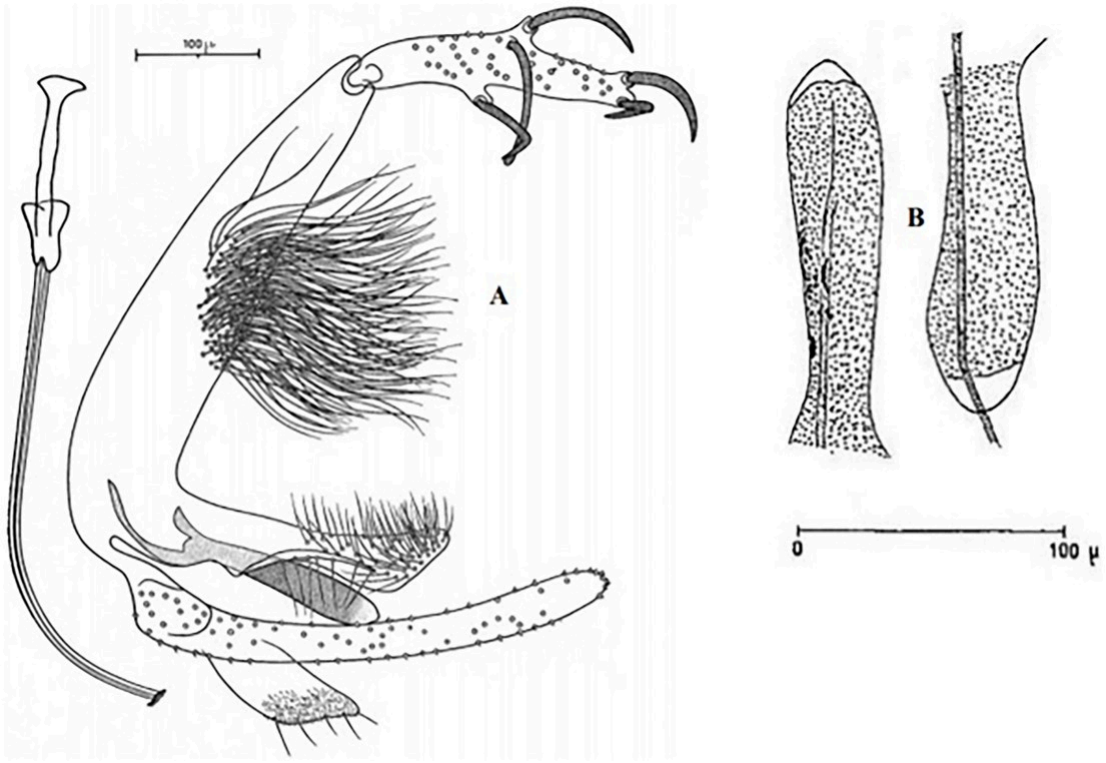
*Phlebotomus chadlii* ♂ [36,49]. **(A)** General genitalia. **(B)** Aedeagus shape.

**Fig 39:**
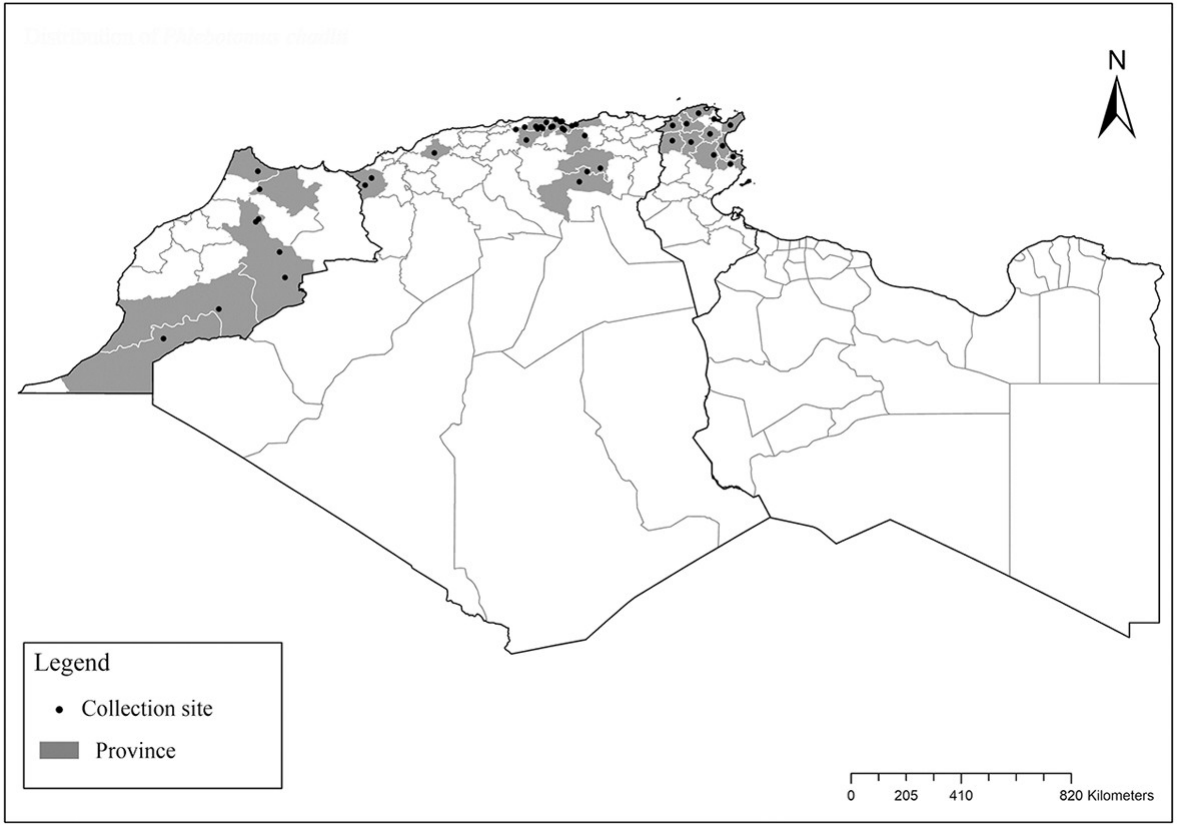
Distribution of *Phlebotomus chadlii*. Available from: https://services3.arcgis.com/W1gaXmEpGR8h1K59/arcgis/rest/services/maghreb/FeatureServer.

There is no information about its involvement in *Leishmania* transmission cycles and due to its rare occurrence, it is likely not to be of epidemiological significance. Its description is based on a male from southern Tunisia in 1965 [94]; later, it was reported in Algeria in 1970 [80] then in Morocco in 1975 [95]. The first female was described in Tunisia in 2006 [57] and in 2011 in Algeria (Fig 37) [32]. So far, this species has been not recorded in Libya. Morphologically it is close to *Ph. ariasi*, but males have shorter aedeagus and higher number of coxite hairs (Fig 38). It occurs between the humid and hyperarid bioclimatic zones (Fig 39).

### I-3-6 *Phlebotomus langeroni*, Nitzulescu, 1930

**Fig 40:**
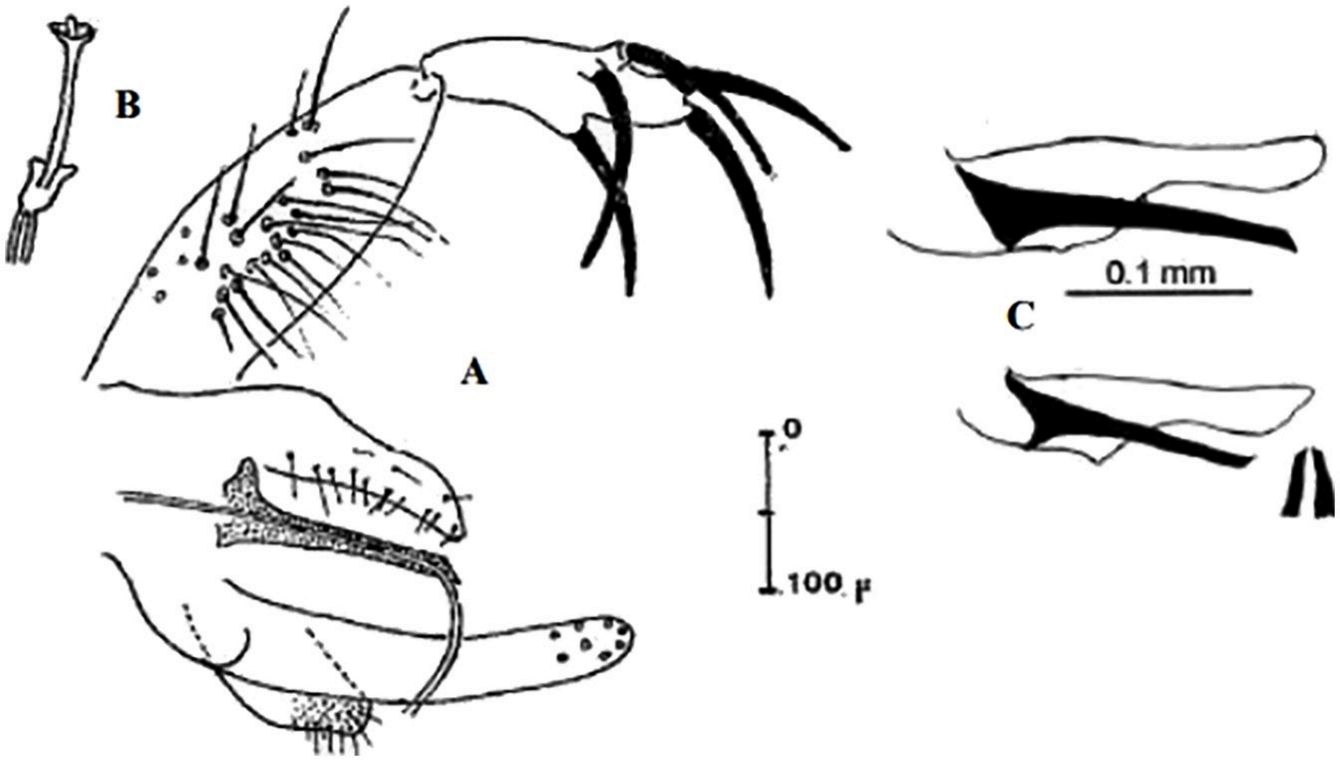
*Phlebotomus langeroni ♂* [36,44]. **(A)** General genitalia. **(B)** Genitalia pump. **(C)** Different reported aedeagus.

**Fig 41:**
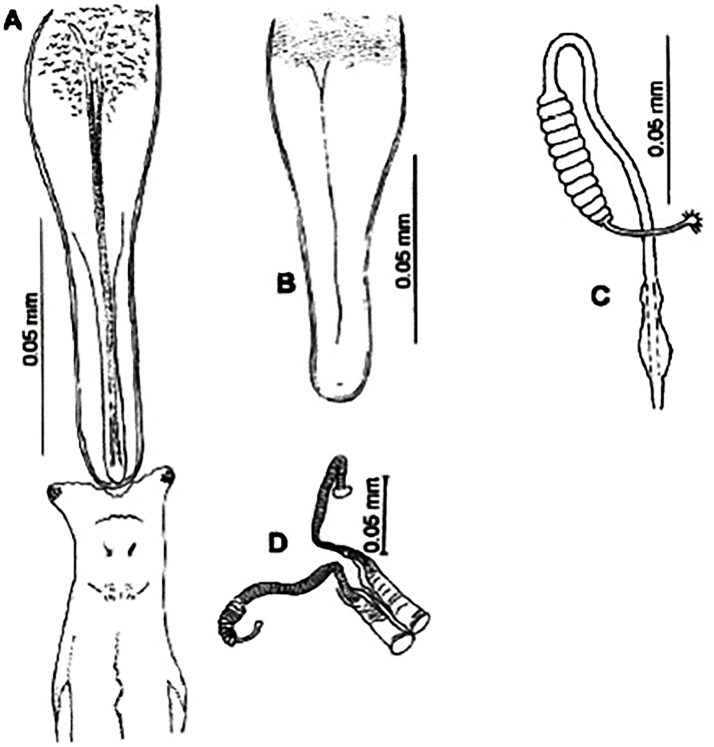
*Phlebotomus langeroni* ♀ [44]. **(A, B)** Pharynx. **(C, D)** Spermathecea and the reservoir at the subterminal part of the duct.

**Fig 42:**
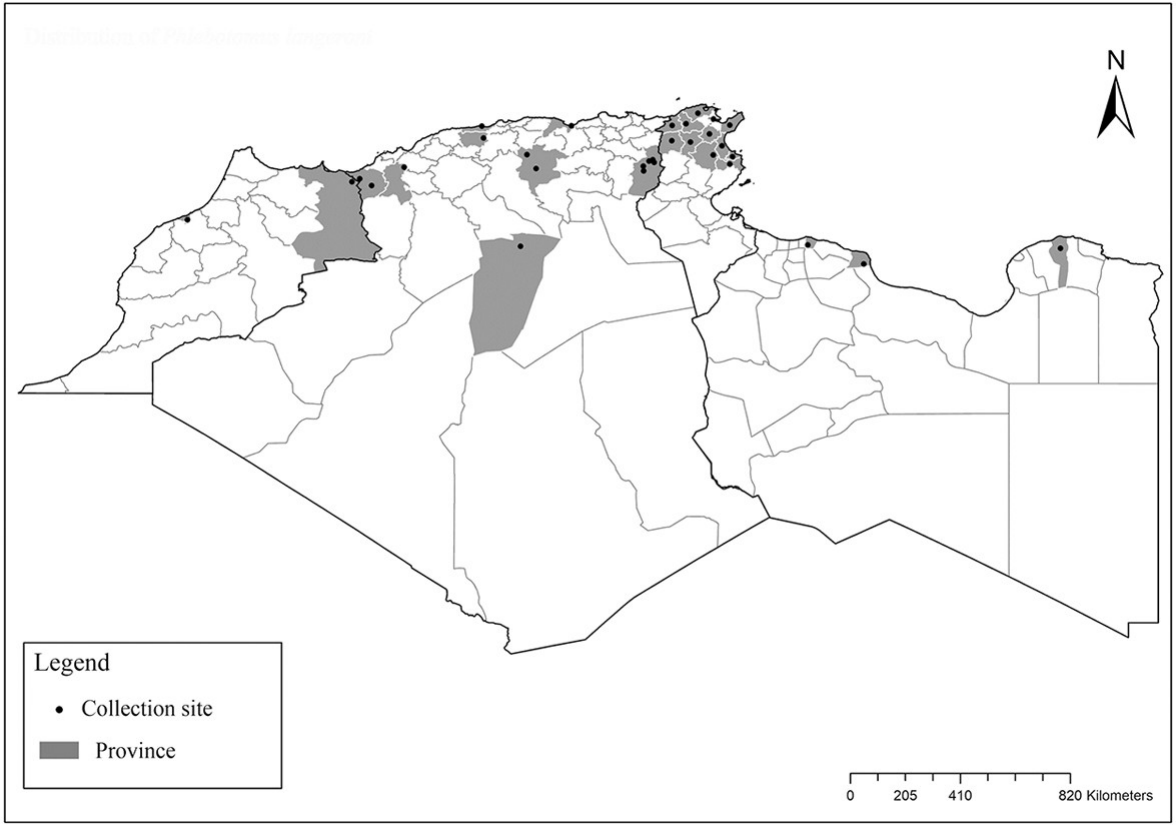
Distribution of *Phlebotomus langeroni*. Available from: https://services3.arcgis.com/W1gaXmEpGR8h1K59/arcgis/rest/services/maghreb/FeatureServer.

This species was found naturally infected by *L*. *infantum* in Tunisia [96,97] and shown to transmit *L*. *infantum* MON-98 under laboratory conditions [98]. The male is recognized by a long and bevel-shaped aedeagus at the tip (Fig 40). Female spermathecae have middle segments larger than the end segments and long slender neck with apical knob (Fig 41). Distal part of spermiducts is swollen but differs in shape from *Ph*. *ariasi*. Described first as a subspecies of *Ph*. *perniciosus*, based on males collected in Tunisia, then in 1930, Nitzulescu arose it to the species level [99]. In 2005, the first female specimen was also described in Tunisia [100]. It occurs from humid to arid bioclimatic zones (Fig 42).

### I-3-7 *Phlebotomus mariae*, Rioux, Croset, Léger et Bailly-Choumara, 1974

**Fig 43:**
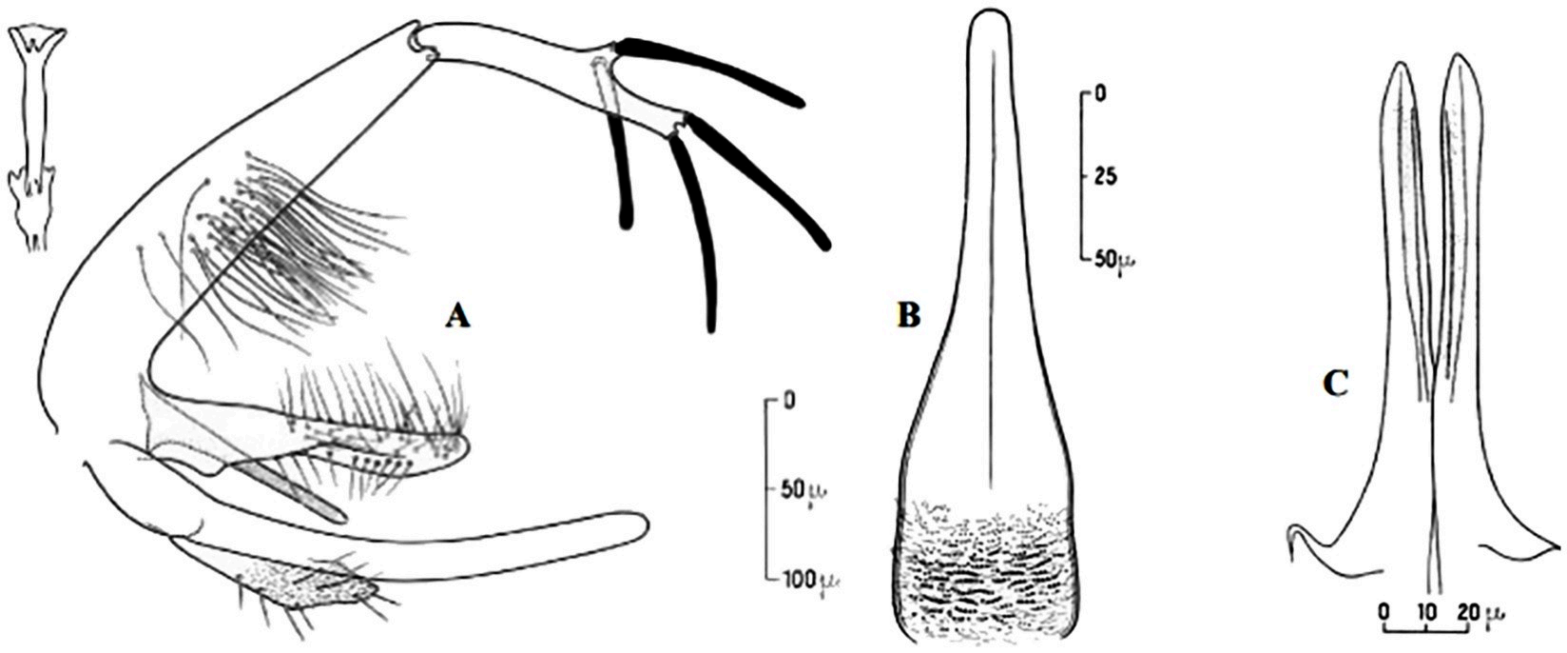
*Phlebotomus mariae* ♂ [28]. **(A)** General genitalia. **(B)** Pharynx. **(C)** Aedeagus.

**Fig 44:**
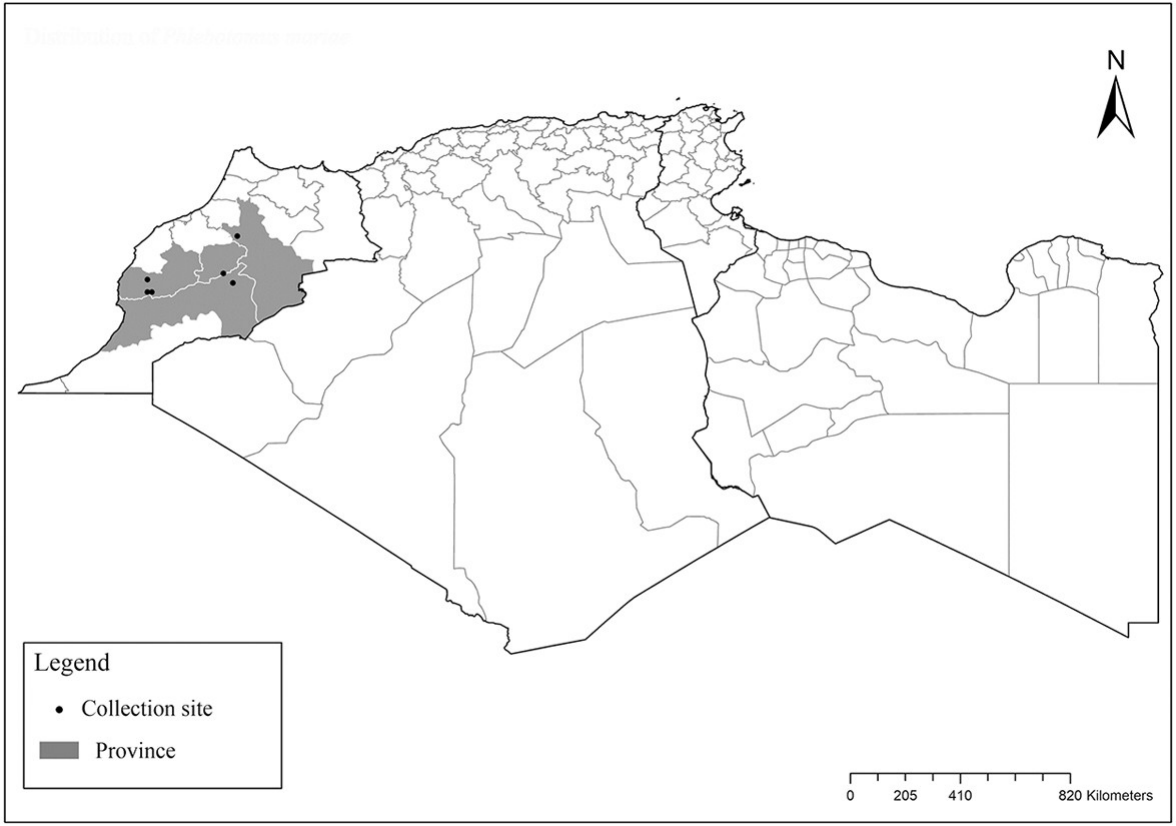
Distribution of *Phlebotomus mariae*. Available from: https://services3.arcgis.com/W1gaXmEpGR8h1K59/arcgis/rest/services/maghreb/FeatureServer.

One of the most elusive *Larroussius* species, described based only on males and considered to be closely related with *Ph*. *ariasi* [101]. Aedeagus are long, narrow with subparallel edges and very discreetly swelling at the end tip (Fig 43). So far, it was recorded only in few localities of Morocco from subhumid to subarid bioclimatic zones (Fig 44). Females of this species remain unknown [25,101,102].

### I-4 Subgenus *Transphlebotomus* Artemiev 1984

The subgenus *Transphlebotomus* was established by Artemiev in 1984 based on the male genital morphology and female characters for spermathecae and pharyngeal armature [103]. The species belonging to this genus are defined by individual spermathecal ducts being broad, joining at genital opening; spermathecae are simple striated tubes, not clearly demarcated into segments, with a small head and pharyngeal teeth point forward [59]. Of 5 species so far described in the Mediterranean region, some of these notably recently [104,105], only 1 has been recorded in Maghreb region. Due to minor morphological differences between species, morphological identifications of *Transphlebotomus* should be confirmed by molecular methods.

### I-4-1 *Phlebotomus mascittii*, Grassi, 1908

**Fig 45:**
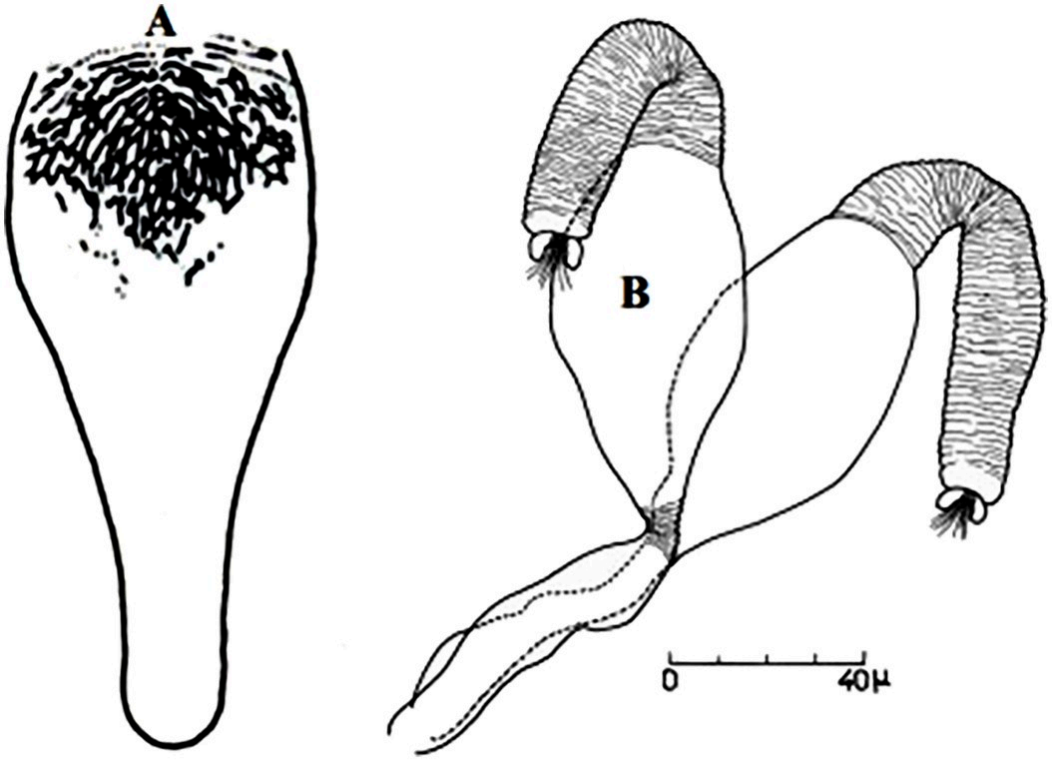
*Phlebotomus mascitti* ♀ [53]. A) Pharynx, B) Spermathecae.

**Fig 46:**
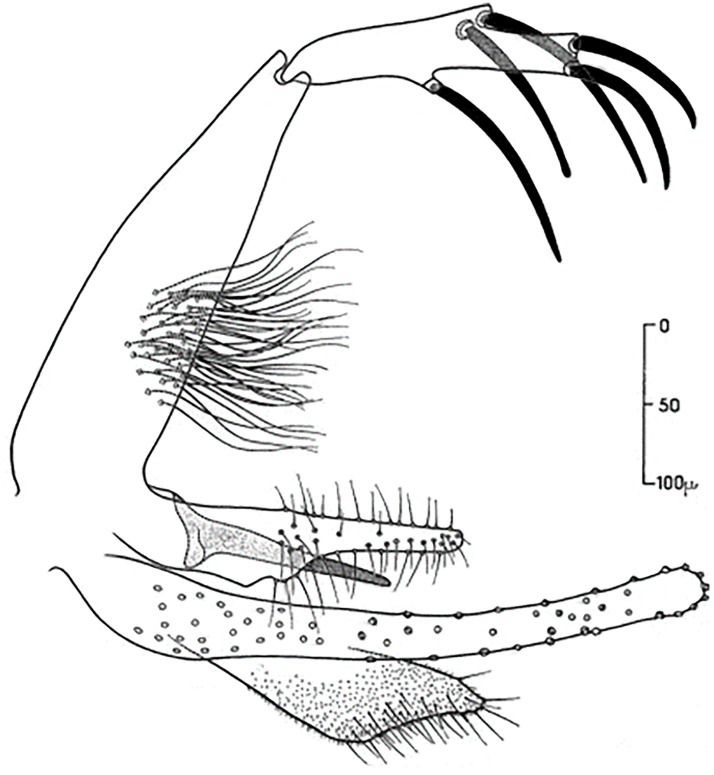
*Phlebotomus mascitti* ♂ [50]. General genitalia.

**Fig 47:**
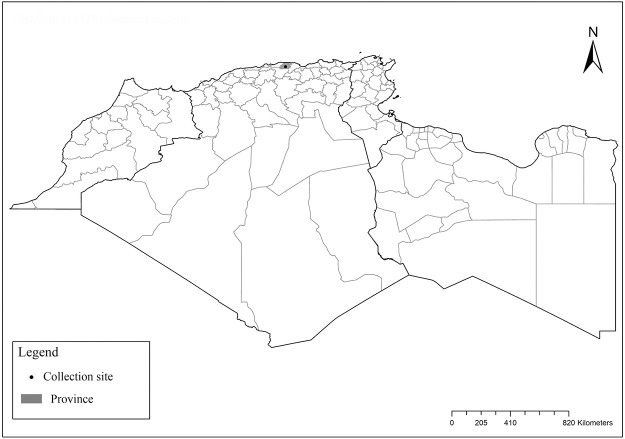
Distribution of *Phlebotomus mascittii*. Available from: https://services3.arcgis.com/W1gaXmEpGR8h1K59/arcgis/rest/services/maghreb/FeatureServer.

Suspected but so far not proven vector of *L*. *infantum* due to circumstantial findings of parasite DNA by PCR screening [106]. This species (Figs 45 and 46) exhibits a wide distribution in Europe that extends the usual Mediterranean regions to the north up to Austria, Germany, and Slovakia [107–109]. In the Maghreb region, its presence was recorded by a sole finding of 1 female in Tizi Ouzou, Algeria (Fig 47) in the humid bioclimatic zone [32].

### II- Genus *Sergentomyia* França and Parrot 1920

In Maghreb region, the genus *Sergentomyia* is represented by species of 4 subgenera described below (*Sergentomyia*, *Parrotomyia*, *Grassomyia*, and *Sintonius*), and the fifth subgenus *Parvidens* is present only in Mauritania. They are all defined by (i) cibarial teeth and/or denticles (more developed in females than in males and arranged in a transverse row); (ii) a pigmented patch on the dorsal wall of cibarium; and (iii) the hind end of abdomen tergites 2 to 6 bear all or most setae recumbent arising from small sockets as compared with large round sockets on first tergite. Males possess (i) a single paramere; and (ii) style with 4 terminal spines (sometime arranged as 2 terminal and 2 subterminal) and a short nondeciduous seta (accessory spine). Females spermathecae are smooth or segmented, either tubular or capsuliform [2,110]. The main differences between the species are summarized in **Tables D and E in** S1 Text for males and females, respectively.

Unlike genus *Phlebotomus*, none of the *Sergentomyia* species was conclusively proven to be a vector of *Leishmania* parasites that infect humans. However, for a number of species, including some that are also recorded in the Maghreb region, circumstantial evidence based on microscopic observations and/or PCR detection of parasite’s DNA suggest that they may be also play a role in *Leishmania* transmission. Such information is therefore mentioned for respective species listed below.

### II-1 Subgenus *Sergentomyia*

Females of this subgenus have tubular spermathecae with smooth walls of uniform width along their length, antennal segment 3 is shorter than segments 4 and 5 together and usually shorter than labrum. Males have a stout finger-like aedeagus with blunt tip [59]. So far, 5 species were reported in the Maghreb region.

### II-1-1 *Sergentomyia minuta parroti*, Adler et Theodor, 1927

**Fig 48:**
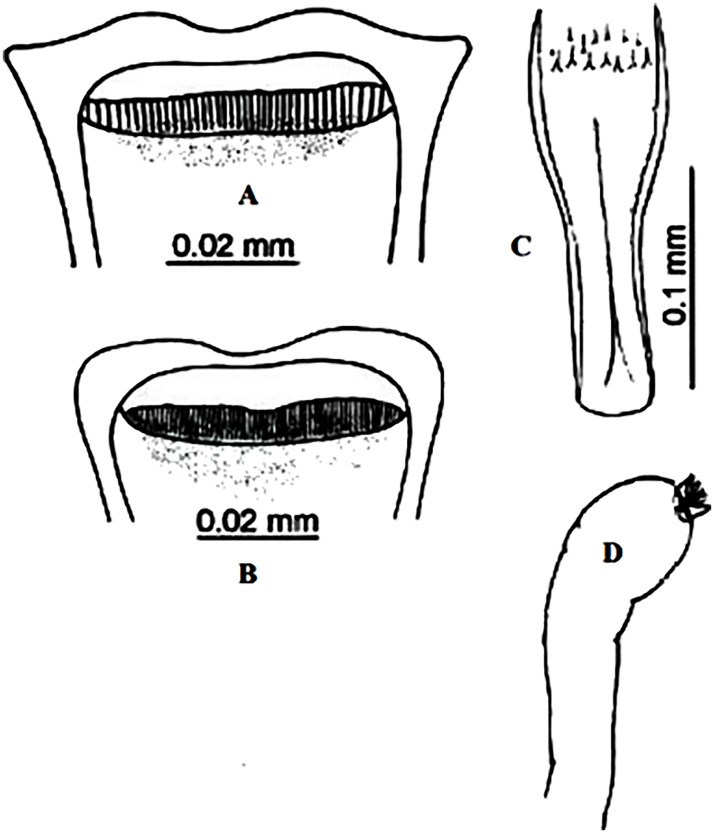
*Sergentomyia minuta* ♀ [44]. A) Cibarium of *Se*. *minuta minuta*, B) Cibarium of *Se*. *minuta parroti*, C) Pharynx, D) Spermathecae.

**Fig 49:**
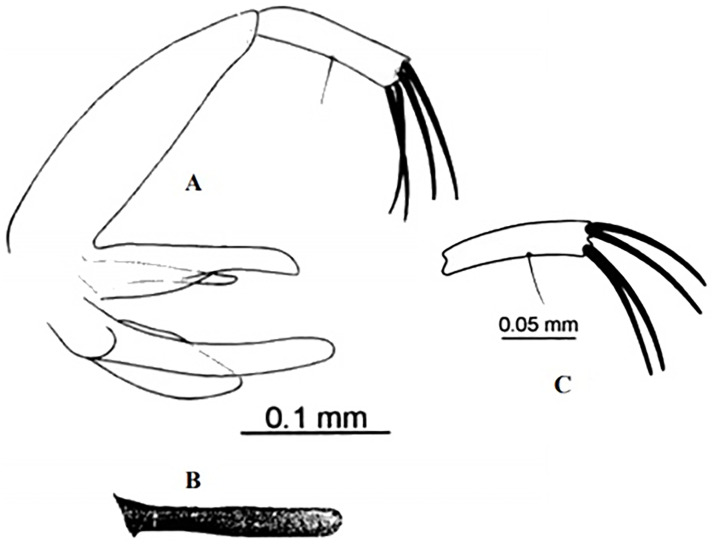
*Sergentomyia minuta* ♂ [44]. A) General genitalia, B) Aedeagus, C) Insertion position of nondeciduous seta.

**Fig 50:**
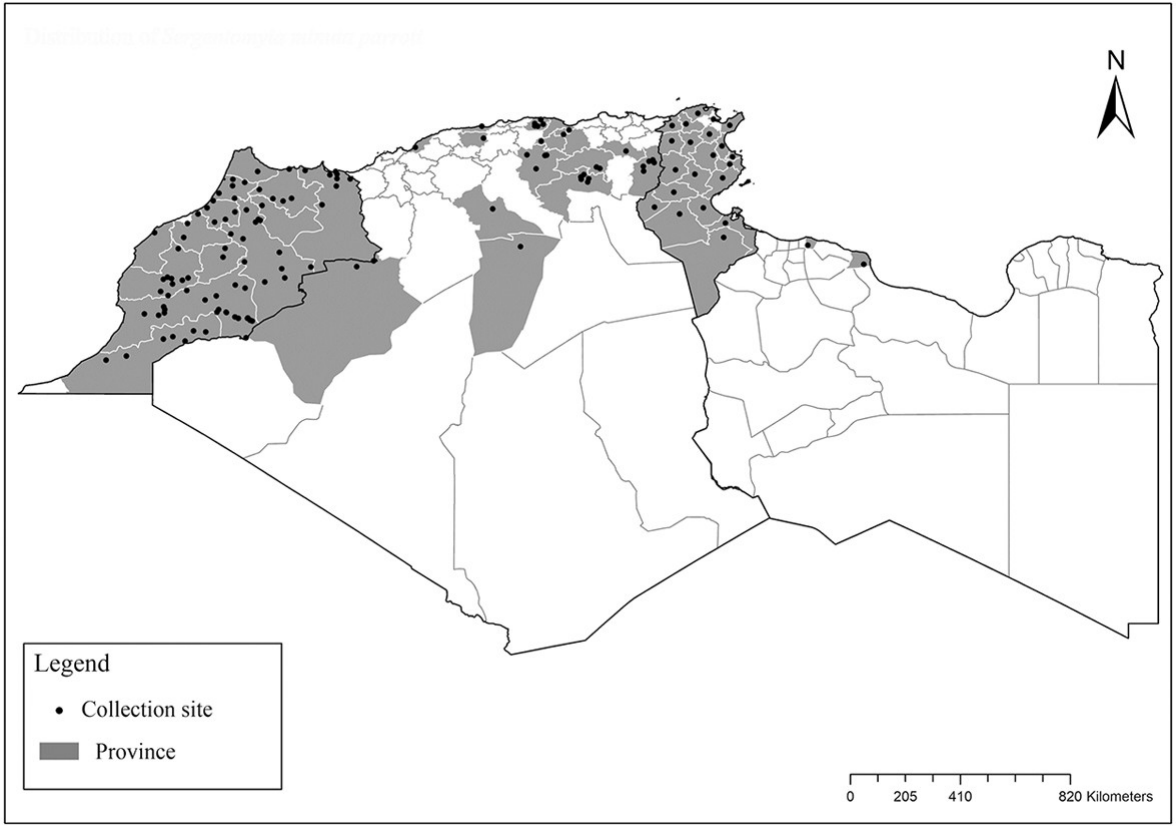
Distribution of *Sergentomyia minuta*. Available from: https://services3.arcgis.com/W1gaXmEpGR8h1K59/arcgis/rest/services/maghreb/FeatureServer.

*Sergentomyia minuta parroti* was reported to transmit *L*. *tarentolae* that infects geckoes [111,112]. Females are recognized by presence of net pigmented patch upon the straight row of cibarial teeth (Fig 48). In males, accessory spine is located in the middle of the style (Fig 49). It is an herpetophilic species found in the whole Maghreb region (Fig 50) from the humid to arid bioclimatic zones [2]. Two subspecies were described, *Se*. *minuta minuta* (Fig 48A) and *Se*. *minuta parroti* (Fig 48B), which differ in the number of cibarial teeth: around 40 for *Se*. *minuta minuta* and over 70 for *Se*. *minuta parroti* [111].

### II- 1–2 *Sergentomyia fallax*, Parrot, 1921

**Fig 51:**
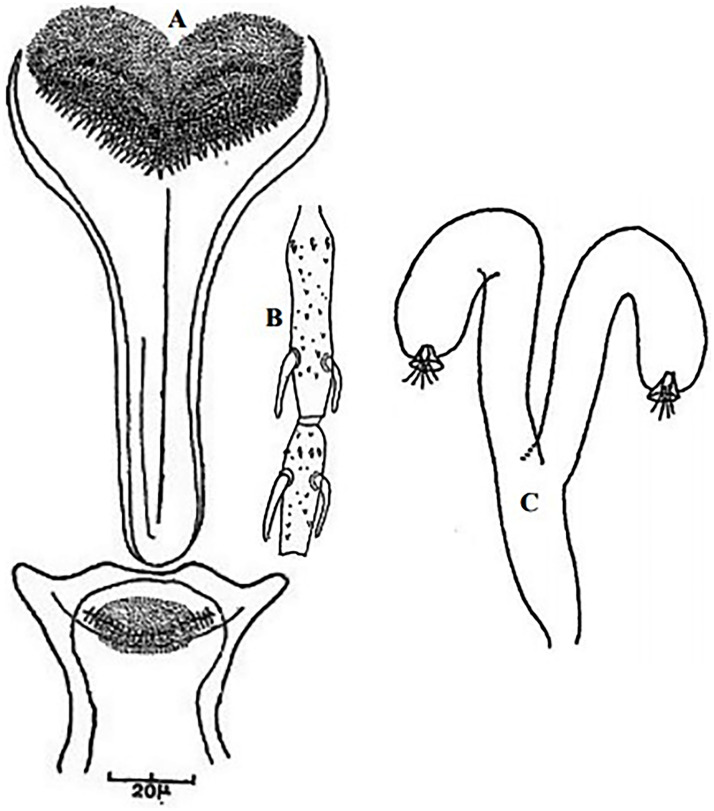
*Sergentomyia fallax* ♀ [2]. **(A)** Pharynx and Cibarium. **(B)** Third and fourth antenna segments. **(C)** Spermathecae.

**Fig 52:**
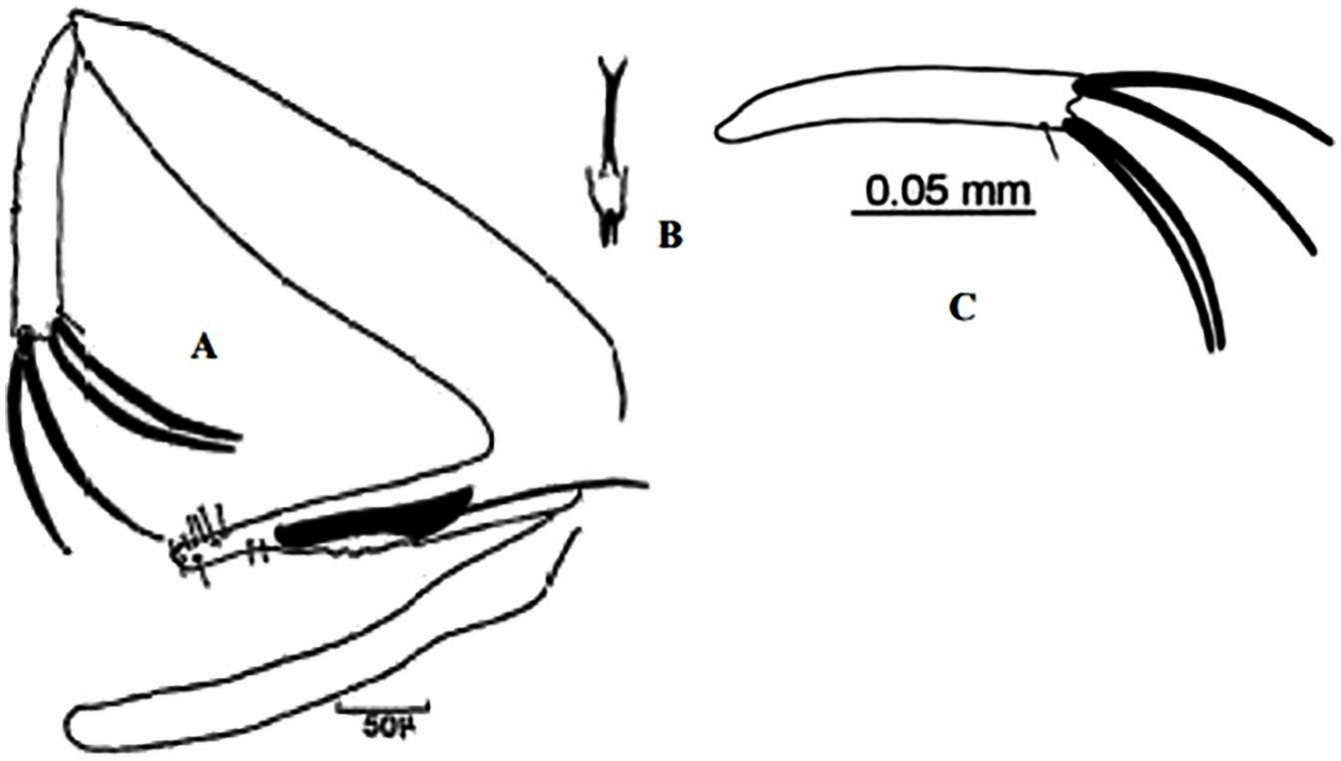
*Sergentomyia fallax* ♂ [44]. **(A)** General genitalia. **(B)** Genitalia pump. **(C)** Insertion of accessory spine.

**Fig 53:**
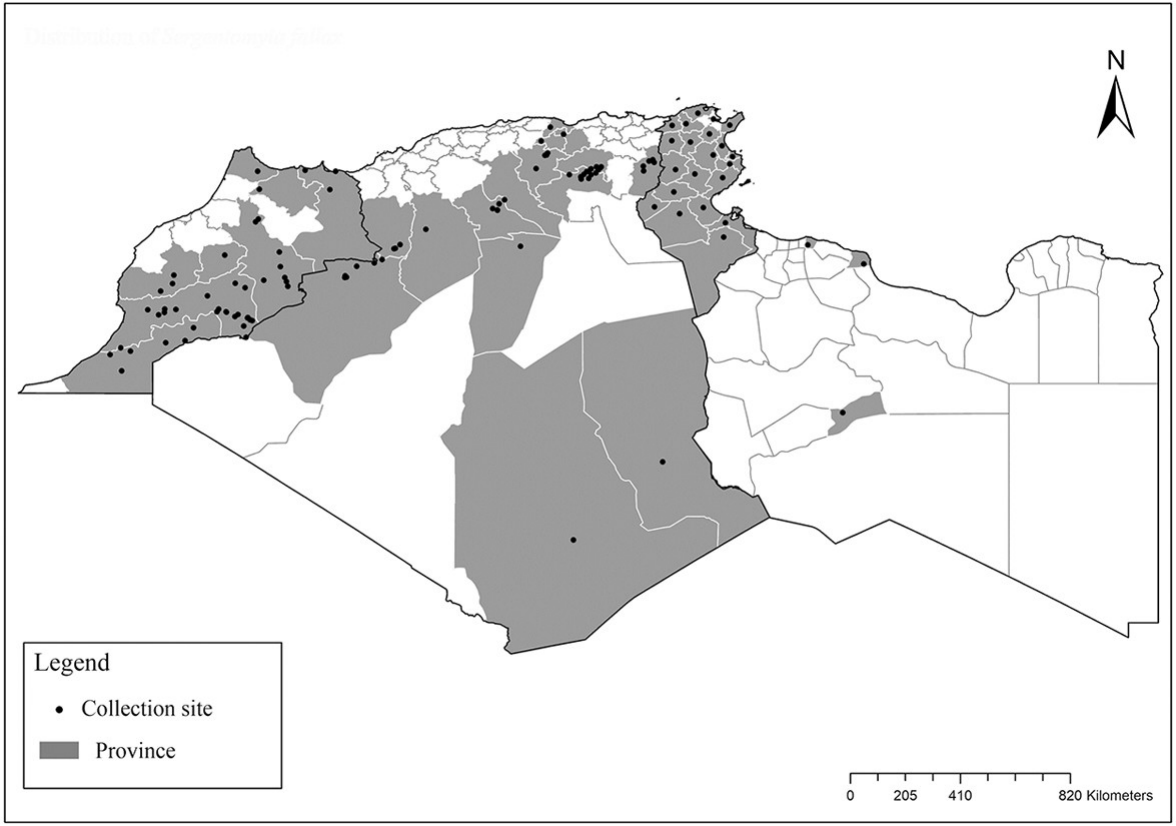
Distribution of *Sergentomyia fallax*. Available from: https://services3.arcgis.com/W1gaXmEpGR8h1K59/arcgis/rest/services/maghreb/FeatureServer.

Females are recognized by strongly distended posterior heart-shaped part of pharynx and the presence of 16 to 18 sharped cibarial teeth aligned on concave shape and oval or rounded pigmented patch (Fig 51). Male style is 4 times longer than wide, with accessory spine in close to distal end (Fig 52) [33]. It occurs in the whole Maghreb region in all bioclimatic zones (Fig 53).

### II-1-3 *Sergentomyia antennata*, Newstead, 1912

**Fig 54:**
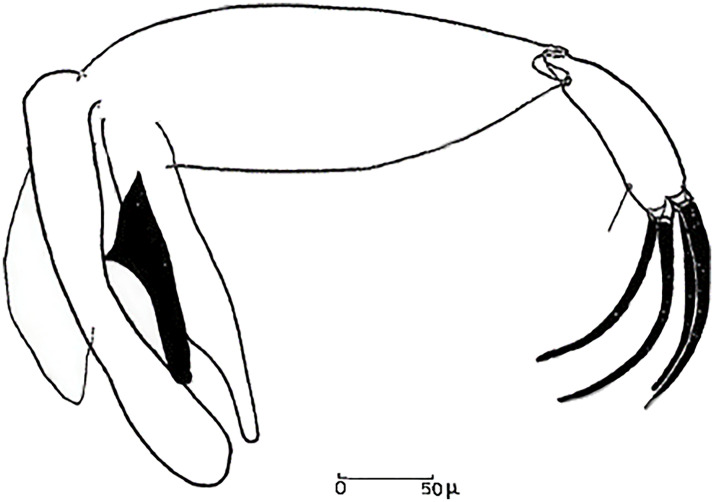
*Sergentomyia antennata* ♂ [44]. General genitalia.

**Fig 55:**
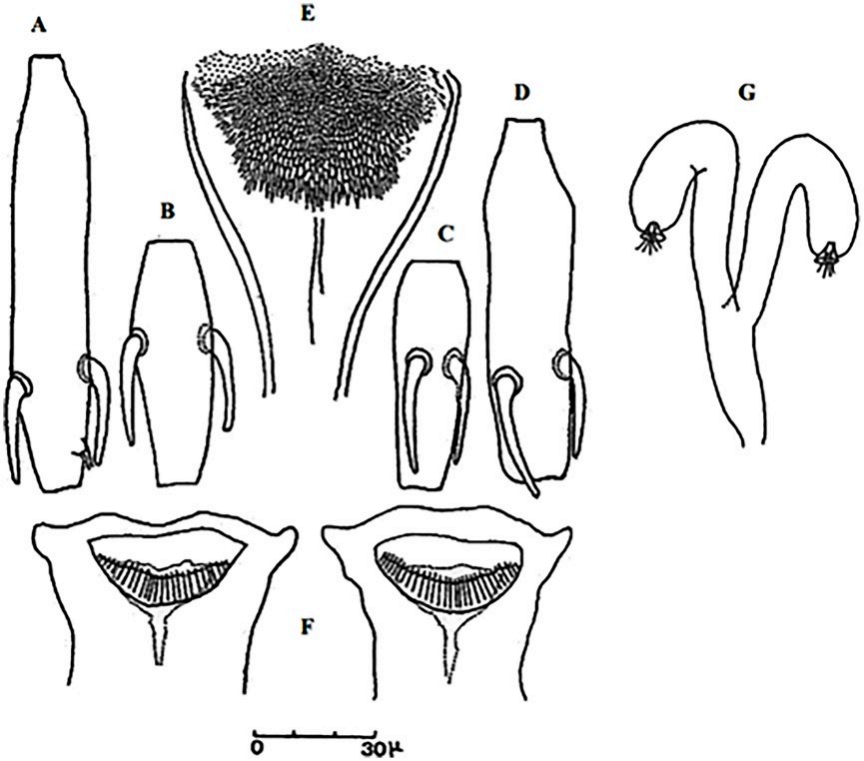
*Sergentomyia antennata*♀ [2]. **(A, B)** Third and fourth antenna segments. **(C, D)** Third and fourth antenna segments. **(E)** Pharynx. **(F)** 2 different aspect of the cibarium. **(G)** Spermathecae.

**Fig 56:**
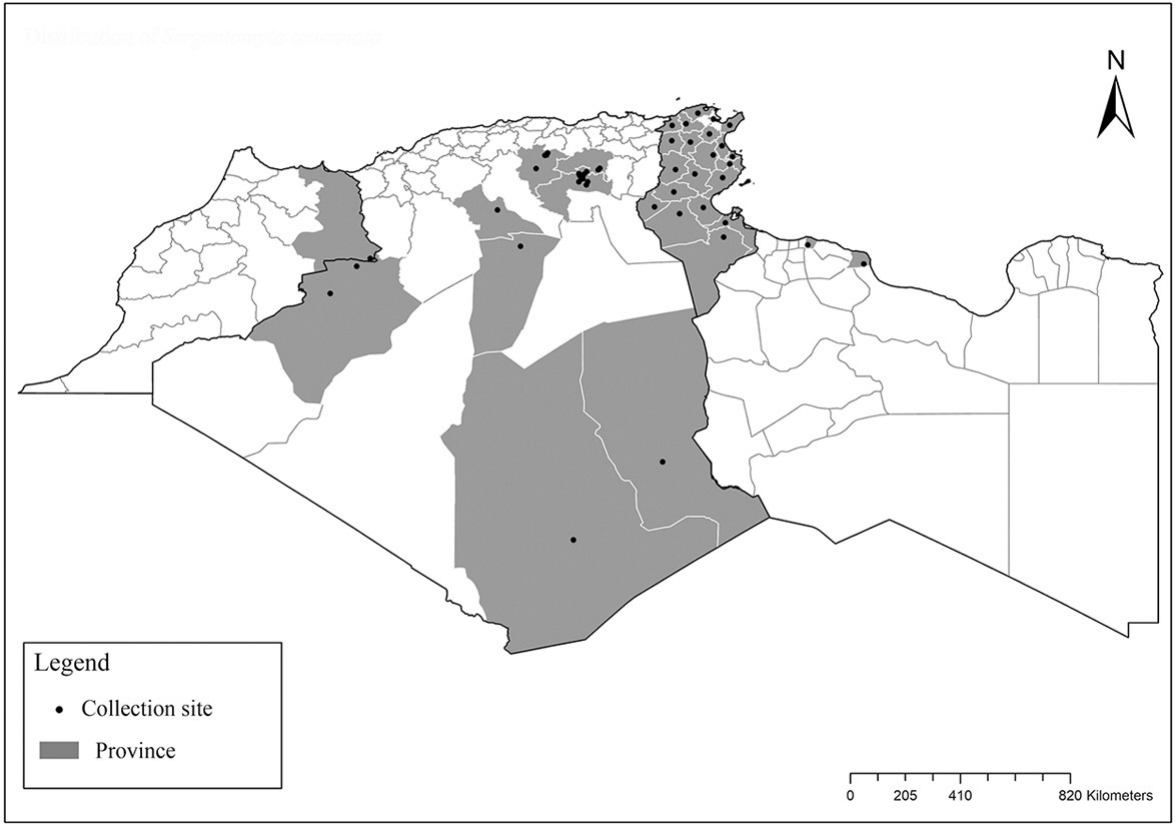
Distribution of *Sergentomyia antennata*. Available from: https://services3.arcgis.com/W1gaXmEpGR8h1K59/arcgis/rest/services/maghreb/FeatureServer.

This species was demonstrated to be also mammaliophilic, feeding on rodents as well as reptiles [33]. Male genitalia are similar to those of *Se*. *minuta*, but the accessory spine is located near the 4 spines (Fig 54). Female possesses 23 to 34 sharped cibarial teeth aligned on a concave shape and triangular pigmented patch (Fig 55). The pharynx has a distended posterior part heart-shaped less marked than *Se. fallax*. *Sergentomyia antennata* also occurs in the whole Maghreb region in all bioclimatic zones (Fig 56).

### II-1-4 *Sergentomyia cincta* Parrot and Martin 1948

**Fig 57:**
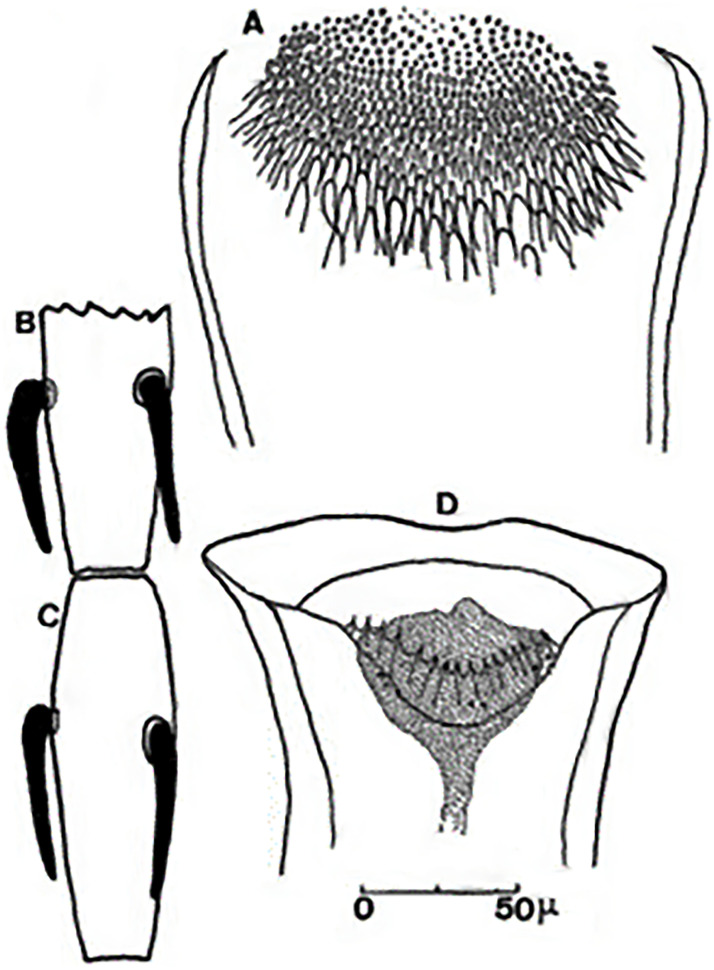
*Sergentomyia cincta*♀ [2]. **(A)** Pharynx. **(B, C)** Third and fourth antenna segments. **(D)** cibarium.

**Fig 58:**
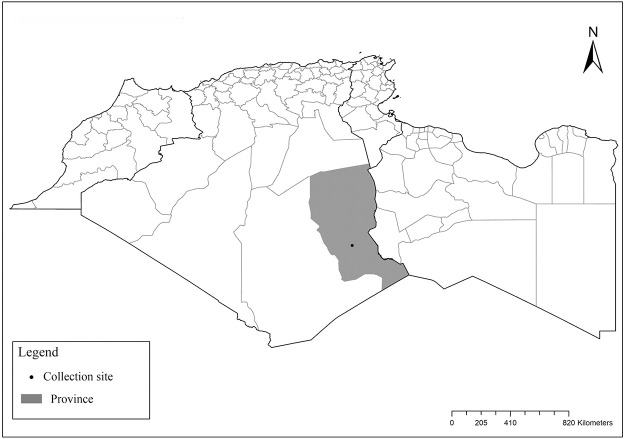
Distribution of *Sergentomyia cincta*. Available from: https://services3.arcgis.com/W1gaXmEpGR8h1K59/arcgis/rest/services/maghreb/FeatureServer.

Its morphology is similar to *Se*. *antennata*. Males are difficult to distinguish but females have only 12 to 18 cibarial teeth (Fig 57) [2]. It is found in the hyperarid bioclimatic zone and reported only in Iherir an Oasis of Algeria (Fig 58). Lewis and Büttiker considered it as synonymous of *Se*. *antennata* [33].

### II-1-5 *Sergentomyia schwetzi*, Adler, Theodor et Parrot, 1929

**Fig 59:**
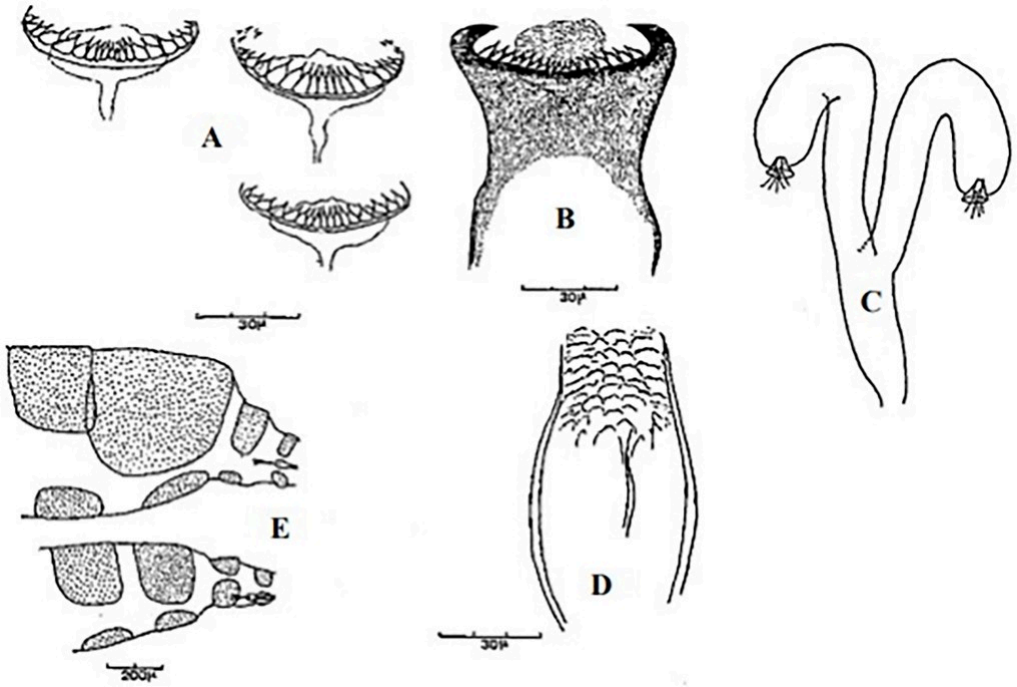
*Sergentomyia schwetzi* ♀ [2]. **(A)** Different cibarium shapes. **(B)** Pharynx. **(C)** Spermathecae. **(D)** Atypical form of the abdomen. **(E)** Typical form of the abdomen.

**Fig 60:**
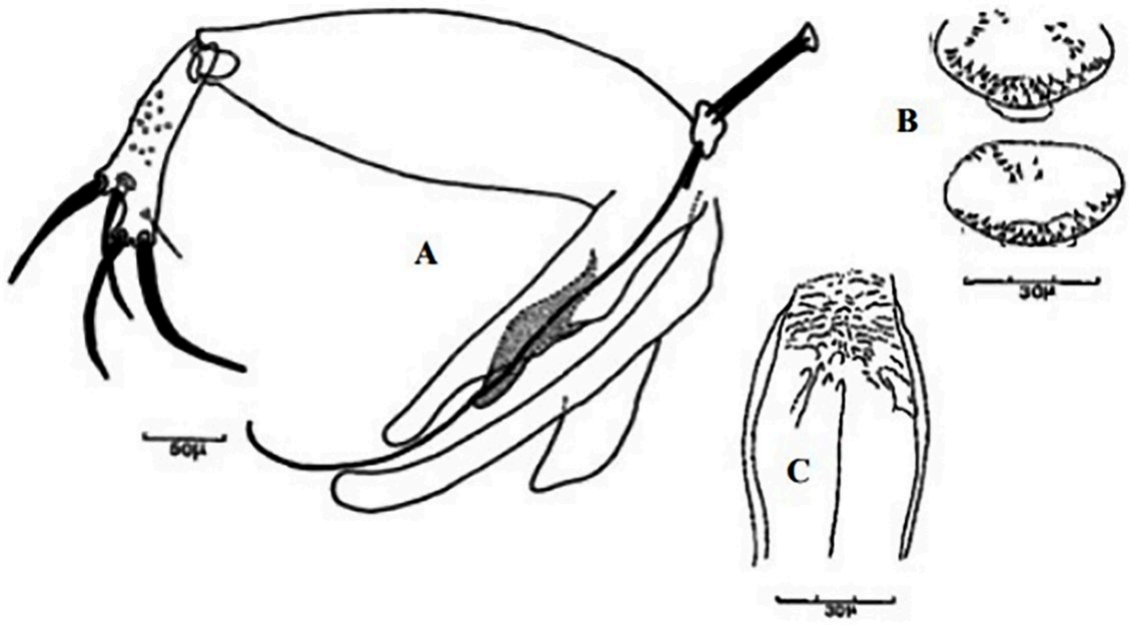
*Sergentomyia schwetzi* ♂ [2,44]. **(A)** General genitalia. **(B)** Different cibarial forms. **(C)** Pharynx.

**Fig 61:**
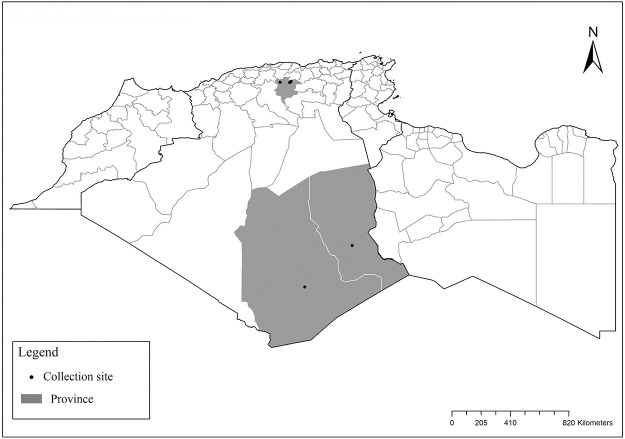
Distribution of *Sergentomyia schwetzi*. Available from: https://services3.arcgis.com/W1gaXmEpGR8h1K59/arcgis/rest/services/maghreb/FeatureServer.

Typical species belongs to the Ethiopian region. Females possess 13 to 20 strong and sharped cibarial teeth aligned on concave shape with pigment patch large in middle (Fig 59). The pharynx has a narrow and strong-armed posterior part (lamp glass shaped). In males, the style holds 4 distal spines arranged in 2 well-separated groups and accessory spine on the distal 1/3. The short and sharped cibarial teeth are arranged in 2 rows, 15 to 20 and 10 to 15, respectively (Fig 60). It was reported only in Algeria and Libya [31,113,114] from subarid to hyperarid bioclimatic zones (Fig 61).

### II-2 Subgenus *Parrotomyia*

Females have cibarium with comb-like row of strong parallel teeth and pharynx narrowing posteriorly (lamp glass shape). The spermathecae have a form of smooth spherical or ellipsoid capsule. Aedeagus is long, slender, triangular, and narrowing gradually to a sharp point, and paramere is hooked [59]. Three species were reported in the Maghreb region.

### II-2-1 *Sergentomyia africana* subsp. *asiatica*, Newstead, 1912

**Fig 62:**
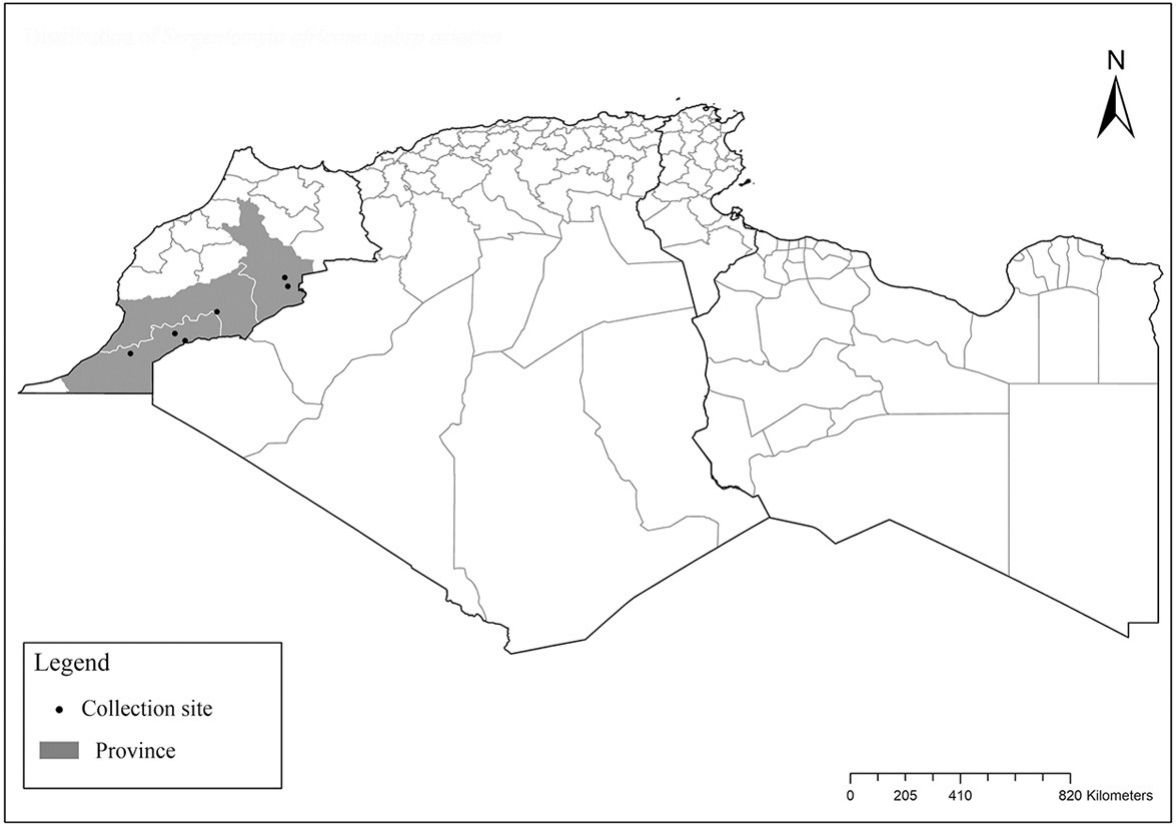
Distribution of *Sergentomyia africana* subsp. *asiatica*. Available from: https://services3.arcgis.com/W1gaXmEpGR8h1K59/arcgis/rest/services/maghreb/FeatureServer.

**Fig 63:**
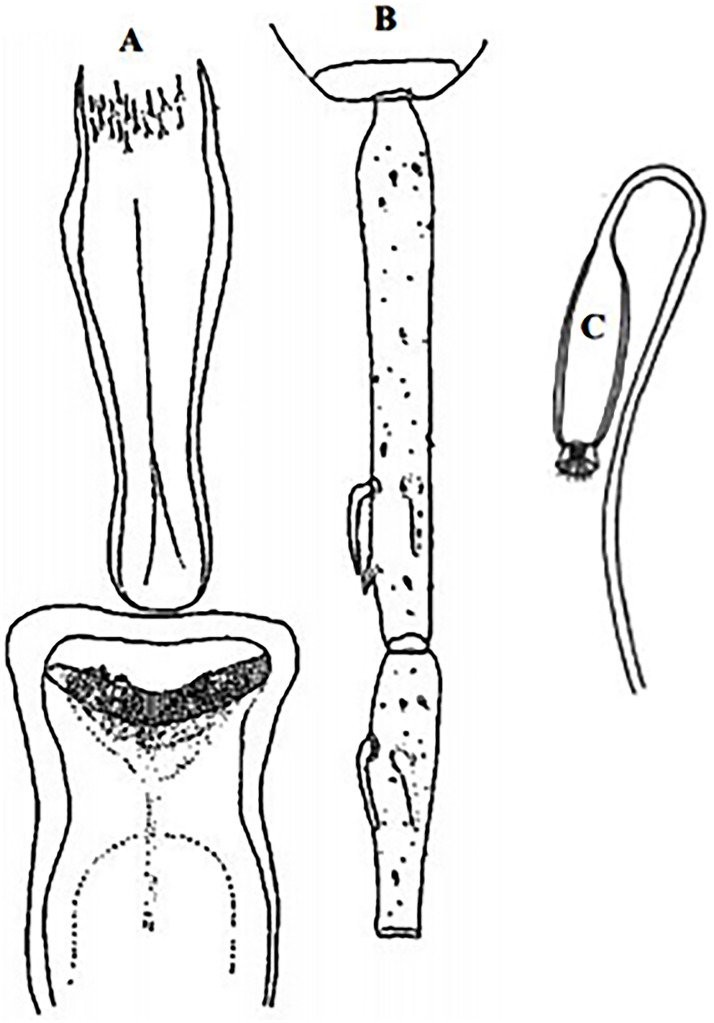
*Sergentomyia africana* subsp *asiatica* ♀ [2]. **(A)** Pharynx and cibarium. **(B)** Third and fourth antenna segments. **(C)** Spermathecae.

This species, also known as *Se*. *africana* var. *cherifiensis*, is distributed in the Ethiopian region, and only 1 female has been reported from Morocco (Fig 62) in the hyperarid bioclimatic zone [24,115]. The spermathecae have an elongated capsule shape; females have 40 to 48 cibarial teeth aligned in a palisade line, 15 punctiform denticules and headband pigmented patch, and their pharynx is long and thread- ike (Fig 63).

### II-2-2 *Sergentomyia africana* subsp. *eremitis*, Parrot et De Jolinière, 1945

**Fig 64:**
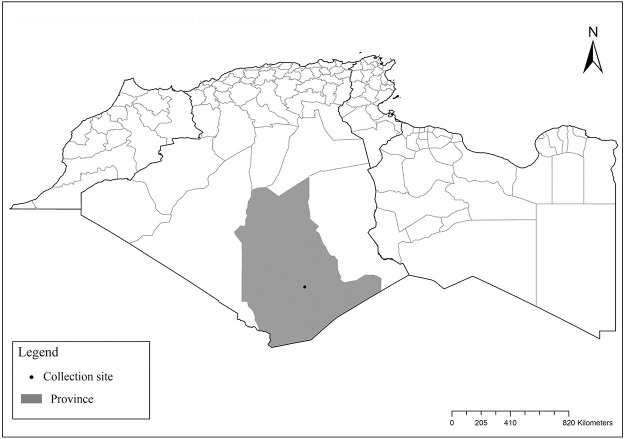
Distribution of *Sergentomyia africana* subsp *eremitis*. Available from: https://services3.arcgis.com/W1gaXmEpGR8h1K59/arcgis/rest/services/maghreb/FeatureServer.

**Fig 65:**
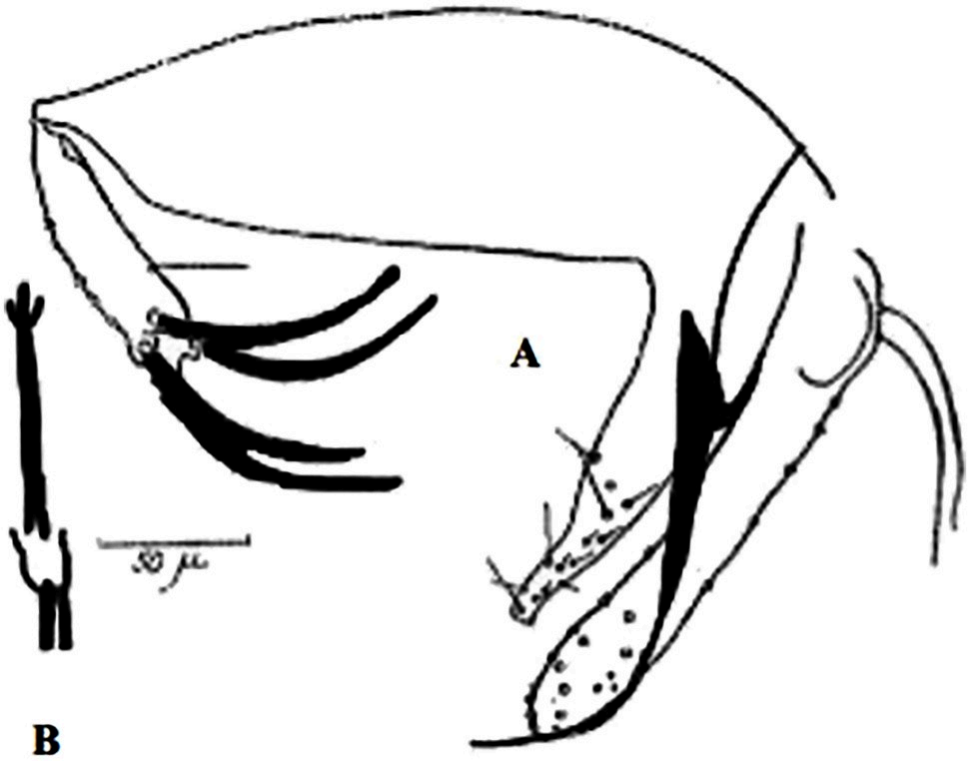
*Sergentomyia africana* subsp *eremitis* ♂ [54]. **(A)** General genitalia. **(B)** Genital pump.

**Fig 66:**
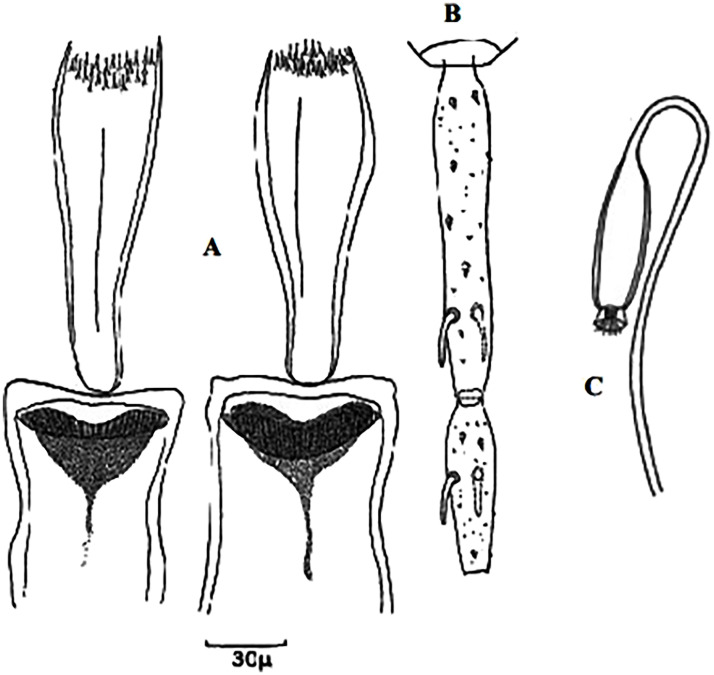
*Sergentomyia africana* subsp *eremitis* ♀ [2]. **(A)** Pharynx and cibarium. **(B)** Third and fourth antenna segments. **(C)** Spermathecae.

Another species of the Ethiopian region [2]. Until now, it was reported only in Algeria (Fig 64) in the hyperarid bioclimatic zone [52]. Male has 30 to 35 cibarial teeth and 8 to 10 denticules disposed on a concave-shape line (Fig 65). Female spermathecae have an elongated capsule shape, 60 to 65 long cibarial teeth aligned on concave-shaped line and 12 punctiform denticules with a mushroom-shaped pigmented patch (Fig 66) [52].

### II-2-3 *Sergentomyia lewisi* Parrot, 1948

**Fig 67:**
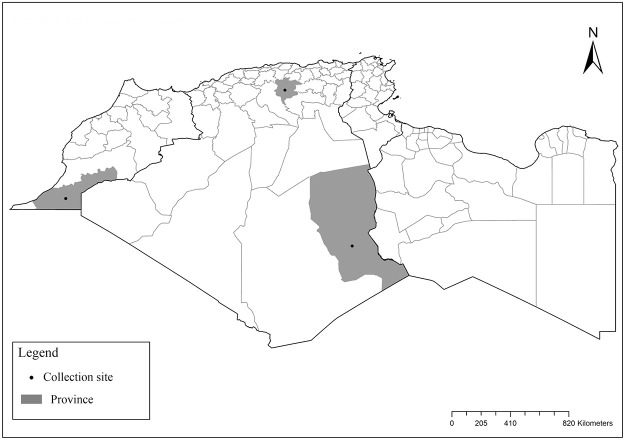
Distribution of *Sergentomyia lewisi*. Available from: https://services3.arcgis.com/W1gaXmEpGR8h1K59/arcgis/rest/services/maghreb/FeatureServer

**Fig 68:**
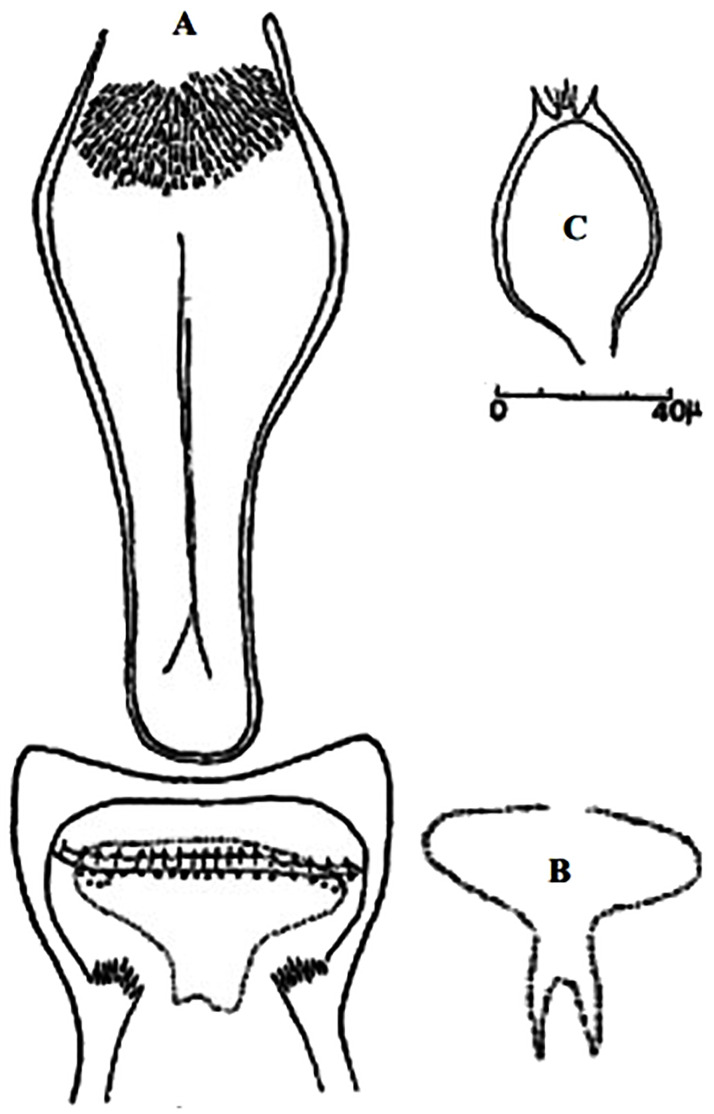
*Sergentomyia lewisi* ♀ [2]. **(A)** Pharynx and Cibarium. **(B)** Pigmentation of the patch. **(C)** Spermathecae.

**Fig 69:**
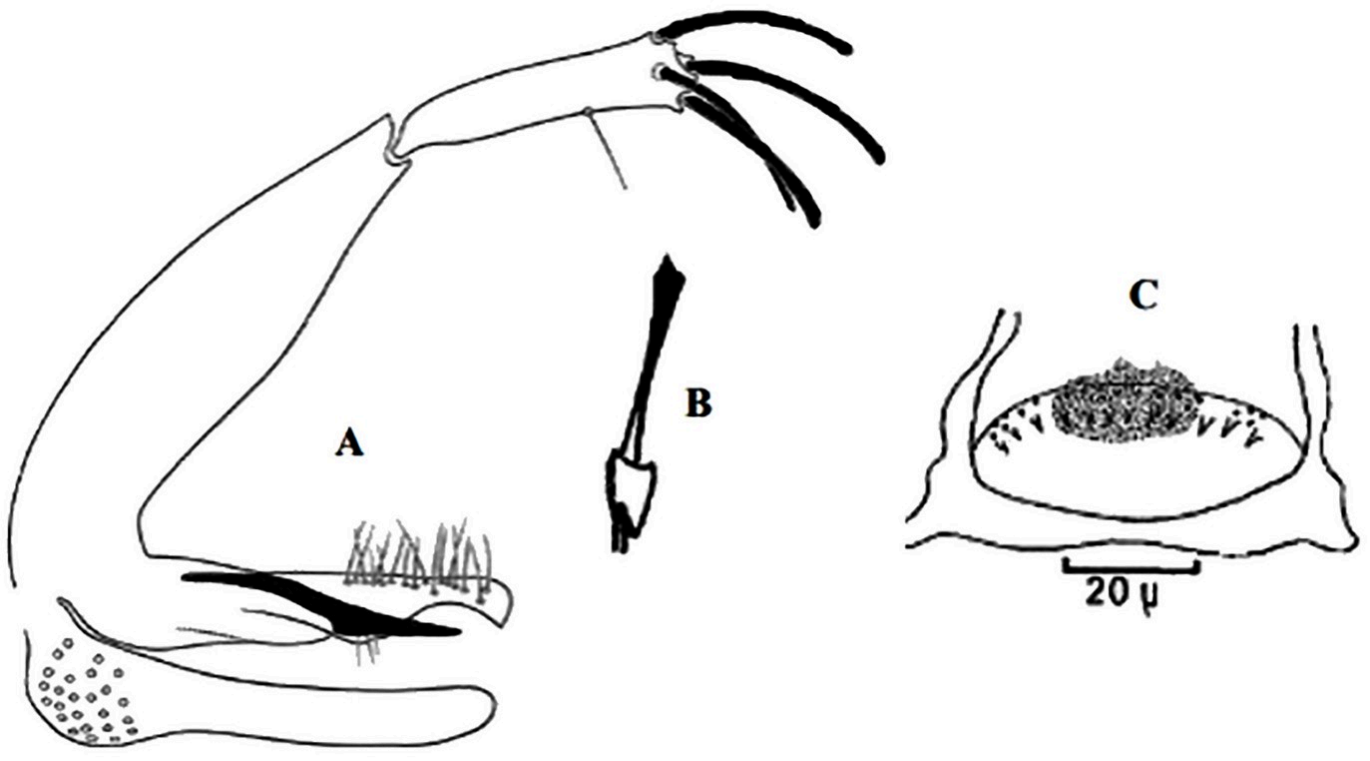
*Sergentomyia lewisi* ♂ [2,57]. **(A)** General genitalia. **(B)** Genitalia pump without pavilion. **(C)** Cibarium.

This species has been reported in Morocco, Algeria, and, recently, in Tunisia [24,38,53] from subarid to hyper arid bioclimatic zones (Fig 67). The females are recognized by the spermathecae with a poppy capsule shape, 15 to 20 strong and sharped cibarial teeth aligned on concave line, 16 to 20 denticules, and mushroom-shaped pigmented patch (Fig 68). Male has 8 to 12 cibarial teeth disposed on a posterior concave line and 8 to 14 denticules (Fig 69) [2].

### II-3 Subgenus *Grassomyia*

Females of this subgenus have spermathecae in form of round, sclerotized capsules with small spicules and their antenna segment 3 lacks ascoids. Females possess cibarial teeth in distinct convex row. Males have simple parameres (not bifid). Species of this subgenus are often associated with vegetation close to water sources and feed on reptiles and amphibians [59,116]. Only a single species is reported in the Maghreb region.

### II-3-1 *Sergentomyia dreyfussi* Parrot, 1933

**Fig 70:**
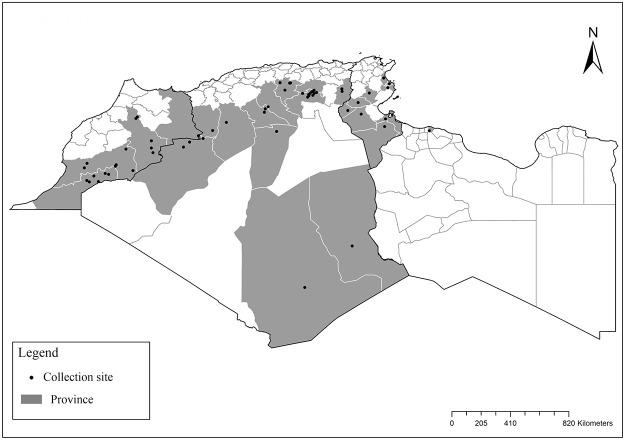
Distribution of *Sergentomyia dreyfussi*. Available from: https://services3.arcgis.com/W1gaXmEpGR8h1K59/arcgis/rest/services/maghreb/FeatureServer.

**Fig 71:**
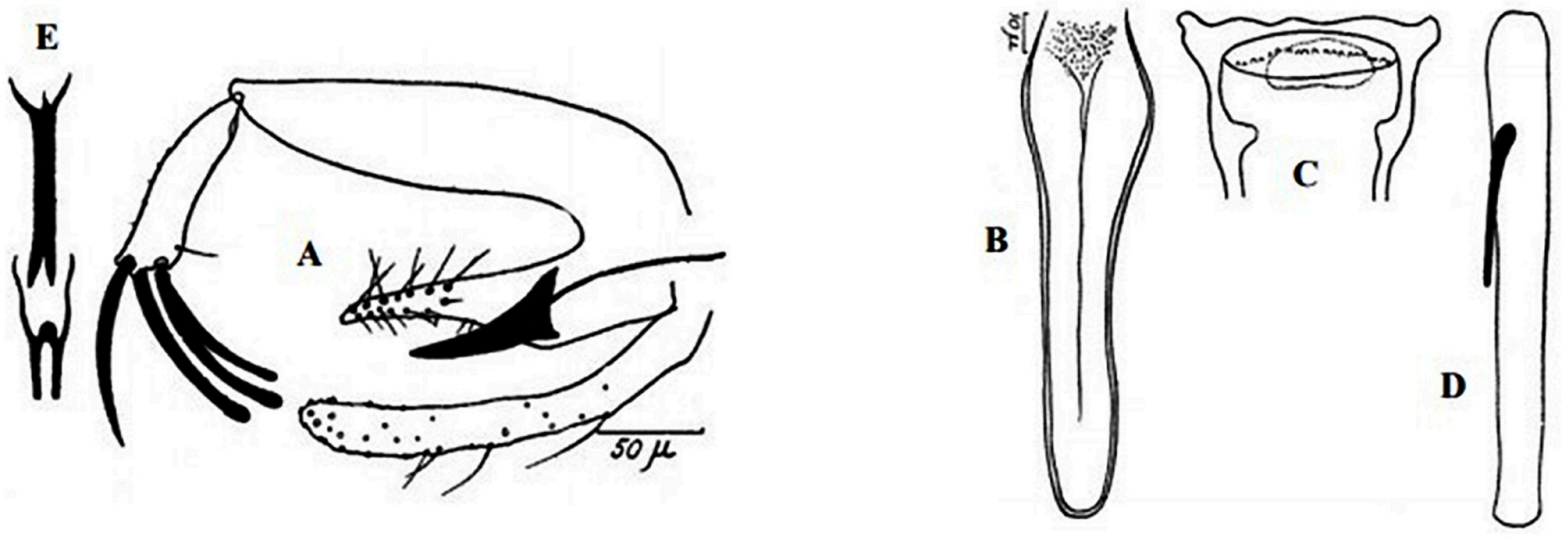
*Sergentomyia dreyfussi* ♂ [54]. **(A)** General genitalia. **(B)** Pharynx. **(C)** Cibarium. **(D)** Third antenna segment. **(E)** Genitalia pump.

**Fig 72:**
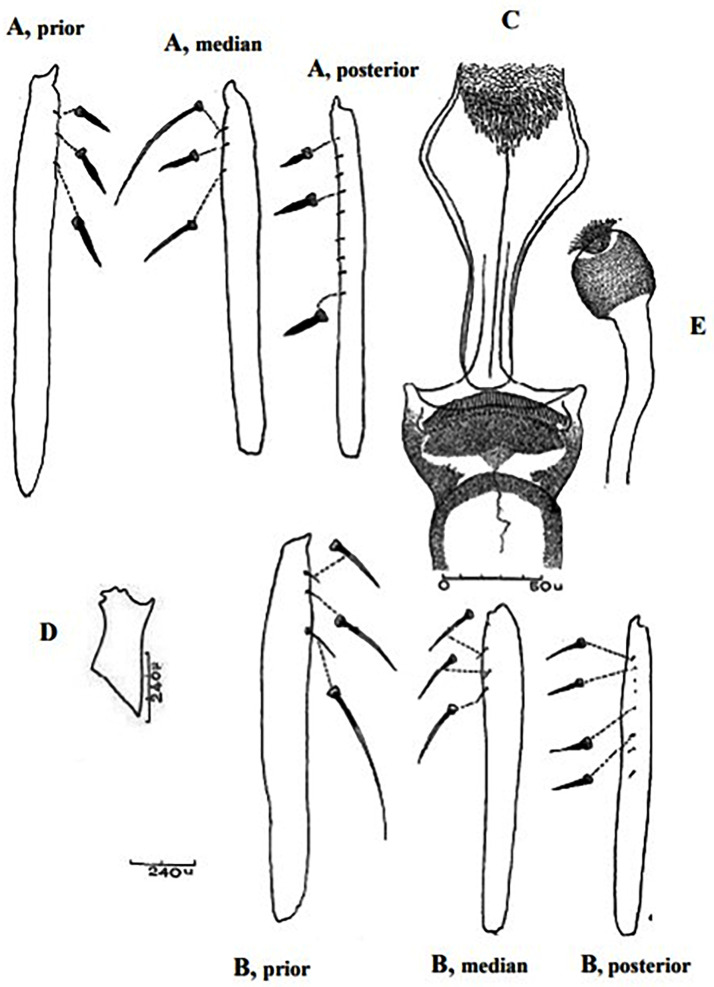
*Sergentomyia dreyfussi* ♀ [2]. **(A, B)** Femurs of Moroccan and Algerian specimens. **(C)** Pharynx and Cibarium. **(D)** Meteplsternum. **(E)** Spermathecae.

This species is reported in the whole Maghreb region (Fig 70) from arid to hyperarid bioclimatic zones [37,41,51]. Males have a style with 4 strong terminal spines, accessory spine located close to them, strong and triangular (conical) aedeagus, 20 separated cibarial teeth, and presence of spines on the femur of the first leg (Fig 71). The females possess characteristic rounded spermathecae covered by thin spicules (Fig 72) [51].

### II-4 Subgenus *Sintonius*

Species with scanty erect hairs on the dorsal aspects of segments II to VI in females with segmented or crenulated spermathecae shaped similarly to subgenus *Phlebotomus*. Males have a pointed aedeagus and hooked intermediate appendage, [2,117]. Four species are reported from the Maghreb region.

### II-4-1 *Sergentomyia clydei* Sinton, 1928

**Fig 73:**
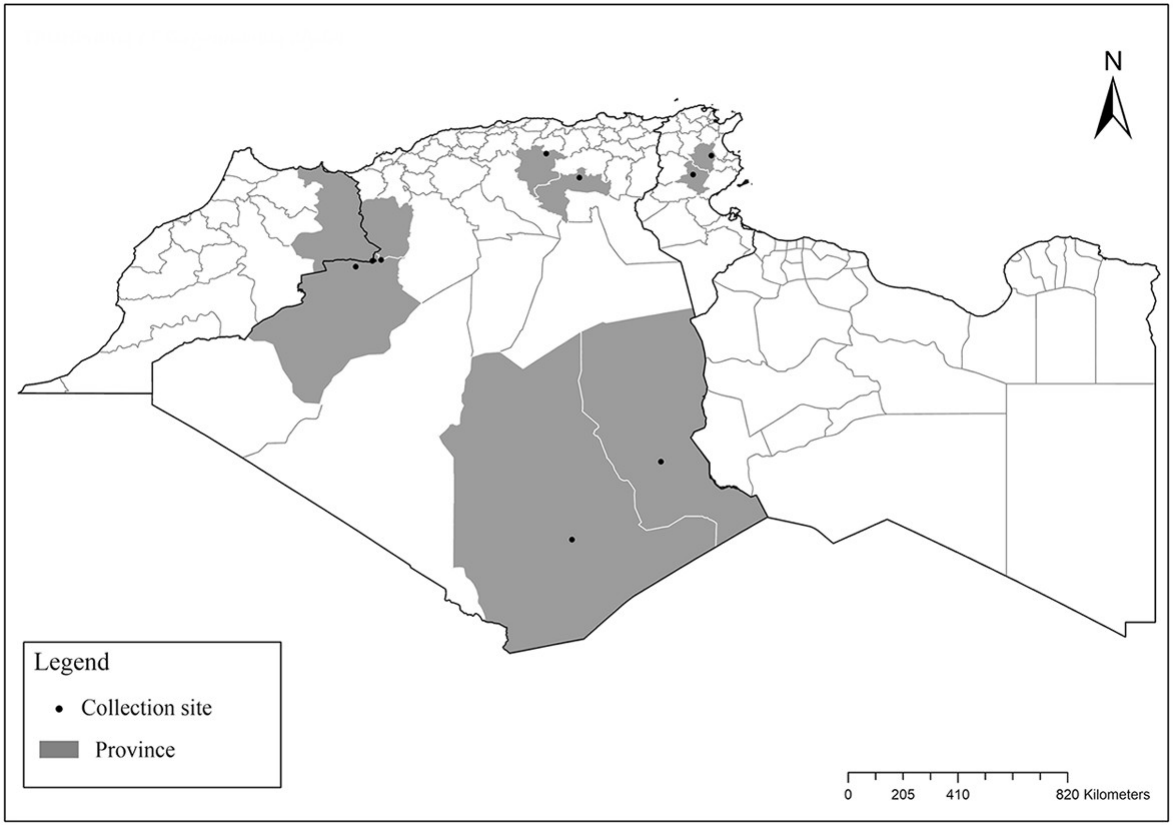
Distribution of *Sergentomyia clydei*. Available from: https://services3.arcgis.com/W1gaXmEpGR8h1K59/arcgis/rest/services/maghreb/FeatureServer

**Fig 74:**
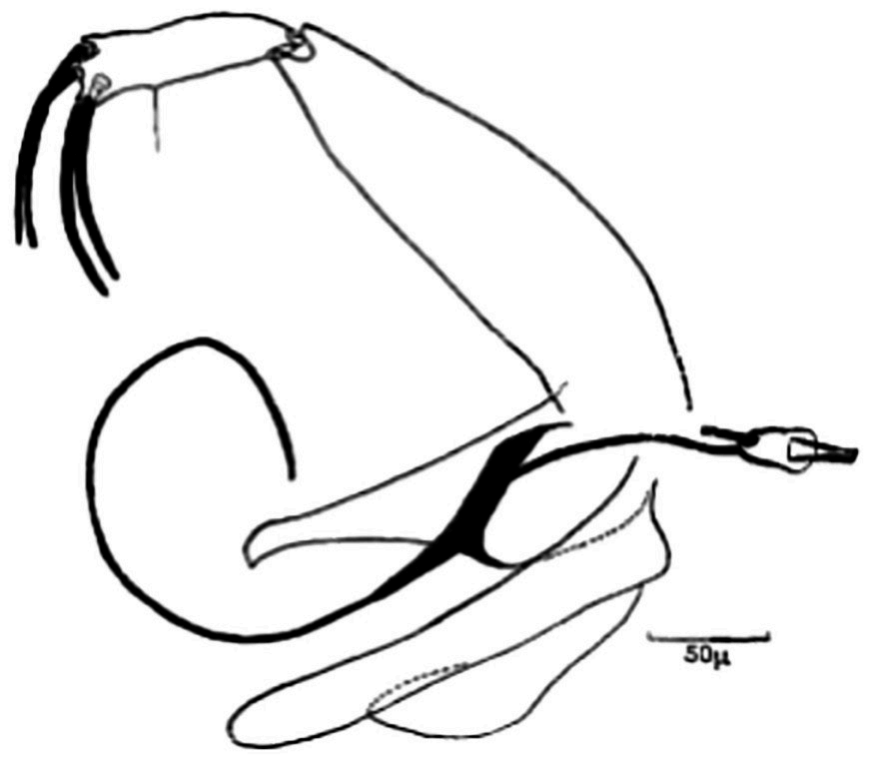
*Sergentomyia clydei* ♂ [2]. General genitalia.

**Fig 75:**
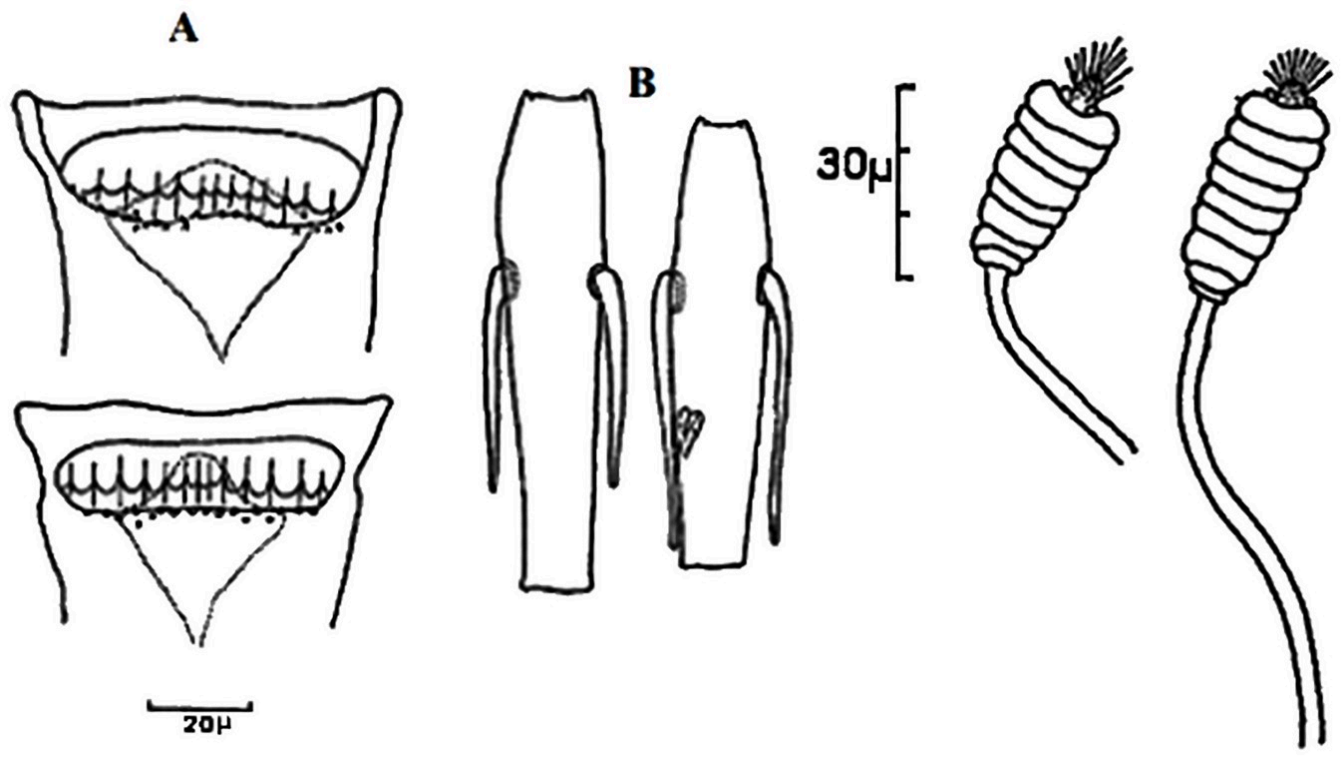
*Sergentomyia clydei* ♀ [2]. **(A)** Cibarium. **(B)** Third and fourth antenna segments. **(C)** Spermathecae.

This species was reported to transmit *Sauroleishmania* sp. and DNA of *L*. *major* was recently detected in specimens from Tunisia [118]. This species is reported in the whole Maghreb region (Fig 73) from arid to hyperarid bioclimatic zones [37,41,119]. The males are recognized by the presence of 4 strong spines, 2 apical and 2 subapical, and an accessory spine on the distal 1/3, 2 to 3 strong teeth and 25 to 35 denticules. The aedeagus is short, conical, and pointed (Fig 74). This species is closely related to *Se. christophersi*, which differs only in the denticules number [119]. In the females, the spermathecae have 7 or 8 segments. The cibarium includes 2 rows of teeth, 13 long and sharped and 18 short and blunt with a diamond pigmented patch (Fig 75) [2].

### II-4-2 *Sergentomyia christophersi* Sinton, 1927

**Fig 76:**
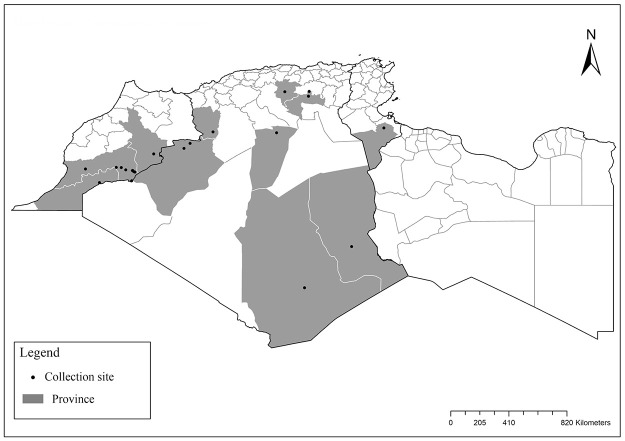
Distribution of *Sergentomyia christophersi*. Available from: https://services3.arcgis.com/W1gaXmEpGR8h1K59/arcgis/rest/services/maghreb/FeatureServer.

**Fig 77:**
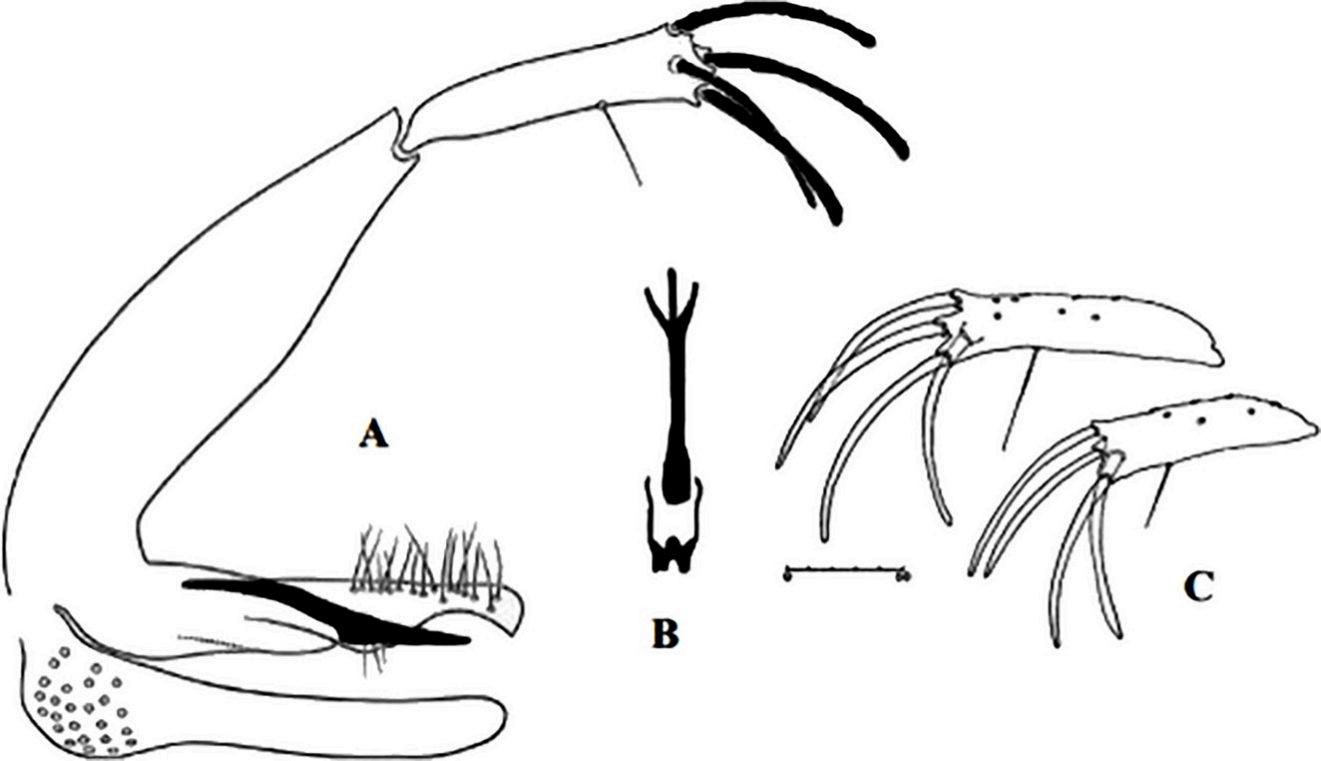
*Sergentomyia christophersi* ♂ [2,48]. **(A)** General genitalia. **(B)** Genital pump. **(C)** Insertion position of accessory spine.

**Fig 78:**
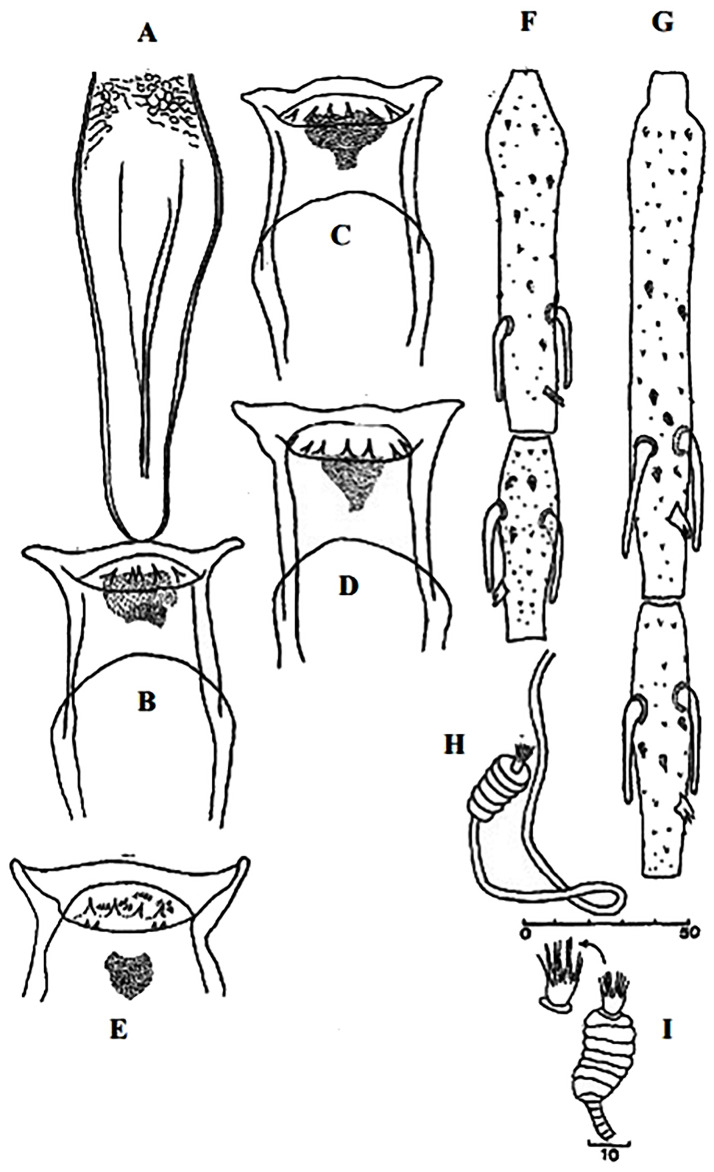
*Sergentomyia christophersi* ♀ [2]. **(A)** Pharynx. **(B–D)** Cibarium Guinean specimens. **(E)** Cibarium Tibetan specimen. **(F)** Third and fourth antenna segments, Guinean specimen. **(G)** Third and fourth antenna segments, Tibetan specimen. **(H)** Spermathecae, Tibetan specimen. **(I)** Spermathecae Guinean specimen.

Originally described from Pakistan by Sinton [120], this species has been reported first in Tunisia in 1971. It occurs in all Maghreb region (Fig 76) between the arid and hyperarid bioclimatic zones [33,45,95,121]. The males are similar to *Se. clydei* except the number of denticules that equals to 05 (Fig 77). In females, the spermathecae have 8 segments, 4 to 7 sharped and pointed cibarial teeth, 4 to 5 denticules, and small triangular pigmented patch (Fig 78) [2].

### II-4-3 *Sergentomyia hirtus* Parrot et de Jolinière, 1945

**Fig 79:**
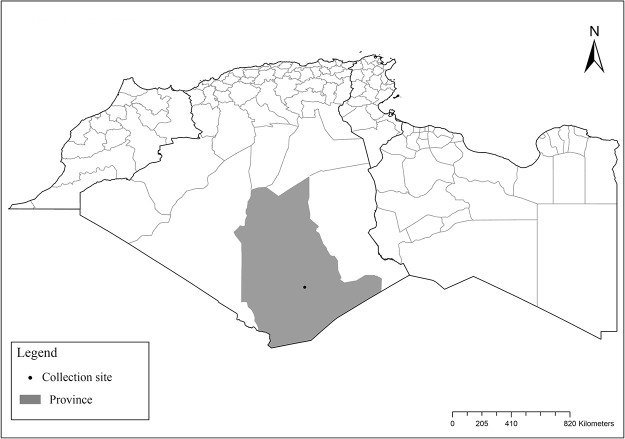
Distribution of *Sergentomyia hirtus*. Available from: https://services3.arcgis.com/W1gaXmEpGR8h1K59/arcgis/rest/services/maghreb/FeatureServer.

**Fig 80:**
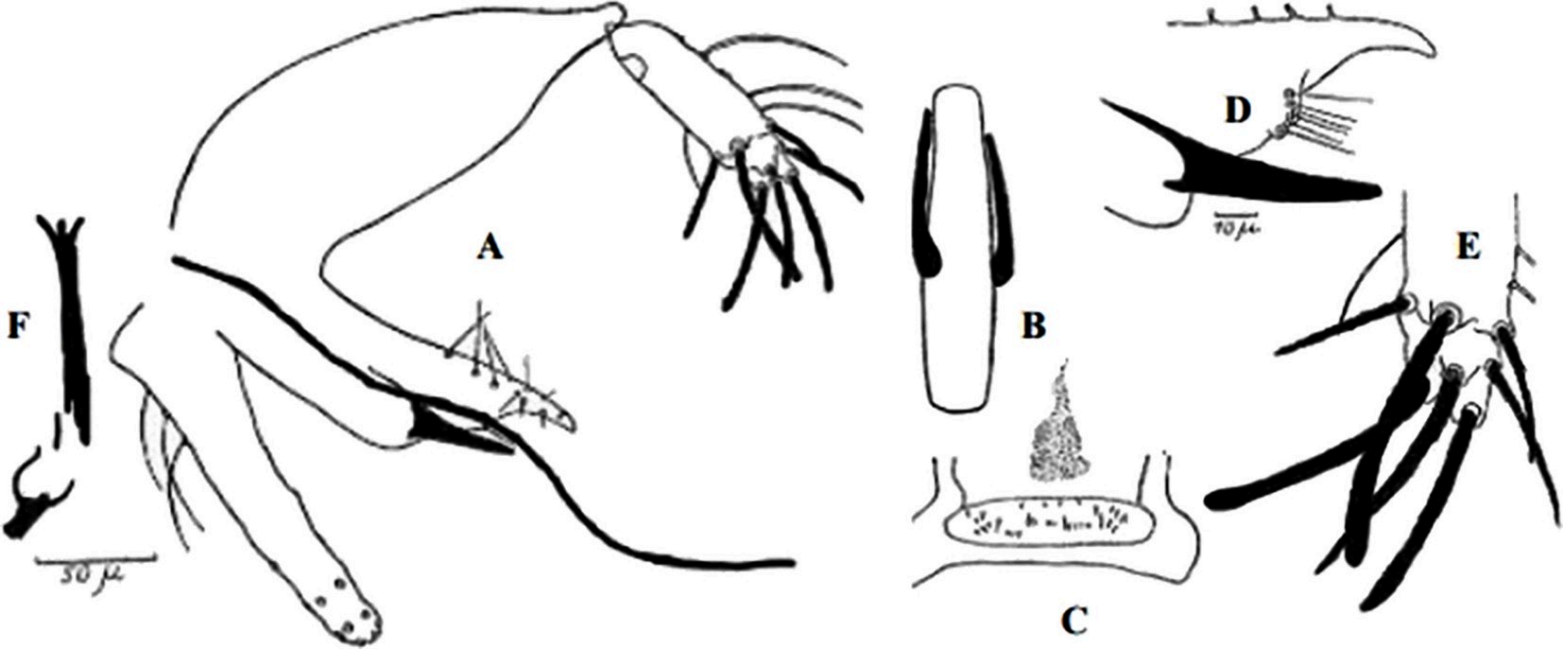
*Sergentomyia hirtus* ♂ [55]. **(A)** General genitalia. **(B)** Fourth antenna segment. **(C)** Cibarium. **(D)** Aedeagus. **(E)** Style. **(F)** Genitalia pump.

This species was described based on a single male captured in In-Amguel (Tamanrasset) in Algeria in the hyperarid bioclimatic zone (Fig 79). Its style holds 7 strong spines (3 submedians, 2 subapical, and 2 apical) and an accessory spine. The aedeagus is very short (18 μm) and subconical (Fig 80). The females remain unknown [52]. Dedet and colleagues [33] challenged the validity of the species since it was not reported from elsewhere and could be just a malformation often reported in *Sergentomyia* species.

### II-4-4 *Sergentomyia tiberiadis* Adler, Theodor, and Lourie, 1930

**Fig 81:**
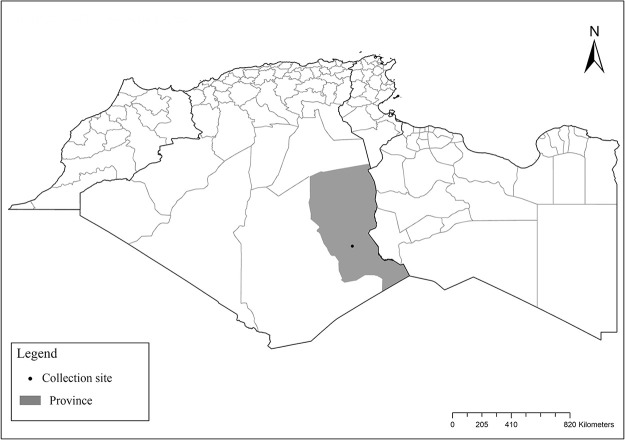
Distribution of *Sergentomyia tiberiadis*. Available from: https://services3.arcgis.com/W1gaXmEpGR8h1K59/arcgis/rest/services/maghreb/FeatureServer.

**Fig 82:**
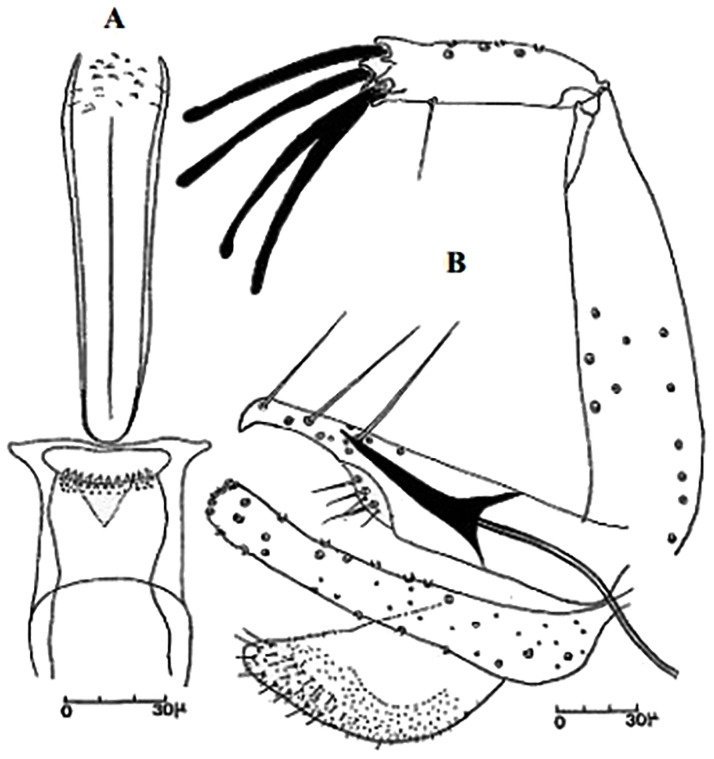
*Sergentomyia tiberiadis* ♂ [2]. **(A)** Pharynx and Cibarium. **(B)** General genitalia.

**Fig 83:**
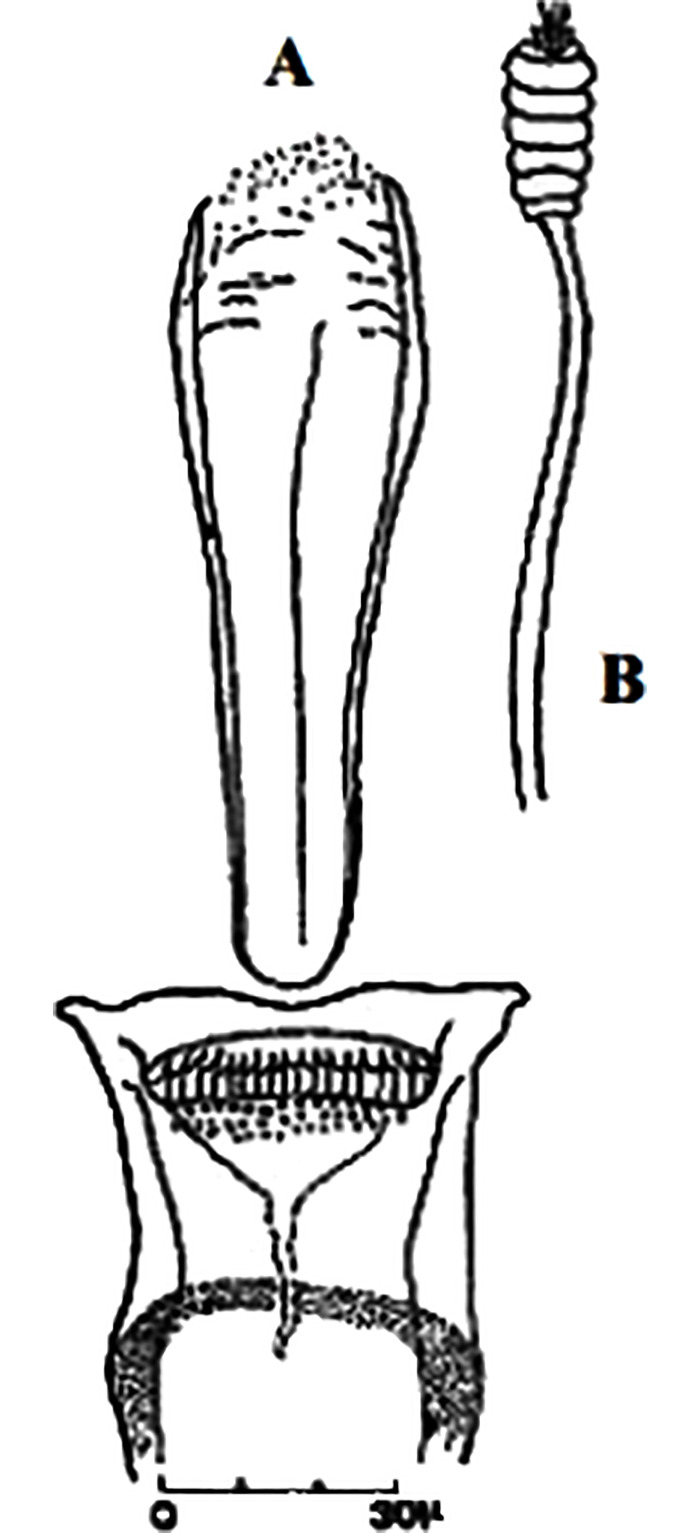
*Sergentomyia tiberiadis* ♀ [2]. **(A)** Pharynx and cibarium. **(B)** Spermathecae.

This species was reported only in the Central Sahara of Algeria where the hyper arid bioclimatic zone occurs (Fig 81). Previously, it was named as *Se*. near *clydei* before considering it as a new species [122]. The males have a long, slender, and pointed end aedeagus without subapical tubercle, 12 to 14 pointed cibarial teeth standing almost in straight line and about 15 punctiform denticules arranged in a zigzag pattern (Fig 82). In females, the spermathecae comprise 6 to 8 segments, narrowing toward the apex, 16 to 17 wide and pointing upward cibarial teeth arranged in a line slightly convex interiorly, and about 10 to 15 punctiform denticules arranged in zigzag row. The pharynx seems completely unarmed (Fig 83) [2,122].

## Discussion

Our study reviews the presence of sand fly species in Morocco, Algeria, Tunisia, and Libya countries where sand fly fauna is known to be very diverse, and many species are incriminated in the transmission of sand fly–borne pathogens as arboviruses of the *Phlebovirus* group [123] and most importantly parasitic protozoa of the genus *Leishmania*. We decided to exclude Mauritania from the analyses because accurate data regarding the distribution of sand fly species and the incidence of CL and VL are unavailable. To our best knowledge, we summarize all sand fly species recorded in the Maghreb region for the first time. We also update the information concerning their vector role in transmission of diseases, morphological characters important for species identification and their biology.

The first sand fly species reported from North Africa was *Ph*. *papatasi* in 1908 [124]. Since then, a plethora of studies reported presence of so far 32 sand fly species, 16 species of the genus *Phlebotomus* and 16 species of the genus *Sergentomyia*. The genus *Phlebotomus* is represented by 4 subgenera, namely *Larroussius*, *Phlebotomus*, *Paraphlebotomus*, and *Transphlebotomus* and the genus *Sergentomyia* comprises 5 subgenera, namely *Sergentomyia*, *Parrotomyia*, *Grassomyia*, *Sintonius*, and *Parvidens*, species of the last one recorded only in Mauritania. The importance of the Maghreb region for sand fly research is highlighted by the fact that within the 32 species recorded here, 13 species were first described in one of the Maghreb region countries [116]: male of *Ph. sergenti* Parrot, 1917 [125], male of *Se*. *fallax* Parrot, 1921, male of *Ph*. *perfiliewi* Parrot, 1930, male of *Ph. langeroni* Nitzulescu, 1930 [99], male and female of *Ph*. *longicuspis* Nitzulescu, 1930 [126], male and female of *Se. dreyfussi* Parrot, 1933, male and female of *Ph*. *bergeroti* Parrot, 1934, male and female of *Se. eremitis* Parrot et De Jolinière, 1945 [52], male of *Se. hirtus* Parrot et de Jolinière, 1945 [52], male of *Ph. chadlii* Rioux, Juminer et Gibily, 1966 [94], male of *Ph*. *chabaudi* Croset, Abonnenc, and Rioux, 1970 [55], male of *Ph. mariae* Rioux, Croset, Léger et Bailly-Choumara, 1974 [25] and finally, male and female of *Ph*. *riouxi* Depaquit, Léger, and Killick-kendrick, 1998 [48].

The 4 reviewed countries within the region share some ubiquitous sand fly species, namely *Ph*. *papatasi*, *Ph*. *bergeroti*, *Ph*. *perniciosus*, *Ph*. *longicuspis*, *Ph*. *langeroni*, *Ph*. *sergenti*, *Ph. alexandri*, *Ph*. *chabaudi*, *Se*. *antennata*, *Se*. *christophersi*, *Se*. *clydei*, *Se*. *fallax*, *Se*. *dreyfussi*, *Se*. *minuta*, and *Se. schwetzi*. Algeria and Morocco have a record of 24 and 23 species, respectively [15,27,32,127]. Several rare species were reported only in Algeria: *Ph. mascittii*, *Se*. *africana* subsp *eremites*, *Se. tiberiadis*, and *Se*. *hirtus* [32,33,52], whereas *Ph*. *mariae* and *Se*. *africana* subsp. *asiatica* were reported only from Morocco [24,25]. Tunisia is ranked at third place with 18 species so far [38]. For Libya, we are inclined to count 15 species, excluding 6 other species reported without any morphological evidence: *Ph. orientalis*, *Ph. tobbi*, *Se. bedfordi*, *Se. cineta*, *Se. adleri*, and *Se. palestiniensis* [113]. Record of these species was later considered as not sufficiently proven [41,121]. So far, the proven vectors of *Leishmania* parasite reported in Libya are *Ph*. *papatasi*, *Ph*. *perniciosus*, *Ph*. *langeroni*, *Ph*. *perfiliewi*, and *Ph*. *sergenti* [69]. Interestingly, the study carried out in Libya [41] provides illustration of some male specimens identified as *Larroussius* sp. that in our opinion displayed an aedeagus with curved tip characteristic of *Ph*. *perniciosus* atypical form; unfortunately, the author passed beside the right identification despite that the atypical forms were clearly described in Spain and Morocco since 1991 and 1998, respectively [83,128]. The studies considering the atypical forms of *Ph*. *perniciosus* were focused mostly on its molecular and morphological characterization and how to discriminate it from *Ph*. *longicuspis* [30,83,128–130]. So far, this species was reported in Morocco, Algeria, and Tunisia trapped in sympatric with *Ph*. *longicuspis* and *Ph*. *perniciosus* [30,83,84]. By contrast, relatively little is so far known about its ecology, host preferences, behavior, and vectorial capacity. These important aspects shall be addressed in future research in order to understand better its potential in the transmission of VL. In Mauritania, 10 sand fly species were historically reported: *Ph*. *bergeroti*, *Ph. duboscqi*, *Se. dubia*, *Se. africana africana*, *Se*. *antennata*, *Se*. *schwetzi*, *Se*. *leslyae*, *Se. magna*, and *Se. freetownensis* [2,116,131]. However, as these data stem exclusively from general keys and catalogs and no results of dedicated entomological surveys are available from this country, we cannot evaluate its sand fly fauna in more details. The update of this scant knowledge is much desired as 6 of the 10 Mauritanian species (*Ph*. *duboscqi*, *Se*. *dubia*, *Se*. *africana africana*, *Se. lesleyae*, *Se*. *magna*, and *Se*. *freetownensis*) were not reported from any other Maghreb country.

It is important to note that the current known distributions of sand fly species within the Maghreb region are not static but rather prone to dynamic changes. The countries share the same climatic conditions and landscape environments in large borderland areas, allowing the exchange of species either naturally or via human activities such as trade (e.g., cattle manure for the soil fertilization) and movement of the populations due to the drought or political crises across the Maghreb and north of the Sahel region. These factors may contribute to a potentially rapid spread of both pathogens and their vectors. Moreover, ongoing climate change may further facilitate the adaptation of arthropods and emergence of arthropod-borne pathogens in new areas [132]; this hypothesis can be supported by the occurrence of *Anopheles gambiae*, a primary vector of malaria in Africa, in the Algerian territory at the Malian border [133], although this species was originally not expected to be able to cross the Saharan band of the Sahel region. Sand fly distribution is known to be impacted by the climatic conditions mainly temperature and humidity. Under the scenarios of a prediction model assessing the impact of potential climate change on the distribution of sand fly populations, expected rising temperatures may allow the expansion of sand fly populations into environmentally suitable areas north off the current Mediterranean distribution [134]. Based on recent climate change predictions, North Africa region is expected to become more arid by 2100 when only the coastal parts will retain their semihumid bioclimatic zone [135]. Subsequently, some species such as *Ph*. *papatasi*, *Ph*. *alexandri*, *Ph*. *sergenti* and *Ph*. *ariasi*, while gaining more new areas by their northward expansion into currently nonendemic regions of Europe, may disappear from some regions in North Africa [136]. Such scenarios allow us to expect some species from the arid part expanding further north of their current distribution, possibly contributing to enhance transmission of various pathogens.

As already described, both cutaneous and visceral leishmaniases constitute one of major public health problems in the Maghreb region: While they were historically established here, recently, these diseases show a significant rise through many local outbreaks and thousands of new human cases annually. This is partially facilitated by abundant sand fly fauna as in each country, several proven and/or suspected sand fly vectors are present: *Ph*. *papatasi* transmitting *L*. *major*, *Ph*. *perniciosus*, and *Ph*. *perfiliewi* transmitting *L*. *infantum*, *Ph*. *sergenti* transmitting *L*. *tropica* and *Ph*. *longicuspis*, which is suspected to transmit *L*. *infantum* [21]. It is important to note that while firm criteria for rigorous incrimination of a sand fly species as a vector were set [1], these are often difficult to meet for practical and logistical reasons. The increasing availability of molecular methods of pathogen detection in sand fly vectors lead to widespread use of these techniques in the screening of field-caught specimens for *Leishmania* detection, this including a majority of studies in Maghreb. However, such findings shall be considered with caution as a detection of *Leishmania* DNA does not prove the role of analyzed sand fly species in its transmission. Typically, species of the genus *Sergentomyia* were often suspected to be incriminated in the transmission of *Leishmania* sp. based on circumstantial evidence [114]. Thus, it is important to determine the potential vectorial role of certain species especially in the central Sahara where *Sergentomyia* species are more abundant than species of the genus *Phlebotomus* [110]. Despite it was demonstrated that *Se*. *schwetzi* did not support the development of *Leishmania* parasites (including *L*. *major* and *L*. *infantum*) under laboratory conditions [137], such experimental assessment of *Sergentomyia* species remains rare and should deserve more attention in the future studies.

Several arboviruses of the order Bunyaviridae were reported from the Maghreb countries, including well-characterized TOSV, which is of most importance as it infects humans and was determined as a causative agent in a human case in Tunisia [138], Naples virus and Sicilian virus. However, despite the fact that these 3 sand fly–borne viruses known from other parts of the Mediterranean pose a potential human health risk, they remain understudied compared to leishmaniases: A recent literature review reported that of all publications on TOSV, only 8 refer to Algeria, 4 to Morocco, and 13 to Tunisia, while no data are available from Libya and Mauritania [139]. This contrasts with the expectation that these viruses may in fact be very prevalent in the region. High rates of TOSV-neutralizing antibodies reported from studies performed on humans or domestic animals in Algeria [87,93,140] and Tunisia [141,142] suggest frequent circulation of the virus in the region; in fact, some of human seroprevalence studies demonstrated percentage of human population that possess neutralizing antibodies against TOSV to be 2 to 3 times higher than in southern Europe [143]. The 3 abovementioned phleboviruses were detected in sand fly species of several subgenera in the Maghreb region as summarized in Table 1: TOSV in *Ph*. *papatasi* [64], *Ph*. *sergenti* [73], *Ph*. *perniciosus* [82], and *Ph*. *longicuspis* [73], Naples viruses in *Ph*. *perniciosus* and *Ph*. *longicuspis* [82,87], and Sicilian virus in *Ph*. *ariasi* [93]. This list is far from being exhaustive, and it is expected that other sand fly species, especially of the subgenus *Larroussius*, may be incriminated in circulation of these viruses [144]. Some of these viruses were detected in other sand fly species in neighboring countries, for example, Sicilian virus in *Ph*. *papatasi* in Egypt [63]. Sometimes, a virus is detected in pooled sand flies without their exact species identification, relying on parallel morphological identification of a subset of sand fly catch to estimate the relative abundance of each sand fly species; this was the case of TOSV detection in Algeria with 6 sand fly species known to occur in the focus [145] and in Tunisia where *Ph*. *perniciosus* was the most abundant species [146]. NGS and statistical analysis of the subsequent reads would enable species identification within pooled samples; however, such analysis is not always available. The diversity of phleboviruses in the Maghreb region is still not fully understood, and several novel viruses were detected or isolated from sand flies, such as Punique virus [82] and Saddaguia virus [147] detected in sand flies in Tunisia. To determine the involvement of different sand fly species in their transmission remains one of the future research tasks.

While species identification based on a detailed analysis of decisive morphological features remains a golden standard in sand fly taxonomy, the advent of molecular methods in last decades allowed it to be complemented by several other approaches. DNA-based techniques targeting various genetic markers such as Cytochrome b (Cytb), Cytochrome C oxidase subunit I (COI), and Internal Transcribed Spacer (ITS) regions allowed further insight into sand fly taxonomy, revealing the presence of *Ph*. *perniciosus* atypical form so long confused with *Ph*. *longicuspis*, a closely related species [129,130], boosting morphological identification criteria proposed for the couple *Ph*. *chabaudi*/*Ph*. *riouxi*, 2 closely related and morphologically challenging species [15,48]. In another study assessing the status of *Se*. *minuta* populations from different parts of the Mediterranean Basin, the analysis of COI and Cytb sequences allowed to reveal the presence of a complex genetic structure including 2 subpopulations *Se*. *minuta minuta* in Europe and *Se*. *minuta parroti* in North Africa [148]. Moreover, molecular techniques proved useful for species identification of pooled sand fly specimens in studies aiming to detect arboviruses where NGS analysis and statistical analysis of the subsequent reads was used to help species identification within pooled samples [149–151]. Recently, protein-based method of MALDI-TOF, which allows a rapid and cost-effective species identification of sand flies [152], was successfully implemented in sand fly surveys in Algeria and Morocco [153,154]. Both DNA and protein-targeting molecular methods were successfully combined with morphological analysis in other geographical regions [155,156] and may be applied in the Maghreb countries to resolve taxonomical issues such as species status confirmation of *Se*. *hirtus* or *Se*. *africana* subsp. *eremites* and *Se*. *africana* subsp. *asiatica*, which were considered as synonymous [157]. In this respect, updated and detailed knowledge of the sand fly fauna as summarized in this study may be a valuable background for further studies.

In conclusion, this review summarizes the sand fly fauna occurring in the Maghreb region, compiling the distribution maps of each species based on published findings, discussing the suspected or proven role of each species in the transmission of pathogens, providing species-specific morphological features for the species identification, and highlighting the gaps to be filled regarding the vectorial status and presence of some sand fly species, pointing out a complete absence of recent data from Mauritania and fragmented knowledge of sand fly fauna in Libya, a country suffering a prolonged political unrest that disrupted healthcare system and sand fly control programs.

**Key Learning Points**

- Leishmaniases remain a major public health problem in the Maghreb region.
- Current gaps of knowledge highlight the necessity to establish or strengthen the entomological networks to study sand fly fauna, species distribution, and their involvement in transmission of pathogens in the Maghreb region.
- Sand fly–borne viruses and the diseases they may cause deserve more attention in this region where different phleboviruses were reported.
- There is an urgent need to fulfill the data gaps concerning leishmaniases and the sand fly vectors in Mauritania.
- Taxonomy of several sand fly species shall be addressed by integrating both morphological and molecular approaches and implementing sustained field research.

**Top Five Papers**

1. Maroli M, Feliciangeli MD, Bichaud L, Charrel RN, Gradoni L. Phlebotomine sandflies and the spreading of leishmaniases and other diseases of public health concern. Med Vet Entomol. 2013;27(2):123–47.
2. Alvar J, Vélez ID, Bern C, Herrero M, Desjeux P, Cano J, et al. Leishmaniasis worldwide and global estimates of its incidence. PLoS ONE. 2012;7(5):35671. Available from: www.plosone.org
3. Swynghedauw B, Weméau JL. Consequences of climate change on human and animal health. Bull Acad Natl Med. 2021;205:219–26.
4. Dvorak V, Halada P, Hlavackova K, Dokianakis E, Antoniou M, Volf P. Identification of phlebotomine sand flies (Diptera: Psychodidae) by matrix-assisted laser desorption/ionization time of flight mass spectrometry. Parasit Vectors. 2014 Jan 14;7(1).
5. Depaquit J. Molecular systematics applied to Phlebotomine sandflies: Review and perspectives. Infect Genet Evol. 2014;28:744–56. doi: 10.1016/j.meegid.2014.10.027

## Supporting information

S1 Data Geographic location of sand fly species in Maghreb region. (XLS)

S1 Text Main differences between *Phlebotomus* and *Sergentomyia* species. (DOCX)
