# Supplementary material for: Phlebotomine sand flies (Diptera: Psychodidae) of the Maghreb region: A systematic review of distribution, morphology, and role in the transmission of the pathogens
Source: PLoS Negl Trop Dis. 2022 Jan 6;16(1):e0009952. doi: 10.1371/journal.pntd.0009952 (PMC8735671; doi:10.1371/journal.pntd.0009952)
Supplement: S1 Text — (DOCX) [file pntd.0009952.s002.docx]

Table A: Morphological features of males within the genus *Phlebotomus*

| Species | Feature | | | | | |  |
| --- | --- | --- | --- | --- | --- | --- | --- |
|  | Number of spines on the style | Tuft the coxite | Basal lobe | Aedeagus shape | Cibarial armature | Other |  |
| *Phlebotomus perniciosus* | Style holding five dispersed spines | 10-18 | Absent | Long and forked at the tip | Absent or rudimentary | / |  |
| *Phlebotomus perniciosus atypical* |  | 10-16 |  | Long and curved at the tip with one point |  |  |  |
| *Phlebotomus longicuspis* |  | 18-32 |  | Long and slightly curved with one point |  |  |  |
| *Phlebotomus langeroni* |  | 21 |  | Long and bevel-shaped of the tip |  |  |  |
| *Phlebotomus perfiliewi* |  | / |  | Long and thin with a blunt and 4-6 denticules at the tip |  |  |  |
| *Phlebotomus ariasi* |  | 30-35 |  | Long, baseball bat-like and thin with a blunt |  |  |  |
| *Phlebotomus chadlii* |  | 60-80 |  | Short, baseball bat-like and thin with a blunt |  |  |  |
| *Phlebotomus mascitti* |  | / |  | Distal region tapered and cup-like expansion at the anterior end |  | Genital pump thin-walled and colourless |  |
| *Phlebotomus mariae* |  | 25-30 |  | Long, narrow and a little bit swelled at the tip |  |  |  |
| *Phlebotomus papatasi* | Long and thick style holding five spines: three at the middle and the two distal part | 10-12 | Poorly developed | Short and conical |  | Style 400 µm |  |
| *Phlebotomus bergeroti* |  | 6-7 |  |  |  | Style 250 µm |  |
| *Phlebotomus sergenti* | Four spines, two distal and two in the middle | / | Well-developed and simple, 20 µm |  |  | Genital pump 130-160 µm |  |
| *Phlebotomus alexandri* |  |  | Well-developed and simple, 10-15 µm |  |  | Genital pump 80-90 µm |  |
| *Phlebotomus kazeruni* |  |  | Very small and narrow |  |  | Genital pump 160 µm |  |
|  |  |  |  |  |  |  |  |
| *Phlebotomus chabaudi* |  |  | Well-developed  28µm length/10 setae | Short and pointed |  |  |  |
| *Phlebotomus riouxi* |  |  | Well-developed  54µm length/23-40 setae |  |  |  |  |

Table B: Morphological features of females within the genus *Phlebotomus*

| Species | Feature | | |
| --- | --- | --- | --- |
|  | Spermathecae | Pharynx | Others |
| *Phlebotomus perniciosus* | 6-13 segments with a long neck. The walls are very thick and refractive. The light is narrow, conical, very pointed, opening through a circular orifice in the sub-terminal part of the duct | armature composed by posterior transverse rows of combs and in front of thread-like denticules (1/3 of the pharynx), arranged in small groups and descending more on the edges than in the centre. |  |
| *Phlebotomus longicuspis* | 10-12 segments with a long neck. A sub bulb is attached to the striated duct of the spermathecae, slightly bilobed, thick-walled terminal | A quarter of the total length with transverse rows of small combs, the anterior combs having more marked teeth than the posterior ones. |  |
| *Phlebotomus langeroni* | 9-14 well-defined segments, those in middle larger than end segments; apical knob setose at end of long slender neck; neck about  2/3 length of spermathecae body; individual ducts of uniform diameter until terminal 1/3, where they widen progressively |  |  |
| *Phlebotomus perfiliewi* | 10-21 segments with a short neck. The duct is striated and it opens into a large asymmetrical pocket, with thick and wrinkled walls. The two pockets, independent of each other, open side by side in the genital atrium, through large openings | The narrow posterior part is furnished with substantially horizontal rows of shorter denticules at the edges than in the middle region. The insertion of the first row of teeth appears clearly perpendicular to the longitudinal axis of the pharynx (1/5 of the pharynx) |  |
| *Phlebotomus ariasi* | 10-14 regular segments with a short neck. Duct slightly striated and widely dilated in its proximal part as a honeycomb-like structure. | Armed on a surface to approximately 1/5 of the total length, punctiform denticules irregularly arranged in front and transverse rows of small finely denticulate projections |  |
| *Phlebotomus chadlii* | Thirteen irregular segments (the first and the last segments are large than the rest) with a short neck. Duct smooth and widely dilated in its proximal smooth part. | Armed on a surface to approximately 1/3 of the total length, punctiform denticules irregularly arranged in front and transverse rows of small finely denticulate projections |  |
| *Phlebotomus mariae* | Not known yet |  |  |
| *Phlebotomus mascitti* | Not ringed and transversal striations in the distal part. No neck and wide ducts | Irregular teeth |  |
| *Phlebotomus papatasi* | 6-9 segments without neck | Weakly distended posterior part, armed in its posterior part with a network of strong scaly teeth very irregular and irregularly arranged | Ascoids of  A**IV** asymmetric & shorter |
| *Phlebotomus bergeroti* |  | Weakly distended posterior part like a bottle shape, armed in its posterior part with a network of strong scaly teeth very irregular and irregularly arranged | Ascoids of  A**IV** symmetric & long |
| *Phlebotomus sergenti* | 3-7 segment with dilated apical segment and without neck | 1/5 posterior pharynx armed with a network of strong scaly teeth regularly arranged | AI**II** 250-290 µm |
| *Phlebotomus alexandri* | 5-7 segment with dilated apical segment and without neck | Posterior pharynx armed with a network of strong scaly teeth regularly arranged and truncated at the posterior end. | A**III** 182-200 µm |
| *Phlebotomus kazeruni* | 1-2 segment with dilated apical segment and without neck | 1/5 posterior pharynx armed with a network of strong scaly teeth regularly arranged | A**III** 240 µm |
| *Phlebotomus chabaudi* | 6-8 segments with a collarets | 1/3 posterior pharynx armed with a network of strong scaly teeth regularly arranged | A**III** 300-360 µm |
| *Phlebotomus riouxi* | 6-8 segments with a collarets | 1/3 posterior pharynx armed with a network of strong scaly teeth regularly arranged | A**III** 223-314 µm |

Table C: main differences between *Phlebotomus papatasi* and *Ph. bergeroti.*

| Criterion | *Phlebotomus papatasi* | | *Phlebotomus bergeroti* | |
| --- | --- | --- | --- | --- |
|  | Male | Female | Male | Female |
| Pharynx shape, Fig 1a: A&B | / | Weakly distended posterior part | / | Weakly distended posterior part like a bottle shape |
| Ascoids shape and length compared to the 4^th^ antennal segment  Fig 1a: C&D | / | Asymmetric & shorter | / | Symmetric & longer |
| Basal lobe coxite setae, Fig 1b:C&F | 10 | / | 6 | / |
| The length between the apical and median spines, Fig 1b: A&D | Long | / | Short | / |
| The shape of the spines, Fig 1b: A&D | Sharp | / | Spatulate | / |
| Tuft hair of the coxite, Fig 1b:C&F | More than 12 | / | Less than 10 | / |

Table D: Morphological features of males within the *Sergentomyia*.

| Species | Feature | | | | |  |
| --- | --- | --- | --- | --- | --- | --- |
|  | Number of the style spines and arrangement | Non-deciduous seta of the style | Aedeagus shape | Cibarial armature | Others |  |
| *Sergentomyia minuta* | Four distal spines | Forward to the middle | Thick and finger-like with a blunt tip | On a straight or curved line | / |  |
| *Sergentomyia antennata* |  | On the distal 1/3 |  | 16-18 teeth, on a posterior concave line | Antenna **AIII** 90**-**110µm |  |
| *Sergentomyia fallax* |  |  |  |  | Long and thin style |  |
| *Sergentomyia dreyfussi* |  |  | Long and conical with a pointed tip | 20 separated teeth | Leg 1: presence of spines on the femur |  |
| *Sergentomyia cincta* |  |  | Thick and finger-like with a blunt tip | 12-18 teeth, on a posterior concave line | Antenna **AIII** 80**-**120µm |  |
| *Sergentomyia schwetzi* | Distal spines arranged in two groups well separated |  | Thick and finger-like with a blunt tip | Two rows of short and sharped teeth (15-20 and 10-15) | / |  |
| *Sergentomyia lewisi* | Four distal spines | On the proximal 1/3 forward the middle | Long and conical with a blunt tip | 8-12 teeth, on a posterior concave line, 8-14 denticules | Genital pump without pavilion |  |
| *Sergentomyia africana*  subsp *asiatica* |  |  | Long and conical with a pointed tip | 17-30 teeth on a palisade line 6-18 denticles | Antenna **AIII** 150**-**230µm |  |
| *Sergentomyia africana subsp eremitis* |  |  |  | 30-35 teeth on a concave line, 8-10 denticles | Antenna **AIII** 130**-**160µm |  |
| *Sergentomyia christophersi* | Two terminal and two sub-terminal spines |  | Short and conical | 2-3 short and strong teeth | Antenna **AIII** 110-120 µm |  |
| *Sergentomyia clydei* |  |  |  | 2-3 short and strong teeth and 25-35 denticules | Antenna **AIII** 110-150 µm |  |
| *Sergentomyia tiberiadis* | Four distal spines | On the distal 1/3 | elongated and very sharp point | 12-14 sharp teeth | Antenna **AIII** 120**-**160µm |  |
| *Sergentomyia hirtus* | Seven spines | On the distal 1/3 forward the middle | Short enough and subconical | Two rows of short and sharped teeth | Antenna **AIII** 0.140µm |  |

Table E: Morphological features of females within the *Sergentomyia*.

| Species | Feature | | | |
| --- | --- | --- | --- | --- |
|  | Spermathecae | Cibarium | Pharynx | Others |
| *Sergentomyia minuta minuta* | Smooth | Average of 40 small teeth deposed in palisade and headband pigmented patch | Narrow posterior part (lamp glass shaped) | Antenna **AIII** 70-95 µm |
| *Sergentomyia minuta parroti* |  | Average of 70 (60-100) small teeth deposed in palisade and headband pigmented patch |  | Antenna **AIII** 120-170 µm |
| *Sergentomyia fallax* |  | 16-18 sharped teeth aligned on concave shape and oval or rounded pigmented patch | Strongly distended posterior part heart-shaped | Antenna **AIII** 100-150 µm |
| *Sergentomyia antennata* |  | 22-26 sharped teeth aligned on a concave shape and triangular pigmented patch | Less large posterior part and heart-shaped | Antenna **AIII** 90-100 µm |
| *Sergentomyia cincta* |  | 14-18 sharped teeth strong interiorly concave line, mushroom-shaped pigmented patch | distended posterior part heart-shaped | Antenna **AIII** 78-88 µm |
| *Sergentomyia schwetzi* |  | 13-20 strong and sharped teeth aligned on concave shape with pigment patch large in the middle | Narrow and strong-armed posterior part (lamp glass shaped) | Antenna **AIII** 180- 250 µm |
| *Sergentomyia christophersi* | 7-8 segments without neck | 4-7 sharped pointed teeth, 4-5 denticules and small triangular pigmented patch | Weakly distended and strongly armed posterior part | Antenna **AIII** 115- 128 µm |
| *Sergentomyia clydei* |  | Two rows of teeth: 12-13 long and sharped, and 16-18 short and blunt, diamond pigmented patch | Weakly distended and armed posterior part | Antenna **AIII** 130- 160 µm |
| *Sergentomyia* *tiberiadis* | 6-8 segments without neck | 18 sharped teeth, the lateral ones are more large than the middle ones, two rows of denticules (14-16 and 10-12), mushroom-shaped pigmented patch | Unarmed | Antenna **AIII** 130- 180 µm |
| *Sergentomyia dreyfussi* | Rounded capsule almost completely covered by thin spicules | 42-55 sharped teeth aligned on a concave shape, 36-40 denticules, and ping pong bat pigmented patch | Strongly distended posterior part heart-shaped | Leg 1: 8-9 spines on the femur; Leg 2: 4-5 spines on the femur; Leg 3: 3-4 spines on the femur.  Antenna **AIII** 170- 210 µm |
| *Sergentomyia lewisi* | Lamp glass shaped capsule | 15-20 strong and sharped teeth aligned on concave, 16-20 denticules, mushroom-shaped pigmented patch | Weakly distended and strongly armed posterior part | Antenna **AIII** 170- 220 µm |
| *Sergentomyia africana* subsp *asiatica* | Elongated capsule | 40-58 teeth aligned in palisade, headband pigmented patch | Consists of 15 sharped denticules, long and thread-like. | Antenna **AIII** 120- 130 µm |
| *Sergentomyia africana* subsp *eremitis* |  | 60-65 long teeth aligned on concave and 12 punctiform denticules, mushroom-shaped pigmented patch | Weakly distended and armed posterior part | Antenna **AIII** 110- 140 µm |
| *Sergentomyia hirtus* | Not known yet | | | |
